# Nested soft-collinear subtractions in NNLO QCD computations

**DOI:** 10.1140/epjc/s10052-017-4774-0

**Published:** 2017-04-18

**Authors:** Fabrizio Caola, Kirill Melnikov, Raoul Röntsch

**Affiliations:** 1 CERN, Theoretical Physics Department, Geneva, Switzerland; 2 IPPP, Durham University, Durham, UK; 3 Institute for Theoretical Particle Physics, KIT, Karlsruhe, Germany

## Abstract

We discuss a modification of the next-to-next-to-leading order (NNLO) subtraction scheme based on the residue-improved sector decomposition that reduces the number of double-real emission sectors from five to four. In particular, a sector where energies and angles of unresolved particles vanish in a correlated fashion is redundant and can be discarded. This simple observation allows us to formulate a transparent iterative subtraction procedure for double-real emission contributions, to demonstrate the cancellation of soft and collinear singularities in an explicit and (almost) process-independent way and to write the result of a NNLO calculation in terms of quantities that can be computed in four space-time dimensions. We illustrate this procedure explicitly in the simple case of $\mathcal O(\alpha _\mathrm{s}^2)$ gluonic corrections to the Drell–Yan process of $q \bar{q}$ annihilation into a lepton pair. We show that this framework leads to fast and numerically stable computation of QCD corrections.

## Introduction

One of the most important recent advances in perturbative QCD was the discovery of practical ways to perform fully differential next-to-next-to-leading order (NNLO) QCD computations for hadron collider processes. These methods, which include antenna [1–9], residue-improved sector-decomposition [10–12] (see also [13]) and projection to Born [14] subtraction schemes, as well as $q_\perp$ [15, 16] and *N*-jettiness [17, 18] slicing methods, were used to perform an impressive number of NNLO QCD computations relevant for LHC phenomenology [14, 15, 18–62].^1^

However, in spite of these remarkable successes, it is important to recognize that existing implementations of subtraction schemes are complex, not transparent, and require significant CPU time to produce stable results. On the other hand, slicing methods, while conceptually simple, have to be carefully controlled to avoid dependence of the final result on the slicing parameter. Given these shortcomings, it is important to study whether improvements to existing methods are possible. In the context of the *N*-jettiness slicing method, there has been recent progress toward a better control of the soft-collinear region [65, 66].

In this paper we study the residue-improved subtraction scheme introduced in Refs. [10–12]. This scheme is interesting because it is the only existing framework for NNLO QCD computations that is fully local in multi-particle phase space. As such, it should demonstrate exemplary numerical stability, at least in theory. Although this scheme is well understood and was applied to a large number of non-trivial problems, we will argue in this paper that certain aspects of it are redundant. Interestingly, once this redundancy is recognized and removed, the residue-improved subtraction scheme becomes very transparent and physical. In addition, the technical simplifications that occur become so significant that the cancellation of the divergent terms can be demonstrated independently of the hard matrix element and almost entirely analytically, and the final finite result for the NNLO contribution to (in principle) any process can be written in a compact form in terms of *generic* four-dimensional matrix elements.^2^

Although the improvements that we just described hold true for an arbitrary complicated process, in this paper, for the sake of clarity, we restrict our discussion to the production of a colorless final state in $q \bar{q}$ annihilation. This allows us to discuss all the relevant conceptual and technical aspects of the computational framework, without cluttering the notation and limiting the bookkeeping to a minimum. The generalization of the framework described here to arbitrary processes is – at least conceptually – straightforward.

Admittedly, compared to NNLO QCD problems studied recently, the production of a colorless final state in $q \bar{q}$ annihilation is a very simple process, which has been discussed in the literature many times. However, we believe that the simplicity of our approach and the structures that emerge justify revisiting it one more time. Moreover, thanks to the simplicity of this process, we will be able to describe our approach in detail and demonstrate many intermediate steps of the calculation. Hopefully this will allow us to make the rather technical subject of NNLO subtractions accessible to a broader part of the particle physics community.

The paper is organized as follows. We begin with preliminary remarks in Sect. 2, where we also precisely define the problem that we plan to address. In Sect. 3 we discuss the next-to-leading order (NLO) QCD computation as a prototype of the following NNLO QCD construction. In Sect. 4, we describe how the NLO computation generalizes to the NNLO case. We elaborate on this in Sect. 5, where we discuss ultraviolet and collinear renormalization, and in Sects. 6, 7 and 8, where we study two-loop virtual corrections, one-loop corrections to single-real emission process, and the double-real emission contributions, respectively. In Sect. 9, we combine the different contributions and present the final result for the NNLO QCD corrections to color singlet production in $q\bar{q}$ annihilation. In Sect. 10, we show some numerical results and a comparison with earlier analytic calculations. We conclude in Sect. 11. A collection of useful formulas is provided in the appendices.

## Preliminary remarks

We consider the production of a colorless final state *V* in the collision of two protons

2.1 $$\begin{aligned} P+P\rightarrow V + X. \end{aligned}$$

We are interested in computing the differential cross section for the process in Eq. (2.1)

2.2 $$\begin{aligned} \mathrm{d} \sigma = \sum \limits _{ij}^{} \int \mathrm{d} x_1 \mathrm{d}x_2 f_i (x_1) f_j(x_2) \mathrm{d}\hat{\sigma }_{ij}(x_1, x_2), \end{aligned}$$

where $\mathrm{d}\hat{\sigma }_{ij}$ is the finite partonic scattering cross section, $f_{i,j}$ are parton distribution functions and $x_{1,2}$ are momenta fractions of the incoming hadrons that are carried to a hard collision by partons *i* and *j*, respectively. The dependence on the renormalization and factorization scales and all other parameters of the process is understood. The finite partonic scattering cross section is obtained after the renormalization of the strong coupling constant removes all ultraviolet divergences, all soft and final state collinear divergences cancel in the sum of cross sections with different partonic multiplicities, and the initial state collinear divergences are subtracted by redefining parton distribution functions.

Since the process under consideration is driven by a conserved current that is independent of $\alpha _\mathrm{s}$, the ultraviolet renormalization reduces to the following ($\overline{\mathrm{MS}}$) redefinition of the strong coupling constant:

2.3 $$\begin{aligned} \frac{\alpha _{\mathrm{s},b}}{2\pi } (\mu _0^2)^{\epsilon }S_{\epsilon }= \frac{\alpha _\mathrm{s}(\mu )}{2\pi }\mu ^{2\epsilon } \left[ 1-\frac{\beta _0}{\epsilon }\frac{\alpha _\mathrm{s}(\mu )}{2\pi }+ \mathcal O\left( \alpha _\mathrm{s}^2\right) \right] , \end{aligned}$$

where $S_\epsilon = (4\pi )^\epsilon e^{-\epsilon \gamma _\mathrm{E}},~\gamma _\mathrm{E}\approx 0.577216$ is the Euler–Mascheroni constant, $\epsilon =(4-d)/2$ and

2.4 $$\begin{aligned} \beta _0 = \frac{11}{6}C_\mathrm{A}- \frac{2}{3} T_\mathrm{R} n_f,\quad C_\mathrm{A}= 3,~T_\mathrm{R} = \frac{1}{2}, \end{aligned}$$

is the leading-order (LO) QCD $\beta$-function.

Collinear divergences associated with initial state QCD radiation are removed by a redefinition of parton distributions. In the $\overline{\mathrm{MS}}$ scheme, this amounts to the replacement

2.5 $$\begin{aligned} f_{i,b}&\rightarrow \bigg [1 + \frac{\alpha _\mathrm{s}(\mu )}{2\pi }\frac{\hat{P}^{(0)}_{ij}}{\epsilon }+ \left( \frac{\alpha _\mathrm{s}(\mu )}{2\pi }\right) ^2 \nonumber \\&\quad \times \left[ \frac{1}{2\epsilon ^2}\left( \hat{P}^{(0)}_{ik} \otimes \hat{P}^{(0)}_{kj} - \beta _0 \hat{P}^{(0)}_{ij} \right) + \frac{1}{2\epsilon } \hat{P}^{(1)}_{ij} \right] \nonumber \\&\quad + \mathcal O(\alpha _\mathrm{s}^3) \bigg ]\otimes f_j (\mu ), \end{aligned}$$

where $\otimes$ stands for the convolution

2.6 $$\begin{aligned} g(z) = [ f_1 \otimes f_2 ](z)= \int \limits _{0}^{1} \mathrm{d} x \mathrm{d} y f_1(x) f_2(y) \delta (z-x y), \end{aligned}$$

and $\hat{P}_{ij}^{(0,1)}$ are the Altarelli–Parisi splitting functions.

As we already mentioned, we focus on gluonic corrections to the $q\bar{q}$ annihilation channel,

2.7 $$\begin{aligned} q+\bar{q} \rightarrow V + ng. \end{aligned}$$

This allows us to present all the features of the framework while limiting the bookkeeping to a minimum and, therefore, to keep the discussion relatively concise. All other partonic channels relevant for the Drell–Yan process can be obtained by a simple generalization of what we will describe.

The collinear-renormalized partonic cross section for $q \bar{q}$ annihilation into a vector boson is expanded in series of $\alpha _\mathrm{s}$. We write

2.8 $$\begin{aligned} \mathrm{d}\hat{\sigma }\equiv \mathrm{d}\hat{\sigma }_{q\bar{q}}= \mathrm{d} \hat{\sigma }^\mathrm{LO} + \mathrm{d} \hat{\sigma }^\mathrm{NLO} + \mathrm{d} \hat{\sigma }^\mathrm{NNLO}, \end{aligned}$$

where

2.9 $$\begin{aligned}&\mathrm{d}\hat{\sigma }^\mathrm{NLO} = \mathrm{d} \sigma ^\mathrm{V} + \mathrm{d} \sigma ^\mathrm{R}\nonumber \\&\quad \quad \quad \quad \quad + \frac{\alpha _\mathrm{s}(\mu )}{2 \pi \epsilon } \left( \hat{P}_{qq}^{(0)} \otimes \mathrm{d} {\hat{\sigma }^\mathrm{LO}} + \mathrm{d} \hat{\sigma }^\mathrm{LO} \otimes \hat{P}_{qq}^{(0)} \right) , \nonumber \\&\mathrm{d}\hat{\sigma }^\mathrm{NNLO} = \mathrm{d} \sigma ^\mathrm{VV} + \mathrm{d} \sigma ^\mathrm{RV} + \mathrm{d} \sigma ^\mathrm{RR} + \mathrm{d}\sigma ^\mathrm{ren} + \mathrm{d} \sigma ^\mathrm{CV}. \end{aligned}$$

Various contributions in Eq. (2.9) refer to virtual and real corrections, as well as to contributions to cross sections that arise because of the ultraviolet and collinear renormalizations. The latter are obtained with the procedure just described and read

2.10 $$\begin{aligned} \mathrm{d}\sigma ^\mathrm{ren}&= -\frac{\alpha _\mathrm{s}(\mu )}{2\pi }\frac{\beta _0}{\epsilon } \mathrm{d}\hat{\sigma }^\mathrm{NLO},\nonumber \\ \mathrm{d}\sigma ^\mathrm{CV}&= \frac{\alpha _\mathrm{s}(\mu )}{2\pi }\bigg [ \Gamma _1 \otimes \mathrm{d}\hat{\sigma }^\mathrm{NLO} + \mathrm{d}\hat{\sigma }^\mathrm{NLO}\otimes \Gamma _1\bigg ] -\left( \frac{\alpha _\mathrm{s}(\mu )}{2\pi }\right) ^2 \nonumber \\&\quad \times \bigg [ \Gamma _1\otimes \mathrm{d}\hat{\sigma }^\mathrm{LO}\otimes \Gamma _1 +\Gamma _2\otimes \mathrm{d}\hat{\sigma }^\mathrm{LO} +\mathrm{d}\hat{\sigma }^\mathrm{LO}\otimes \Gamma _2 \bigg ], \end{aligned}$$

where

2.11 $$\begin{aligned} \Gamma _1 = \frac{\hat{P}_{qq}^{(0)}}{\epsilon },\quad \Gamma _2 = \frac{\hat{P}_{qq}^{(0)}\otimes \hat{P}_{qq}^{(0)}+\beta _0 \hat{P}_{qq}^{(0)}}{2\epsilon ^2} -\frac{\hat{P}^{(1)}_{qq}}{2\epsilon } ,\nonumber \\ \end{aligned}$$

and the relevant splitting functions are provided in Appendix A.

The cross section $\mathrm{d} \hat{\sigma }^\mathrm{(N)NLO}$ is finite but all the individual contributions in Eq. (2.9) are divergent. The well-known problem is that these divergences are explicit in some of the terms and implicit in the others. Indeed, soft and collinear divergences appear as explicit $1/\epsilon$ poles in virtual corrections but they only become evident in real corrections once integration over gluon momenta is performed. However, since we would like to keep the kinematics of all the final state particles intact, we cannot integrate over momenta of any of the final state particles if it is resolved. It is this point that makes extraction of implicit singularities complicated and requires us to devise a procedure to do it.

Depending on how these implicit singularities are extracted, it may or may not be straightforward to recognize how they combine and cancel, once all contributions to the physical cross section are put together. At NNLO, this was done for the antenna subtraction scheme and, in a less transparent way, for the residue-improved sector decomposition. One thing we would like to do, therefore, is to combine the individual terms that contribute to partonic cross sections, and cancel all the $1/\epsilon$ divergences explicitly, without any reference to the matrix elements that contribute to the different terms in Eq. (2.9). In the next section we show how to do that at next-to-leading order in the perturbative expansion for the Drell–Yan process. This will allow us to set up the formalism and the notation that will be used for the NNLO analysis of Sects. 4–8.

## The NLO calculation

We will illustrate our approach by studying the production of a lepton pair in quark–antiquark annihilation at next-to-leading order in perturbative QCD. We note that, at this order, the method that we would like to describe is identical to the FKS subtraction scheme introduced in Refs. [67, 68]. However, we formulate the FKS method in a way that makes its extension to next-to-next-to-leading order as straightforward as possible.^3^ One point that we found helpful, especially for bookkeeping, was to introduce soft and collinear subtraction operators, and we show how to use them in the NLO computation below.

We are interested in the calculation of the finite partonic cross section $\mathrm{d}\hat{\sigma }^\mathrm{NLO}$ defined in Eq. (2.9). It receives contributions from the virtual and real corrections and the collinear subtraction term. We will start the discussion with the real-emission contribution. It refers to the process

3.1 $$\begin{aligned} q(p_1) + \bar{q}(p_2) \rightarrow V + g(p_4), \end{aligned}$$

where *V* is a generic notation for all colorless particles in the final state. We write the cross section for the process in Eq. (3.1) as

3.2 $$\begin{aligned} \mathrm{d} \sigma ^\mathrm{R} = \frac{1}{2 s}\int [\mathrm{d}g_{4}] F_{LM}(1,2,4), \end{aligned}$$

where *s* is the partonic center-of-mass energy,

3.3 $$\begin{aligned}{}[\mathrm{d}g_{4}] = \frac{\mathrm{d}^{d-1} p_4}{(2\pi )^d 2 E_4} \theta (E_\mathrm{max}-E_4), \end{aligned}$$

and

3.4 $$\begin{aligned} F_{LM}(1,2,4) = \mathrm{d}\mathrm{Lips}_V\; |\mathcal{M}(1,2,4,V)|^2\; \mathcal{F}_\mathrm{kin}(1,2,4,V). \end{aligned}$$

In Eq. (3.4), $\mathrm{d}\mathrm{Lips}_V$ is the Lorentz-invariant phase space for colorless particles, including the momentum-conserving $\delta ^{(d)}(p_1+p_2-p_4-p_V)$, $\mathcal{M}(1,2,4,V)$ is the matrix element for the process in Eq. (3.1) and $\mathcal{F}_\mathrm{kin}(1,2,4,V)$ is an (infra-red safe) observable that depends on kinematic variables of all particles in the process. Also, $E_\mathrm{max}$ is an arbitrary auxiliary parameter that has to be large enough to accommodate all possible kinematic configurations for $q \bar{q} \rightarrow V+g$. The need to introduce such a parameter is a consequence of our construction, as explained in detail below.

We would like to isolate and extract soft and collinear singularities that appear when the integration over $[\mathrm{d}g_{4}]$ in Eq. (3.2) is attempted. To this end, we introduce two operators that define soft and collinear projections

3.5 $$\begin{aligned} S_i A = \lim _{E_i \rightarrow 0} A,\quad C_{ij} A = \lim _{\rho _{ij} \rightarrow 0} A, \end{aligned}$$

where $\rho _{ij} = 1 - n_{i} \cdot n_{j}$ and $n_{i}$ is a unit vector that describes the direction of the momentum of the *i*th particle in $(d-1)$-dimensional space. By definition, operators in Eq. (3.5) act on everything that appears to the right of them. The limit operations, on the right hand side of Eq. (3.5), are to be understood in the sense of extracting the most singular contribution provided that limits in the conventional sense do not exist. We will also use the averaging sign $\langle ... \rangle$ to represent integration over momenta of final state particles. This integration is supposed to be performed in the center-of-mass frame of incoming partons. *We emphasize that this remark is important since our construction of the subtraction terms is frame-dependent and not Lorentz-invariant.*

We rewrite Eq. (3.2) in the following way:

3.6 $$\begin{aligned}&\int [ \mathrm{d} g_4] F_{LM}(1,2,4) \nonumber \\ {}&\quad = \langle F_{LM}(1,2,4) \rangle = \langle S_4 F_{LM}(1,2,4) \rangle +\langle ( I - S_4) F_{LM}(1,2,4) \rangle \nonumber \\&\quad = \langle S_4 F_{LM}(1,2,4) \rangle + \langle (C_{41} + C_{42} ) ( I - S_4) F_{LM}(1,2,4) \rangle \nonumber \\&\quad \quad + \langle \hat{O}_\mathrm{NLO} F_{LM}(1,2,4) \rangle , \end{aligned}$$

where *I* is the identity operator and $\hat{O}_\mathrm{NLO}$ is a short-hand notation for a combination of soft and collinear projection operators

3.7 $$\begin{aligned} \hat{O}_\mathrm{NLO} = ( I - C_{41} - C_{42} ) ( I - S_4). \end{aligned}$$

Note that in Eq. (3.6) soft and collinear projection operators act on $F_{LM}(1,2,4)$, which, according to Eq. (3.4), contains the energy-momentum conserving $\delta$-function; we stress that the soft and collinear limits must be taken in that $\delta$-function as well.

The reason for rewriting $\mathrm{d} \sigma ^\mathrm{R}$ as in Eq. (3.6) is that the last term there is finite, thanks to the nested structure of subtraction terms. This term can, therefore, be integrated numerically in four dimensions. We emphasize again that the subtraction terms, as formulated here, are not Lorentz-invariant. This means that all the three terms in Eq. (3.6) should be computed in the same reference frame that, as already mentioned, is taken to be the center-of-mass reference frame of the colliding partons.

We now consider the remaining two terms in Eq. (3.6). Their common feature is either complete or partial decoupling of the gluon $g_4$ from the matrix element thanks to the fact that they contain either soft or collinear projection operators. Hence, those terms can be re-written in such a way that all singularities are extracted and canceled, without specifying the matrix elements for the hard process.

To see this explicitly, consider first two terms in Eq. (3.6) and write them as

3.8 $$\begin{aligned} \big \langle ( I - C_{41} - C_{42} ) S_4 F_{LM}(1,2,4) \big \rangle + \big \langle (C_{41} + C_{42} ) F_{LM}(1,2,4) \big \rangle . \end{aligned}$$

It is easy to see that the first term in Eq. (3.8) vanishes.^4^ Indeed, in the limit when the gluon $g_4$ becomes soft, we find

3.9 $$\begin{aligned} S_4 F_{LM}(1,2,4) = \frac{2 C_\mathrm{F}g_{s,b}^2 }{E_4^2} \frac{\rho _{12}}{\rho _{14} \rho _{24} } F_{LM}(1,2), \end{aligned}$$

where $g_{s,b}$ is the bare QCD coupling, $C_\mathrm{F}=4/3$ is the QCD color factor, and $F_{LM}(1,2)$ is closely related to the LO cross section

3.10 $$\begin{aligned} \langle F_{LM}(1,2) \rangle= & {} 2s \cdot \mathrm{d} \hat{\sigma }^\mathrm{LO} \nonumber \\= & {} \int \mathrm{d}\mathrm{Lips}_V\; |\mathcal{M}(1,2,V)|^2\; \mathcal{F}_\mathrm{kin}(1,2,V). \nonumber \\ \end{aligned}$$

The action of the collinear operators on the $\rho$ gives

3.11 $$\begin{aligned} C_{41} \frac{\rho _{12}}{\rho _{14}\rho _{24}} = \frac{1}{\rho _{14}},\quad C_{42} \frac{\rho _{12}}{\rho _{14}\rho _{24}} = \frac{1}{\rho _{24}}. \end{aligned}$$

Since for head-on collision $\rho _{12}=2$, $\rho _{24}=2-\rho _{14}$, we find

3.12 $$\begin{aligned} \frac{\rho _{12}}{\rho _{14}\rho _{24}} = \frac{1}{\rho _{14}} + \frac{1}{\rho _{24}}; \end{aligned}$$

this implies $$(I-C_{41}-C_{42})S_4F_{LM}(1,2,4) = 0$$.

Hence, the only term that we need to consider in Eq. (3.8) is the collinear subtraction

3.13 $$\begin{aligned} \big \langle (C_{41} + C_{42} ) F_{LM}(1,2,4) \big \rangle . \end{aligned}$$

We will consider the action of the operator $C_{41}$ on $F_{LM}(1,2,4)$ and then infer the result for the operator $C_{42}$. First, we find the collinear limit

3.14 $$\begin{aligned} C_{41} F_{LM}(1,2,4) = \frac{g_{s,b}^2}{E_4^2 \rho _{41}} (1-z) P_{qq}(z) \frac{F_{LM}( z \cdot 1, 2 )}{z}. \end{aligned}$$

We note that a new variable $z = 1 - E_4/E_1$ is introduced in Eq. (3.14). The notation $F_{LM}( z \cdot 1, 2 )$ implies that in the computation of $F_{LM}(1,2)$, cf. Eq. (3.10), the momentum $p_1$ is replaced with $z p_1$ everywhere, including the energy-momentum conserving $\delta$-function. We also used $P_{qq}(z)$ to denote the splitting function

3.15 $$\begin{aligned} P_{qq}(z) = C_\mathrm{F} \left[ \frac{1+z^2}{1-z} - \epsilon (1-z) \right] = P_{qq}^{(0)}(z) + \epsilon P_{qq}^{(\epsilon )}(z). \end{aligned}$$

To simplify $\langle C_{41} F_{LM}(1,2,4) \rangle$, we integrate over the emission angle of the gluon $g_4$, rewrite the integration over its energy as an integral over *z* and express $g_{s,b}$ in terms of the renormalized coupling $\alpha _\mathrm{s}(\mu )$. After straightforward manipulations we find

3.16 $$\begin{aligned} \langle C_{41} F_{LM}(1,2,4) \rangle= & {} -\frac{[\alpha _\mathrm{s}]}{\epsilon } \frac{\Gamma ^2(1-\epsilon )}{\Gamma (1-2\epsilon )}(2 E_1)^{-2 \epsilon }\nonumber \\&\times \int \limits _{z_\mathrm{min}}^{1} \frac{\mathrm{d} z }{(1-z)^{2\epsilon }} P_{qq}(z) \frac{F_{LM}( z \cdot 1, 2 )}{z}, \nonumber \\ \end{aligned}$$

where $z_\mathrm{min}= 1-E_\mathrm{max}/E_1$ and we introduced the short-hand notation

3.17 $$\begin{aligned}{}[\alpha _\mathrm{s}]\equiv \frac{\alpha _\mathrm{s}(\mu )}{2\pi }\frac{\mu ^{2\epsilon } e^{\epsilon \gamma _\mathrm{E}}}{\Gamma (1-\epsilon )}. \end{aligned}$$

We note that in Eq. (3.16) integration over *z* leads to divergences caused by the soft $z \rightarrow 1$ singularity in the splitting functions. These singularities are regulated dimensionally in Eq. (3.16). On the other hand, this equation has the form of a convolution of a hard matrix element with a splitting function, so we expect that divergences present there will cancel against the collinear subtraction terms. However, collinear subtractions employ regularization of soft singularities that is based on the plus-prescription. Our goal, therefore, is to rewrite Eq. (3.16) in such a way that all soft singularities are regulated by the plus-prescription; once this is done, combining this contribution with virtual corrections and collinear subtractions becomes straightforward.

To simplify the notation, we denote $F_{LM}( z \cdot 1, 2 )/z = G(z)$ and split $P_{qq}(z)$ into a piece that is singular at $z = 1$ and a regular piece

3.18 $$\begin{aligned} P_{qq}(z) = \frac{2C_\mathrm{F}}{(1-z)} + P^\mathrm{reg}_{qq}(z). \end{aligned}$$

We also note that we can extend the integral over *z* in Eq. (3.16) to $z = 0$ since if $E_4 > E_\mathrm{max}$, $F_{LM}(z \cdot 1,2)$ will vanish because there is not enough energy to produce the final state. We will use this fact frequently in our NNLO analysis. We write

3.19 $$\begin{aligned}&\int \limits _{z_\mathrm{min}}^{1} \frac{\mathrm{d} z }{(1-z)^{2\epsilon }} P_{qq}(z) \frac{F_{LM}( z \cdot 1, 2 )}{z} \nonumber \\&\quad = \int \limits _{0}^{1} \mathrm{d} z\; \left[ \frac{2 C_\mathrm{F} }{(1-z)^{1+2\epsilon } }+ (1-z)^{-2\epsilon }\; P^\mathrm{reg}_{qq}(z) \right] G(z) \nonumber \\&\quad = -\frac{C_\mathrm{F}}{\epsilon } G(1) + \int \limits _{0}^{1} \mathrm{d} z\; \left[ \frac{2 C_\mathrm{F} }{(1-z)^{1+2\epsilon } } \left( G(z) - G(1) \right) \right. \nonumber \\&\left. \qquad +\, (1-z)^{-2\epsilon }\; P^\mathrm{reg}_{qq}(z) G(z) \right] . \end{aligned}$$

The expression in Eq. (3.19) can be expanded in a power series in $\epsilon$ to the required order and the plus-distributions can be used to write the result in a compact form. Indeed, the following equation holds:

3.20 $$\begin{aligned} \frac{ G(z) - G(1) }{(1-z)^{1+2\epsilon } } = \left[ \sum \limits _{n=0}^{\infty } \frac{(-1)^n (2\epsilon )^n}{n!} \mathcal{D}_n(z) \right] \; G(z), \end{aligned}$$

where $\mathcal{D}_n(z) = [\ln ^n(1-z)/(1-z)]_+$. It is now straightforward to rewrite Eq. (3.16) in such a way that all soft, $z\rightarrow 1$, singularities are regulated using the plus-prescription. We use the fact that we are in the center-of-mass frame of the incoming $q\bar{q}$ pair, so that $2 E_1 = 2 E_2 = \sqrt{s}$. We find

3.21 $$\begin{aligned} \langle C_{41} F_{LM}(1,2,4) \rangle&= -\frac{[\alpha _\mathrm{s}] s^{- \epsilon } }{\epsilon } \frac{\Gamma ^2(1-\epsilon )}{\Gamma (1-2\epsilon )} \nonumber \\&\quad \times \left[ - \left( \frac{C_\mathrm{F}}{\epsilon } + \frac{3 C_\mathrm{F} }{2} \right) \bigl <F_{LM}(1,2) \bigr >\right. \nonumber \\&\quad \left. + \int \limits _{0}^{1} \mathrm{d} z \mathcal P_{qq,R}(z) \left\langle \frac{F_{LM}( z \cdot 1, 2 )}{z}\right\rangle \right] . \end{aligned}$$

The splitting function in Eq. (3.21) reads^5^

3.22 $$\begin{aligned} \mathcal P_{qq,R}(z) = {\hat{P}}_{qq}^{(0)}(z) + \epsilon \mathcal P^{(\epsilon )}_{qq,R}(z) + \mathcal O(\epsilon ^2), \end{aligned}$$

where ${\hat{P}}_{qq}^{(0)}(z)$ is the LO Altarelli–Parisi splitting kernel, see Eq. (A.1), and $\mathcal P^{(\epsilon )}_{qq,R}$ is defined as

3.23 $$\begin{aligned} \mathcal P^{(\epsilon )}_{qq,R}(z) = C_\mathrm{F}\bigg [2 (1+z) \ln (1-z) - (1-z) - 4 \mathcal{D}_1(z)\bigg ].\quad \end{aligned}$$

The result for $\langle C_{42} F_{LM}(1,2,4) \rangle$ is obtained by a simple replacement $F_{LM}(z \cdot 1, 2) \rightarrow F_{LM}(1, z\cdot 2)$ in Eq. (3.21). Putting everything together, we find the following result for the real-emission cross section:

3.24 $$\begin{aligned} 2 s\cdot \mathrm{d}\sigma ^\mathrm{R}&= 2 [\alpha _\mathrm{s}] s^{-\epsilon } \left( \frac{C_\mathrm{F}}{\epsilon ^2} + \frac{3C_\mathrm{F}}{2\epsilon } \right) \frac{\Gamma ^2(1-\epsilon )}{\Gamma (1-2\epsilon )} \big \langle F_{LM}(1,2) \big \rangle \nonumber \\&\quad +\,\big \langle \hat{O}_\mathrm{NLO} F_{LM}(1,2,4) \big \rangle \nonumber \\&\quad -\,\frac{[\alpha _\mathrm{s}] s^{-\epsilon } }{\epsilon } \frac{\Gamma ^2(1-\epsilon )}{\Gamma (1-2\epsilon )} \int \limits _{0}^{1} \mathrm{d} z \mathcal P_{qq,R}(z) \nonumber \\&\quad \times \left\langle \frac{F_{LM}( z \cdot 1, 2 )}{z} + \frac{F_{LM}( 1, z \cdot 2 )}{z} \right\rangle . \end{aligned}$$

We note that in Eq. (3.24) all singularities of the real-emission contribution are explicit and a straightforward expansion in $\epsilon$ is, in principle, possible. However, such an expansion is inconvenient since it involves higher-order $\epsilon$ terms of lower-multiplicity amplitude. To avoid these contributions, it is useful to combine Eq. (3.24) with virtual corrections and collinear counterterms.

For the virtual corrections, all divergent parts can be separated using Catani’s representation of renormalized one-loop scattering amplitudes [70]. We obtain

3.25 $$\begin{aligned} 2 s \cdot \mathrm{d}\sigma ^\mathrm{V}&= \big \langle F_{LV}(1,2) \big \rangle \nonumber \\&= \int \mathrm{d}\mathrm{Lips}_V\; 2 \mathrm{Re} \left\{ \mathcal{M}(1,2) \mathcal{M}^*_{\mathrm{1-loop}}(1,2) \right\} \; \nonumber \\&\quad \quad \times \mathcal{F}_\mathrm{kin}(1,2,V)\nonumber \\&= -2[\alpha _\mathrm{s}]\cos (\epsilon \pi ) \left( \frac{C_\mathrm{F}}{\epsilon ^2}+\frac{3}{2}\frac{C_\mathrm{F}}{\epsilon }\right) s^{-\epsilon } \nonumber \\&\quad \quad \times \big \langle F_{LM}(1,2) \big \rangle + \big \langle F^\mathrm{fin}_{LV}(1,2)\big \rangle , \end{aligned}$$

where $F^\mathrm{fin}_{LV}(1,2)$ is free of singularities and $\mu$-independent.

To arrive at the final result, we add virtual, real and collinear subtraction terms, cf. Eq. (2.9), and obtain

3.26 $$\begin{aligned}&2 s\cdot \mathrm{d}\hat{\sigma }^\mathrm{NLO} = \bigg . [\alpha _\mathrm{s}]\big \langle \mathcal S(1,2)F_{LM}(1,2)\big \rangle + \langle \hat{O}_\mathrm{NLO} F_{LM}(1,2,4) \rangle \nonumber \\&\quad + \left\langle F^\mathrm{fin}_{LV}(1,2)\right\rangle -\frac{[\alpha _\mathrm{s}] s^{-\epsilon }}{\epsilon } \frac{\Gamma ^2(1-\epsilon )}{\Gamma (1-2\epsilon )}\nonumber \\&\quad \times \int \limits _{0}^{1} \mathrm{d} z \mathcal P_{qq,R}(z) \left\langle \frac{F_{LM}( z \cdot 1, 2 )}{z} + \frac{F_{LM}( 1, z \cdot 2 )}{z} \right\rangle \nonumber \\&\quad + \frac{\alpha _\mathrm{s}(\mu )}{2\pi \epsilon } \int \limits _{0}^{1} \mathrm{d} z \; \hat{P}_{qq}^{(0)}(z) \left\langle \frac{F_{LM}( z \cdot 1, 2 )}{z} + \frac{F_{LM}( 1, z \cdot 2 )}{z} \right\rangle , \end{aligned}$$

where

3.27 $$\begin{aligned} \mathcal S(1,2) = 2 s^{-\epsilon }\left( \frac{C_\mathrm{F}}{\epsilon ^2}+\frac{3}{2}\frac{C_\mathrm{F}}{\epsilon }\right) \left[ \frac{\Gamma ^2(1-\epsilon )}{\Gamma (1-2\epsilon )}-\cos (\pi \epsilon )\right] , \end{aligned}$$

and the extra *z* terms in the denominator of the last line of Eq. (3.26) appear because of the *z*-dependent flux factor in the collinear counterterm cross section. Taking the limit $\epsilon \rightarrow 0$ in Eq. (3.26), we find the final formula for the NLO QCD contribution to the scattering cross section for $q(p_1) + \bar{q}(p_2) \rightarrow V +X$. It reads

3.28 $$\begin{aligned} 2 s\cdot \mathrm{d} \hat{\sigma }^\mathrm{NLO}&= \left\langle F^\mathrm{fin}_{LV}(1,2) + \frac{\alpha _\mathrm{s}(\mu )}{2\pi }\left[ \frac{2}{3}\pi ^2 C_\mathrm{F}F_{LM}(1,2)\right] \right\rangle \nonumber \\&\quad + \big \langle \hat{O}_\mathrm{NLO} F_{LM}(1,2,4) \big \rangle \nonumber \\&\quad +\frac{\alpha _\mathrm{s}(\mu )}{2\pi }\int \limits _{0}^{1} \mathrm{d} z \left[ \ln \left( \frac{s}{\mu ^2}\right) \hat{P}_{qq}^{(0)}(z) - \mathcal P^{(\epsilon )}_{qq,R}(z) \right] \nonumber \\&\quad \times \left\langle \frac{F_{LM}( z \cdot 1, 2 )}{z} + \frac{F_{LM}( 1, z \cdot 2 )}{z} \right\rangle . \end{aligned}$$

It follows that the NLO cross section is computed as a sum of low-multiplicity terms, including those where $F_{LM}(z \cdot 1, 2)$ or $F_{LM}(1,z \cdot 2)$ are convoluted with particular splitting functions, and the subtracted real-emission term described by $\langle \mathcal{O}_\mathrm{NLO} F_{LM}(1,2,4) \rangle$. We note that terms that involve matrix elements of different multiplicities, as well as terms that involve different types of convolutions, are separately finite. We will use this observation at NNLO, to check for the cancellation of $1/\epsilon$ divergences in an efficient way.

## The NNLO computation: general considerations

We would like to extend the above framework to NNLO in QCD. Apart from the UV and collinear renormalization discussed in Sect. 2, the NNLO cross section receives contributions from two-loop virtual corrections to $q \bar{q} \rightarrow V$ (double virtual), from one-loop corrections to the process with an additional gluon in the final state $q \bar{q} \rightarrow V+g$ (real-virtual), and from the tree-level process with two additional gluons in the final state $q \bar{q} \rightarrow V+gg$ (double real).

The double-virtual corrections can be dealt with in a straightforward way since all the divergences of the two-loop matrix elements are explicit, universal and well understood [70]. For our purposes, we only need to write them in a convenient form. The real-virtual corrections are more tricky, but do not require new conceptual developments. Indeed, the kinematic regions that lead to singularities in one-loop amplitudes with an additional gluon in the final state are identical to those appearing in the NLO computations and, furthermore, the limiting behavior of one-loop amplitudes with one additional parton is well understood [71–73]. Hence, we can deal with the real-virtual contribution by a simple generalization of what we did at NLO.

The qualitatively new element of the NNLO computation is the double-real emission process $q \bar{q} \rightarrow V+gg$. The methods that are applicable at next-to-leading order need to be adjusted to become useful in the NNLO case. However, somewhat surprisingly, these adjustments appear to be *relatively minor* although, of course, the bookkeeping becomes much more complex.

We begin by discussing the ultraviolet and PDF renormalizations at NNLO, as well as the double-virtual and the real-virtual contributions. We then move on to a more involved analysis of the double-real emission contribution to $\mathrm{d} \hat{\sigma }^\mathrm{NNLO}$.

## The NNLO computation: ultraviolet and PDF renormalization

In this section we study the contributions to $\mathrm{d} \hat{\sigma }^\mathrm{NNLO}$ coming from the ultraviolet and the collinear renormalization, beginning with the former. As mentioned previously, because the process $q \bar{q} \rightarrow V$ is driven by a conserved current which is independent of $\alpha _\mathrm{s}$, the ultraviolet renormalization contribution is very simple. Combining Eq. (2.10) and the first two lines of Eq. (3.26), it is straightforward to obtain

5.1 $$\begin{aligned} 2s\cdot \mathrm{d}\sigma ^\mathrm{ren}&= -\frac{\beta _0}{\epsilon }\frac{\alpha _\mathrm{s}(\mu )}{2\pi }\bigg [\big \langle \hat{\mathcal O}_\mathrm{NLO}F_{LM}(1,2,4)\big \rangle + \big \langle F^\mathrm{fin}_{LV}(1,2)\big \rangle \bigg ]\nonumber \\&\quad -\frac{\beta _0}{\epsilon }\left( \frac{\alpha _\mathrm{s}(\mu )}{2\pi }\right) ^2\frac{\Gamma (1-\epsilon )e^{\epsilon \gamma _\mathrm{E}}}{\Gamma (1-2\epsilon )} \left( \frac{\mu ^2}{s}\right) ^{\epsilon } \nonumber \\&\quad \times \Bigg \{ C_\mathrm{F}\left[ \frac{2}{\epsilon ^2}+\frac{3}{\epsilon }\right] \times \left[ 1 -\cos (\pi \epsilon )\frac{\Gamma (1-2\epsilon )}{\Gamma ^2(1-\epsilon )}\right] \nonumber \\&\quad \times \big \langle F_{LM}(1,2)\big \rangle -\frac{1}{\epsilon }\int _0^1 \mathrm{d}z \;\mathcal P_{qq,R}(z)\nonumber \\&\quad \times \left\langle \frac{F_{LM}(z\cdot 1,2)}{z}+\frac{F_{LM}(1,z\cdot 2)}{z}\right\rangle \Bigg \}. \end{aligned}$$

We proceed to the collinear subtraction. Rewriting Eq. (2.10) to make the convolutions explicit, we obtain

5.2 $$\begin{aligned} 2s\cdot \mathrm{d}\sigma ^\mathrm{CV}&= 2s\cdot \left( \frac{\alpha _\mathrm{s}(\mu )}{2\pi }\right) \frac{1}{\epsilon }\left( \hat{P}_{qq}^{(0)} \otimes \big [\mathrm{d}\sigma ^\mathrm{R}+\mathrm{d}\sigma ^\mathrm{V}\big ]\right. \nonumber \\&\quad \left. +\,\big [\mathrm{d}\sigma ^\mathrm{R}+\mathrm{d}\sigma ^\mathrm{V}\big ] \otimes \hat{P}_{qq}^{(0)} \right) \nonumber \\&\quad +\left( \frac{\alpha _\mathrm{s}(\mu )}{2\pi }\right) ^2\frac{1}{\epsilon ^2} \int \limits _0^1 \mathrm{d}z \; \mathrm{d}\bar{z} \; \hat{P}^{(0)}_{qq}(z)\nonumber \\&\quad \times \left\langle \frac{F_{LM}(z\cdot 1,\bar{z}\cdot 2)}{z\bar{z}} \right\rangle \times \hat{P}^{(0)}_{qq}(\bar{z})+\left( \frac{\alpha _\mathrm{s}(\mu )}{2\pi }\right) ^2 \nonumber \\&\quad \times \int \limits _0^1 \mathrm{d}z \left[ \frac{\big [\hat{P}^{(0)}_{qq}\otimes \hat{P}^{(0)}_{qq}\big ](z) -\beta _0 \hat{P}^{(0)}_{qq}(z)}{2\epsilon ^2} + \frac{\hat{P}^{(1)}_{qq}(z)}{2\epsilon }\right] \nonumber \\&\quad \times \Bigg \langle \frac{F_{LM}(z\cdot 1,2)}{z}\Bigg \rangle +\left( \frac{\alpha _\mathrm{s}(\mu )}{2\pi }\right) ^2 \nonumber \\&\quad \times \int \limits _0^1 \mathrm{d}z \left[ \frac{\big [\hat{P}^{(0)}_{qq}\otimes \hat{P}^{(0)}_{qq}\big ](z) -\beta _0 \hat{P}^{(0)}_{qq}(z)}{2\epsilon ^2} + \frac{\hat{P}^{(1)}_{qq}(z)}{2\epsilon }\right] \nonumber \\&\quad \times \Bigg \langle \frac{F_{LM}(1,z\cdot 2)}{z}\Bigg \rangle . \end{aligned}$$

Terms that involve convolutions of the various splitting functions with $F_{LM}$ are, in principle, straightforward to deal with. These terms are fully regulated and can be expanded in powers of $\epsilon$ without further ado. In practice, we combine those terms with other contributions in order to cancel the singularities prior to integration over *z*.

It is less straightforward to rewrite $\hat{P}\otimes \mathrm{d}\sigma ^\mathrm{R+V}$ and $\mathrm{d}\sigma ^\mathrm{R+V} \otimes \hat{P}$ in a form convenient for combining them with other contributions to $\mathrm{d} \hat{\sigma }^\mathrm{NNLO}$. We focus on $\hat{P}\otimes \mathrm{d}\sigma ^\mathrm{R+V}$, and consider the effect of the convolution on the first two lines of Eq. (3.26).

First, we consider the term proportional to $\langle \mathcal S(1,2) F_{LM}(1,2) \rangle$ in Eq. (3.26). It receives contributions from the divergent part of virtual corrections and from the soft regularization of collinear subtraction terms. These terms scale differently with *z*. The virtual correction depends on $s^{-\epsilon }$ which becomes $(zs)^{-\epsilon }$ once the momentum $p_1$ is changed to $z p_1$. On the other hand, the soft remainders of the collinear subtracted terms scale as $E_i^{-2\epsilon }$, with $i = 1,2$. Hence, in the calculation of $\mathrm{d}\sigma ^\mathrm{R+V}(z \cdot 1, 2)$, the corresponding contribution scales with *z* either as $\sim z^{-2\epsilon }$ or as $\sim 1$. Therefore, we have to compute

5.3 $$\begin{aligned} \int \limits _{0}^{1} \mathrm{d}z\; \hat{P}^{(0)}_{qq}(z) \mathcal S(z\cdot 1,2) \times \bigg \langle \frac{F_{LM}(z\cdot 1,2)}{z}\bigg \rangle , \end{aligned}$$

where

5.4 $$\begin{aligned} \mathcal S(z\cdot 1,2)&= s^{-\epsilon } \left( \frac{C_\mathrm{F}}{\epsilon ^2}+\frac{3}{2}\frac{C_\mathrm{F}}{\epsilon }\right) \nonumber \\&\quad \times \left[ \frac{\Gamma ^2(1-\epsilon )}{\Gamma (1-2\epsilon )} \big [ z^{-2\epsilon }+1\big ] -2 \cos (\pi \epsilon )z^{-\epsilon } \right] \nonumber \\&=s^{-\epsilon }C_\mathrm{F}\bigg [ \frac{2}{3}\pi ^2 + \ln ^2 z + \left( \pi ^2-\frac{2}{3}\pi ^2\ln z \right. \nonumber \\&\quad \left. + \frac{3}{2}\ln ^2 z - \ln ^3 z - 4\zeta _3 \right) \epsilon \bigg ] + \mathcal O(\epsilon ^2). \end{aligned}$$

For future convenience, we rewrite Eq. (5.3) as

5.5 $$\begin{aligned}&\int \limits _{0}^{1} \mathrm{d}z\; \hat{P}^{(0)}_{qq}(z) \mathcal S(z\cdot 1,2) \times \bigg \langle \frac{F_{LM}(z\cdot 1,2)}{z}\bigg \rangle \nonumber \\&\quad =s^{-\epsilon } \int \limits _{0}^{1} \mathrm{d}z\; \mathcal P_{qq,\mathrm{NLO_{CV}}}(z) \bigg \langle \frac{F_{LM}(z\cdot 1,2)}{z} \bigg \rangle , \end{aligned}$$

where the splitting function $\mathcal P_{qq,\mathrm{NLO_{CV}}}(z)$ is defined in Eq. (A.6).

The other two terms that we need involve convolutions of splitting function $\mathcal P_{qq,R}$ with $F_{LM}(1,2)$, cf. Eq. (3.26). The first term can be written as a double convolution

5.6 $$\begin{aligned}&\int \limits _0^1 \mathrm{d}x \;\hat{P}^{(0)}_{qq}(x) x^{-2\epsilon } s^{-\epsilon } \int \limits _0^1 \mathrm{d}y \; \mathcal P_{qq,R}(y)\; \Bigg \langle \frac{F_{LM}(xy\cdot 1,2)}{xy} \Bigg \rangle \nonumber \\&\quad = s^{-\epsilon } \int \limits _0^{1} \mathrm{d}z \; \big [\mathcal P_{qq}\otimes \mathcal P_{qq}\big ]_\mathrm{NLO_{CV}}(z) \;\Bigg \langle \frac{F_{LM}(z \cdot 1,2)}{z}\Bigg \rangle , \end{aligned}$$

where the splitting function $\big [\mathcal P_{qq}\otimes \mathcal P_{qq}\big ]_\mathrm{NLO_{CV}}$ is defined in Eq. (A.7). The second term is the left–right convolution,

5.7 $$\begin{aligned} s^{-\epsilon } \int \limits _{0}^{1} \mathrm{d}x\; \hat{P}^{(0)}_{qq}(x) \int \limits _{0}^{1} \mathrm{d}y\; \mathcal P_{qq,R}(y) \; \Bigg \langle \frac{F_{LM}(x\cdot 1,y\cdot 2)}{xy} \Bigg \rangle . \end{aligned}$$

Combining all these terms we find

5.8 $$\begin{aligned}&2s\cdot \bigg [\hat{P}^{(0)}_{qq}\otimes \mathrm{d}\sigma _{qq}^\mathrm{R+V}+ \mathrm{d}\sigma ^\mathrm{R+V}\otimes \hat{P}^{(0)}_{qq}\bigg ] = -\frac{[\alpha _\mathrm{s}] s^{-\epsilon } }{\epsilon }\frac{\Gamma ^2(1-\epsilon )}{\Gamma (1-2\epsilon )} \nonumber \\&\quad \times \Bigg \{ \int \limits _0^1 \mathrm{d}z\; \mathrm{d}\bar{z}~ \mathcal P_{qq,R}(z) \Bigg \langle \frac{F_{LM}(z\cdot 1,\bar{z}\cdot 2) +F_{LM}(\bar{z}\cdot 1,z\cdot 2)}{z\bar{z}} \Bigg \rangle \hat{P}^{(0)}_{qq}(\bar{z}) \nonumber \\&\quad + \int \limits _0^1 \mathrm{d}z \big [\mathcal P_{qq}\otimes \mathcal P_{qq}\big ]_\mathrm{NLO_{CV}}(z) \Bigg \langle \frac{F_{LM}(z\cdot 1,2)+F_{LM}(1,z\cdot 2)}{z}\Bigg \rangle \Bigg \} \nonumber \\&\quad +[\alpha _\mathrm{s}] s^{-\epsilon } \int \limits _{0}^{1} \mathrm{d}z\; \mathcal P_{qq,\mathrm{NLO_{CV}}}(z) \Bigg \langle \frac{F_{LM}(z\cdot 1,2)+F_{LM}(1,z\cdot 2)}{z}\Bigg \rangle \nonumber \\&\quad +\int \limits _0^1 \mathrm{d}z~ \hat{P}^{(0)}_{qq}(z) \Bigg \langle \hat{\mathcal O}_\mathrm{NLO}\frac{F_{LM}(z\cdot 1,2,4)+F_{LM}(1,z\cdot 2,4)}{z}\Bigg \rangle \nonumber \\&\quad + \int \limits _{0}^{1} \mathrm{d}z \;\hat{P}^{(0)}_{qq}(z) \Bigg \langle \frac{F^\mathrm{fin}_{LV}(z\cdot 1,2)+F^\mathrm{fin}_{LV}(1,z\cdot 2)}{z}\Bigg \rangle . \end{aligned}$$

Each term that appears on the right hand side of Eq. (5.8) is regularized and can be expanded in powers of $\epsilon$ independently of the other terms in that equation.

## The NNLO computation: double-virtual corrections

The calculation of double-virtual corrections proceeds in the standard way. We start with the scattering amplitude for $q \bar{q} \rightarrow V$ and write it as an expansion in the renormalized strong coupling constant

6.1 $$\begin{aligned} \mathcal M&= \mathcal M_\mathrm{tree} + \frac{\alpha _\mathrm{s}(\mu )}{2\pi }\mathcal M_\mathrm{1-loop} \nonumber \\&\quad + \left[ \frac{\alpha _\mathrm{s}(\mu )}{2\pi }\right] ^2 \mathcal M_\mathrm{2-loop} + \mathcal O(\alpha _\mathrm{s}^3), \end{aligned}$$

where the dependence of the scattering amplitudes on the momenta of the external particles is suppressed. By analogy with what was done in Sect. 3, we write

6.2 $$\begin{aligned}&2s\cdot \mathrm{d} \sigma ^\mathrm{VV} \nonumber \\&\quad = \left[ \frac{\alpha _\mathrm{s}(\mu )}{2\pi }\right] ^2 \int \mathrm{dLips}_{12 \rightarrow V} \left[ 2 \mathrm{Re} \{\mathcal{M}_\mathrm{2-loop} \mathcal{M}_\mathrm{tree}^*\}\right. \nonumber \\&\qquad \left. +\, |\mathcal{M}_\mathrm{1-loop}|^2 \right] \mathcal F_\mathrm{kin}(1,2,V)+\frac{\beta _0}{\epsilon } \left[ \frac{\alpha _\mathrm{s}(\mu )}{2\pi }\right] ^2 \nonumber \\&\qquad \times \int \mathrm{dLips}_{12 \rightarrow V} 2 \mathrm{Re} \{\mathcal{M}_\mathrm{1-loop} \mathcal{M}_\mathrm{tree}^*\} \mathcal F_\mathrm{kin}(1,2,V)\nonumber \\&\quad \equiv \big \langle F_{LVV}(1,2) \big \rangle , \end{aligned}$$

where in the second line we removed the renormalization contribution that is already accounted for in Eq. (5.1).

$F_{LVV}$ can be directly expanded in a Laurent series in $\epsilon$ and integrated over the phase space of the final state particles since this integration does not introduce soft or collinear divergences. Before doing that, it is convenient to explicitly extract the $1/\epsilon$ poles from $\langle F_{LVV}\rangle$. Soft and collinear singularities of a generic scattering amplitude are given in Ref. [70]. Using these results, we rewrite $\langle F_{LVV}\rangle$ as

6.3 $$\begin{aligned} \big \langle F_{LVV}(1,2) \big \rangle&= \left[ \frac{\alpha _\mathrm{s}(\mu )}{2\pi }\right] ^2 \left( \frac{\mu ^2}{s}\right) ^{2\epsilon } \Bigg \{ \frac{e^{2\epsilon \gamma _\mathrm{E}}}{\Gamma ^2(1-\epsilon )}\cos ^2(\epsilon \pi )\nonumber \\&\quad \times C_\mathrm{F}^2\left[ \frac{2}{\epsilon ^4}+\frac{6}{\epsilon ^3}+\frac{9}{2\epsilon ^2} \right] \nonumber \\&\quad +\frac{e^{\epsilon \gamma _\mathrm{E}}}{\Gamma (1-\epsilon )}\cos (2\epsilon \pi ) \bigg [ \frac{C_\mathrm{F}^2}{\epsilon }\left( -\frac{3}{8}+\frac{\pi ^2}{2}-6\zeta _3\right) \nonumber \\&\quad + C_\mathrm{A}C_\mathrm{F}\times \left( -\frac{11}{12\epsilon ^3}-\frac{83}{18\epsilon ^2} +\frac{\pi ^2}{12\epsilon ^2} \right. \nonumber \\&\quad \left. -\frac{961}{216\epsilon }-\frac{11\pi ^2}{48\epsilon } +\frac{13\zeta _3}{2\epsilon }\right) \bigg ] \Bigg \} \big \langle F_{LM}(1,2) \big \rangle \nonumber \\&\quad +\left[ \frac{\alpha _\mathrm{s}(\mu )}{2\pi }\right] \bigg [ \frac{e^{\epsilon \gamma _\mathrm{E}}}{\Gamma (1-\epsilon )} \cos (\epsilon \pi )C_\mathrm{F}\left( \frac{\mu ^2}{s}\right) ^\epsilon \nonumber \\&\quad \times \left( \frac{2}{\epsilon ^2}+\frac{3}{\epsilon }\right) + \frac{\beta _0}{\epsilon }\bigg ] \big \langle F^\mathrm{fin}_{LV}(1,2) \big \rangle \nonumber \\&\quad + \big \langle F^\mathrm{fin}_{LV^2}(1,2) \big \rangle + \big \langle F^\mathrm{fin}_{LVV}(1,2) \big \rangle . \end{aligned}$$

The sum of the last two terms in Eq. (6.3) is a finite remainder of the $\mathcal{O}(\alpha _\mathrm{s}^2)$ contribution to the virtual corrections once its divergent part is written in a form suggested in Ref. [70]. More specifically, $F^\mathrm{fin}_{LV^2}$ is the finite remainder of the one-loop amplitude squared, while $F^\mathrm{fin}_{LVV}$ is the genuine two-loop finite remainder. Note that, contrary to $F^\mathrm{fin}_{LV}(1,2)$ and $F^\mathrm{fin}_{LV^2}$, $F^\mathrm{fin}_{LVV}$ is scale-dependent; the scale-dependent contribution reads

6.4 $$\begin{aligned} F^\mathrm{fin}_{LVV}(\mu ^2,s) - F^\mathrm{fin}_{LVV}(s,s) = \frac{44}{3} C_\mathrm{F}C_\mathrm{A}\log \left( \frac{s}{\mu ^2}\right) F_{LM}. \end{aligned}$$

As follows from Eq. (6.3), the singularities of the double-virtual corrections are proportional to the leading-order contribution $F_{LM}(1,2)$ and to the finite part of the virtual corrections to the NLO cross section $F_{LV}^\mathrm{fin}(1,2)$, given in Sect. 3. Our goal is to rewrite the real-virtual and the double-real emission contributions in a way that allows explicit cancellation of the divergences in Eq. (6.3) without specifying hard matrix elements.

## The NNLO computation: real-virtual corrections

The kinematics of the real-virtual corrections is identical to the NLO case described in Sect. 3. The procedure for making these corrections expandable in $\epsilon$ is, therefore, the same. We write

7.1 $$\begin{aligned} 2s\cdot \mathrm{d}\sigma ^\mathrm{RV}&\equiv \big \langle F_{LRV}(1,2,4)\big \rangle = \big \langle S_4 F_{LRV}(1,2,4)\big \rangle \nonumber \\&\quad + \left\langle \big (I-S_4 \big )\big (C_{41}+C_{42}\big ) F_{LRV}(1,2,4) \right\rangle \nonumber \\&\quad + \big \langle \mathcal{O}_{NLO} F_{LRV}(1,2,4) \big \rangle . \end{aligned}$$

We remind the reader that, according to our notation, soft- and collinear-projection operators in Eq. (7.1) do not act on the phase space of the gluon $g_4$. It remains to compute the corresponding limits in Eq. (7.1) and to rewrite them, where appropriate, as convolutions of the hard matrix elements with splitting functions.

The soft limit of a general one-loop amplitude is discussed in Refs. [71, 72]. Adapting those results to our case, we find

7.2 $$\begin{aligned} E_4^2 S_4 F_{LRV}(1,2,4)&= 2C_\mathrm{F}g_{s,b}^2 \bigg [ \frac{\rho _{12}}{\rho _{14}\rho _{24}} F_{LV}(1,2) \nonumber \\&\quad -C_\mathrm{A}[\alpha _\mathrm{s}]\frac{1}{\epsilon ^2}\frac{\Gamma ^5(1-\epsilon )\Gamma ^3(1+\epsilon )}{\Gamma ^2(1-2\epsilon )\Gamma (1+2\epsilon )}\nonumber \\&\quad \times \left( \frac{\rho _{12}}{\rho _{14}\rho _{24}}\right) ^{1+\epsilon } E_4^{-2\epsilon } 2^{-\epsilon }F_{LM}(1,2)\bigg ]. \end{aligned}$$

We need to integrate Eq. (7.2) over the phase space of the gluon $g_4$. This can easily be done, with the result

7.3 $$\begin{aligned}&\big \langle S_4 F_{LRV}(1,2,4)\big \rangle = 2C_\mathrm{F}[\alpha _\mathrm{s}]\bigg [ \frac{\left( 4E_\mathrm{max}^2\right) ^{-\epsilon }}{\epsilon ^2} \big \langle F_{LV}(1,2)\big \rangle \nonumber \\&\quad -C_\mathrm{A}\frac{[\alpha _\mathrm{s}]}{\epsilon ^4} \frac{\Gamma ^5(1-\epsilon )\Gamma ^3(1+\epsilon )}{\Gamma ^2(1-2\epsilon )\Gamma (1+2\epsilon )} \frac{\left( 4E_\mathrm{max}^2\right) ^{-2\epsilon }}{4} \big \langle F_{LM}(1,2)\big \rangle \bigg ]. \end{aligned}$$

Note that in order to obtain a meaningful result, it is crucial that the integration over gluon energy is bounded from above; as we already explained in the context of the NLO computations, we use a parameter $E_\mathrm{max}$ for this purpose, cf. Eq. (3.3).

The second term that we need to consider is the soft-regulated collinear subtraction term

7.4 $$\begin{aligned} \left\langle \big (I-S_4\big ) \big ( C_{41} + C_{42}\big ) F_{LRV}(1,2,4)\right\rangle . \end{aligned}$$

We will only discuss the collinear projection operator $C_{41}$; the contribution of $C_{42}$ is obtained along the same lines. Collinear limits of loop amplitudes were studied in Refs. [71, 73]. Using these results and adapting them to our case, we find

7.5 $$\begin{aligned} C_{41} F_{LRV}(1,2,4)&= \frac{g_{s,b}^2}{E_4^2\rho _{41}} \Bigg [ (1-z)P_{qq}(z) \frac{F_{LV}(z\cdot 1,2)}{z} \nonumber \\&\quad +[\alpha _\mathrm{s}]\frac{\Gamma ^3(1-\epsilon )\Gamma (1+\epsilon )}{\Gamma (1-2\epsilon )} \nonumber \\&\quad \times \frac{ 2^{-\epsilon } E_1^{-2\epsilon }}{\rho _{41}^{\epsilon }} P_{qq}^\mathrm{loop,i}(z) \frac{F_{LM}(z\cdot 1,2)}{z}\Bigg ], \end{aligned}$$

where the splitting function $P_{qq}^\mathrm{loop,i}$ is given in Eq. (A.8). The soft-collinear limit is easily obtained by taking the collinear approximation in Eq. (7.2). We find

7.6 $$\begin{aligned}&S_4 C_{41} F_{LRV}(1,2,4) \nonumber \\&\quad = \frac{g_{s,b}^2}{E_4^2\rho _{41}}\Bigg [ 2C_\mathrm{F}F_{LV}(1,2)-2C_\mathrm{A}C_\mathrm{F}\frac{[\alpha _\mathrm{s}]}{\epsilon ^2}2^{-\epsilon }\nonumber \\&\quad \quad \times \frac{\Gamma ^5(1-\epsilon )\Gamma ^3(1+\epsilon )}{\Gamma ^2(1-2\epsilon )\Gamma (1+2\epsilon )} E_4^{-2\epsilon }\rho _{41}^{-\epsilon }F_{LM}(1,2)\Bigg ]. \end{aligned}$$

Integrating over emission angles of the gluon $g_4$ and rewriting the result through plus-distributions, following the discussion in Sect. 3, we obtain a convenient representation for the soft-regulated collinear subtraction term. It reads

7.7 $$\begin{aligned}&\left\langle \big (I-S_4\big )C_{41} F_{LRV}(1,2,4)\right\rangle \nonumber \\&\quad = \frac{[\alpha _\mathrm{s}] s^{-\epsilon }}{\epsilon }\frac{\Gamma ^2(1-\epsilon )}{\Gamma (1-2\epsilon )}\int \limits _{0}^{1} \mathrm{d}z\;\mathcal P_{qq,RV,1}(z) \nonumber \\&\quad \quad \times \left\langle \frac{F_{LV}(z\cdot 1,2)}{z}\right\rangle +\;\frac{[\alpha _\mathrm{s}]^2s^{-2\epsilon } }{\epsilon } \frac{\Gamma ^4(1-\epsilon )\Gamma (1+\epsilon )}{\Gamma (1-3\epsilon )}\nonumber \\&\quad \quad \times \int \limits _{0}^{1} \mathrm{d}z\; \mathcal P_{qq,RV,2}(z) \left\langle \frac{F_{LM}(z\cdot 1,2)}{z}\right\rangle , \end{aligned}$$

where the two splitting functions are defined in Eqs. (A.10) and (A.11). We replace $F_{LM}(z\cdot 1,2)$ with $F_{LM}(1,z\cdot 2)$ in Eq. (7.7) to obtain the result for $\left\langle \big (I-S_4\big )C_{42} F_{LRV}(1,2,4)\right\rangle$.

We are now in a position to present the final result for the real-virtual part. Collecting results shown in Eqs. (7.3) and (7.7), we obtain

7.8 $$\begin{aligned}&\big \langle F_{LV}(1,2,4)\big \rangle \nonumber \\&\quad =\big \langle \mathcal O_\mathrm{NLO} F_{LV}(1,2,4) \big \rangle +2C_\mathrm{F}[\alpha _\mathrm{s}] s^{-\epsilon } \Bigg [ \frac{1}{\epsilon ^2} \left( \frac{4E_\mathrm{max}^2}{s}\right) ^{-\epsilon } \nonumber \\&\quad \times \big \langle F_{LV}(1,2)\big \rangle -C_\mathrm{A}\frac{[\alpha _\mathrm{s}] s^{-\epsilon }}{4\epsilon ^4} \frac{\Gamma ^5(1-\epsilon )\Gamma ^3(1+\epsilon )}{\Gamma ^2(1-2\epsilon )\Gamma (1+2\epsilon )} \nonumber \\&\quad \times \left( \frac{4E_\mathrm{max}^2}{s}\right) ^{-2\epsilon }\big \langle F_{LM}(1,2)\big \rangle \Bigg ] +\frac{[\alpha _\mathrm{s}] s^{-\epsilon } }{\epsilon }\frac{\Gamma ^2(1-\epsilon )}{\Gamma (1-2\epsilon )}\nonumber \\&\quad \times \int \limits _{0}^{1} \mathrm{d}z~ \mathcal P_{qq,RV_1}(z) \bigg \langle \frac{F_{LV}(z\cdot 1,2)+F_{LV}(1,z\cdot 2)}{z}\bigg \rangle \nonumber \\&\quad +\frac{[\alpha _\mathrm{s}]^2 s^{-2\epsilon }}{\epsilon } \frac{\Gamma ^4(1-\epsilon )\Gamma (1+\epsilon )}{\Gamma (1-3\epsilon )}\nonumber \\&\quad \times \int \limits _0^1 \mathrm{d}z\; \mathcal P_{qq,RV_2}(z) \bigg \langle \frac{F_{LM}(z\cdot 1,2)+F_{LM}(1,z\cdot 2)}{z} \bigg \rangle . \end{aligned}$$

We stress that each term on the r.h.s. in Eq. (7.8) can be expanded in powers of $\epsilon$; we will make full use of this to cancel $1/\epsilon$ singularities when combining Eq. (7.8) with other contributions to $\mathrm{d}\hat{\sigma }^\mathrm{NNLO}$. To this end, it is useful to make all the $1/\epsilon$ singularities explicit in Eq. (7.8) by writing $F_{LV}(1,2)$ in terms of $F^\mathrm{fin}_{LV}(1,2)$ and $F_{LM}(1,2)$, cf. Eq. (3.25), and $F_{LV}(1,2,4)$ as

7.9 $$\begin{aligned} F_{LV}(1,2,4)&= [\alpha _\mathrm{s}]\Bigg [\left( \frac{1}{\epsilon ^2}+\frac{3}{2\epsilon }\right) \cos (\epsilon \pi ) (C_\mathrm{A}-2C_\mathrm{F})s_{12}^{-\epsilon }\nonumber \\&\quad -\left( \frac{C_\mathrm{A}}{\epsilon ^2}+\frac{3C_\mathrm{A}}{4\epsilon }\right) (s_{14}^{-\epsilon }+s_{24}^{-\epsilon })\Bigg ]\nonumber \\&\quad \times F_{LM}(1,2,4) +F^\mathrm{fin}_{LV}(1,2,4). \end{aligned}$$

We used $s_{ij} = 2p_i\cdot p_j$ and denoted a finite remainder which does not depend on the scale $\mu$ by $F^\mathrm{fin}_{LV}(1,2,4)$.

## The NNLO computation: double-real emission

### General considerations

In this section, we discuss the double-real emission contributions to $\mathrm{d}\hat{\sigma }^\mathrm{NNLO}$. Similar to the NLO case, we need to determine all kinematic configurations that may lead to singularities and understand the factorization of the matrix element that describes $q\bar{q} \rightarrow V+ gg$ in these regions. In the case of the two-gluon emission in $q \bar{q}$ annihilation into a colorless final state, the singular regions correspond to soft and/or collinear emissions, with collinear directions being the collision axis and the direction of either one of the two gluons.

The difficulty of the NNLO case is that each of these kinematic limits can be approached in several different ways and all of them have to be identified and regularized separately. To do so, we introduce several soft and collinear projection operators. They are defined as follows. Consider a quantity *A* that depends on the four-momenta of some or all of the particles in the process. The action of operators $SS, S_{4,5}, CC_{1,2}, C_{14},C_{15},C_{24},C_{25},C_{45}$ on *A* is described by the following formulas:

8.1 $$\begin{aligned} SSA&= \lim _{E_4,E_5\rightarrow 0} A, \mathrm{~ at~ fixed~ }E_5/E_4,\quad S_i A = \lim _{E_i \rightarrow 0} A,\nonumber \\ CC_i A&= \lim _{\rho _{4i},\rho _{5i}\rightarrow 0} A,\,\,\mathrm{~with~non~vanishing~}\nonumber \\&\quad \rho _{4i}/\rho _{5i},\rho _{45}/\rho _{4i},\rho _{45}/\rho _{5i},\quad C_{ij} A = \lim _{\rho _{ij}\rightarrow 0} A. \end{aligned}$$

To make use of these projection operators, we need to rewrite the two-gluon phase space in a way that allows these limits to be taken. It is convenient to order gluon energies as the first step. We write

8.2 $$\begin{aligned}&2s\cdot \mathrm{d}\sigma ^\mathrm{RR}=\int \frac{1}{2!} [dg_4][dg_5] |\mathcal{M}(1,2,4,5,V)|^2\nonumber \\&\qquad \times \mathrm{d}\mathrm{Lips}_{12-45\rightarrow V} \mathcal F_\mathrm{kin}(1,2,4,5,V)\nonumber \\&\quad = \int [dg_4][dg_5] \theta (E_4-E_5)|\mathcal{M}(1,2,4,5,V)|^2\nonumber \\&\qquad \times \mathrm{d}\mathrm{Lips}_{12-45\rightarrow V} \mathcal F_\mathrm{kin}(1,2,4,5,V) \nonumber \\&\quad = \int [dg_4][dg_5] \theta (E_4-E_5)F_{LM}(1,2,4,5) \nonumber \\&\quad = \big \langle F_{LM}(1,2,4,5) \big \rangle , \end{aligned}$$

where, as in Sect. 3, $\mathrm{d}\mathrm{Lips}$ is the phase space for the final state *V*, including momentum-conserving delta-function. The gluon phase space elements $[\mathrm{d} g_{4,5}]$ are defined as in Eq. (3.3)

8.3 $$\begin{aligned}{}[\mathrm{d}g] = \frac{d^{d-1}p_g}{(2\pi )^{d-1}2E_g} \theta (E_\mathrm{max}-E_g). \end{aligned}$$

As we already saw when considering the real-virtual contribution, it is necessary to introduce the $\theta$-function in order to define integrals over gluon energies in the soft limits. We work in the center-of-mass frame of the colliding quark and antiquark; it is in this frame that all the energies in the above formulas are defined.

We recall that, similar to the NLO case, soft and collinear projection operators act on everything that appears to the right of them. However, in the NNLO case we will find it convenient, occasionally, to also simplify the phase space in the collinear limits. If so, we will explicitly show the corresponding part of the phase space to the right of the operator. For example

8.4 $$\begin{aligned}&\big \langle \mathcal O [\mathrm{d}g_{4}] F_{LM}(1,2,4,5) \big \rangle \nonumber \\&\quad \equiv \int _{E_4>E_5} [\mathrm{d}g_{5}] \mathcal O [\mathrm{d}g_{4}] F_{LM}(1,2,4,5), \end{aligned}$$

implies that the operator $\mathcal{O}$ acts on $F_{LM}(1,2,4,5)$ and on the phase space element $[\mathrm{d}g_{4}]$.

We begin by extracting soft singularities from the double-real process, largely repeating what we have done at next-to-leading order.^6^ We write

8.5 $$\begin{aligned}&\big \langle F_{LM}(1,2,4,5) \big \rangle \nonumber \\&\quad = \big \langle SSF_{LM}(1,2,4,5) \big \rangle + \big \langle ( I - SS) F_{LM}(1,2,4,5) \big \rangle \nonumber \\&\quad = \big \langle SSF_{LM}(1,2,4,5) \big \rangle + \big \langle S_5 ( I - SS) F_{LM}(1,2,4,5) \big \rangle \nonumber \\&\quad \quad + \big \langle (I - S_5) ( I - SS) F_{LM}(1,2,4,5) \big \rangle . \end{aligned}$$

In Eq. (8.5), the last term is soft-regulated, in the second term gluon $g_5$ is soft and soft singularities associated with $g_4$ are regulated, and in the first term *both* $g_4$ and $g_5$ are soft.

All of these terms contain collinear singularities. Regulating these is more difficult since collinear singularities overlap. For this reason, we first need to split the phase space into mutually exclusive partitions that, ideally, select a single kinematic configuration that leads to singularities. We write

8.6 $$\begin{aligned} 1 = \sum _i w^i, \end{aligned}$$

where the label *i* runs through the elements of the set $i \in \{14,15;~ 24,25;~ 14,25;~ 15,24\}$. We refer to the first two elements of the set as triple-collinear and to the last two elements of the set as double-collinear partitions. We construct the weights $w^i$ in such a way that when they multiply the matrix element $\mathcal{M}(1,2,4,5)$ squared, the resulting expression is only singular in a well-defined subset of limits. For example, in the partition 14, 15 collinear singularities in $w^{14,15}|\mathcal{M}|^2$ only occur when gluons 4 and/or 5 are emitted along the direction of the incoming quark $q(p_1)$ or when their momenta are parallel to each other. Similarly, in the partition 24, 25, the singularities occur when momenta of $g_4$ and/or $g_5$ are parallel to $p_2$ or to each other. In the partitions 14, 25 and 15, 24, singularities only occur when momenta of $g_4$ and $g_5$ are collinear to $p_1$ and $p_2$ or $p_2$ and $p_1$, respectively. Apart from these requirements, the specific form of $w^i$ is arbitrary. Weights used in this calculation are given in Appendix B. In the following, we assume that weights do not depend on gluon energies and, therefore, commute with soft operators.

The triple-collinear partitions require further splitting to factorize all the relevant singularities. The purpose of this splitting is to establish a well-defined hierarchy for the parameters $\rho _{4i},\rho _{5i},\rho _{45}$, since different orderings correspond to different limiting behavior. This splitting is not unique; a possible choice consistent with the phase space parametrization that we employ later (cf. Appendix B) reads

8.7 $$\begin{aligned} 1&= \theta \left( \eta _{51}< \frac{\eta _{41}}{2}\right) + \theta \left( \frac{\eta _{41}}{2}< \eta _{51}< \eta _{41}\right) \nonumber \\&\quad + \theta \left( \eta _{41}< \frac{\eta _{51}}{2}\right) + \theta \left( \frac{\eta _{51}}{2}< \eta _{41} < \eta _{51}\right) \nonumber \\&\equiv \theta ^{(a)} + \theta ^{(b)} + \theta ^{(c)} + \theta ^{(d)}, \end{aligned}$$

where, as usual, $\eta _{ij} = \rho _{ij}/2 = (1-\cos \theta _{ij})/2$. We will refer to the four contributions shown in Eq. (8.7) as sectors *a*, *b*, *c* and *d*. We note that only two of the sectors are qualitatively different, since the other two are just obtained by the $4\leftrightarrow 5$ replacement. However, because of the energy ordering $E_5 < E_4$, we no longer have the $4\leftrightarrow 5$ symmetry, and we have to consider all the four sectors separately.

A suitable parametrization of all angular variables that supports splitting of the angular phase space as shown in Eq. (8.7) and allows for factorization of singularities in hard amplitudes was provided in Refs. [10, 11] and is reviewed in Appendix B. We will use this parametrization to carry out integrations over sectors $\theta ^{(a)},\theta ^{(b)},\theta ^{(c)},\theta ^{(d)}$ explicitly in what follows.

Having introduced partitions and sectors as a tool to identify singularities that may appear in the course of integrating over the angles of the final state gluons, we are now in a position to write the result for the double-real emission cross section as a sum of terms that either can be integrated in four dimensions, or that depend on hard matrix elements of lower multiplicity. The latter contributions still diverge, either explicitly or implicitly, and we will have to combine them with double-virtual and real-virtual contributions to arrive at the finite result.

We can thus rewrite the double-real emission cross section as

8.8 $$\begin{aligned}&\big \langle F_{LM}(1,2,4,5)\big \rangle \nonumber \\&\quad = \left\langle SSF_{LM}(1,2,4,5)\right\rangle +\left\langle \big [I-SS\big ] S_5 F_{LM}(1,2,4,5)\right\rangle \nonumber \\&\qquad + \big \langle F_{LM}^{s_rc_s}(1,2,4,5) \big \rangle + \big \langle F_{LM}^{s_rc_t}(1,2,4,5) \big \rangle \nonumber \\&\qquad + \big \langle F_{LM}^{s_rc_r}(1,2,4,5) \big \rangle , \end{aligned}$$

where the soft-regulated, single-collinear term $\langle F_{LM}^{s_rc_s} \rangle$ reads

8.9 $$\begin{aligned}&\langle F_{LM}^{s_rc_s} \rangle = \sum _{(ij)\in dc} \left\langle \big [I-SS\big ]\big [I-S_5\big ]\right. \nonumber \\&\quad \times \left. \bigg [ C_{4i} [\mathrm{d}g_{4}] + C_{5j}[\mathrm{d}g_{5}] \bigg ]w^{i4,j5}F_{LM}(1,2,4,5) \right\rangle \nonumber \\&\quad +\sum _{i\in tc} \bigg \langle \big [I-SS\big ]\big [I-S_5\big ] \bigg [ \theta ^{(a)} C_{5i} + \theta ^{(b)} C_{45}\nonumber \\&\quad + \theta ^{(c)} C_{4i} + \theta ^{(d)} C_{45} \bigg ][\mathrm{d}g_{4}] [\mathrm{d}g_{5}] w^{i4,i5}F_{LM}(1,2,4,5) \bigg \rangle , \end{aligned}$$

the soft-regulated, triple-collinear terms $\langle F_{LM}^{s_rc_t} \rangle$ reads

8.10 $$\begin{aligned}&\langle F_{LM}^{s_rc_t} \rangle = -\sum _{(ij)\in dc}\bigg \langle \big [I-SS\big ]\big [I-S_5\big ] \nonumber \\&\quad \times C_{4i}C_{5j}[\mathrm{d}g_{4}][\mathrm{d}g_{5}]w^{i4,j5}F_{LM}(1,2,4,5) \bigg \rangle \nonumber \\&\quad +\sum _{i\in tc} \bigg \langle \big [I-SS\big ]\big [I-S_5\big ] \bigg [ \theta ^{(a)} CC_i\big [I-C_{5i}\big ] \nonumber \\&\quad + \theta ^{(b)} CC_i\big [I-C_{45}\big ]+ \theta ^{(c)} CC_i\big [I-C_{4i}\big ]\nonumber \\&\quad + \theta ^{(d)} CC_i\big [I-C_{45}\big ] \bigg ]\nonumber \\&\quad \times [\mathrm{d}g_{4}] [\mathrm{d}g_{5}] w^{i4,i5}F_{LM}(1,2,4,5) \bigg \rangle , \end{aligned}$$

and the fully regulated term $\langle F_{LM}^{s_rc_r} \rangle$ reads

8.11 $$\begin{aligned}&\langle F_{LM}^{s_rc_r} \rangle = \sum _{(ij)\in dc}\bigg \langle \big [I-SS\big ]\big [I-S_5\big ] \bigg [(I- C_{5j})(I-C_{4i})\bigg ]\nonumber \\&\quad \times [\mathrm{d}g_{4}][\mathrm{d}g_{5}] w^{i4,j5}F_{LM}(1,2,4,5) \bigg \rangle \nonumber \\&\quad +\sum _{i\in tc} \bigg \langle \big [I-SS\big ]\big [I-S_5\big ] \bigg [ \theta ^{(a)} \big [I-CC_i\big ]\big [I-C_{5i}\big ]\nonumber \\&\quad + \theta ^{(b)}\big [I-CC_i\big ]\big [I-C_{45}\big ] \nonumber \\&\quad + \theta ^{(c)} \big [I-CC_i\big ]\big [I-C_{4i}\big ]+ \theta ^{(d)} \big [I-CC_i\big ]\big [I-C_{45}\big ] \bigg ]\nonumber \\&\quad \times [\mathrm{d}g_{4}] [\mathrm{d}g_{5}] w^{i4,i5}F_{LM}(1,2,4,5) \bigg \rangle . \end{aligned}$$

For the process under consideration, $dc=\{(1,2),(2,1)\}$ and $tc = \{1,2\}$. The above results are obtained by combining soft-regulated expression for $F_{LM}(1,2,4,5)$ with multiple partitions of unity for the angular projections, and the understanding of which collinear divergences can appear in each partition and sector. One can easily check, starting from Eq. (8.8), that the collinear projection operators add up to an identity operator for each partition and each sector.

For example, the contribution of the double-collinear sector *i*4, *j*5 follows from an expansion of an identity operator written in the following form:

8.12 $$\begin{aligned} I= & {} (I - C_{4i} + C_{4i}) ( I - C_{5j}+C_{5j}) \nonumber \\= & {} C_{4i} + C_{5j} - C_{4i}C_{5j}+(I - C_{4i}) (I - C_{5j} ). \end{aligned}$$

The reason we restrict ourselves to the subtraction of the $C_{4i}$ and $C_{5j}$ collinear projection operators is that in the partition *i*4, *j*5 no other collinear singularities appear, thanks to the damping factor $w^{i4,j5}$. Similarly, taking e.g. the sector *a* of the triple-collinear partition $w^{i4,i5}$, we write

8.13 $$\begin{aligned} I= & {} (I - CC_i + CC_i) ( I - C_{5i}+C_{5i}) = C_{5i} + CC_i(I-C_{5i})\nonumber \\&+ (I - CC_i) (I - C_{5i} ), \end{aligned}$$

because in this case a singularity can only occur either in a triple-collinear limit $\eta _{4i} \sim \eta _{5i} \rightarrow 0$ or if $\eta _{5i} \rightarrow 0$ at fixed $\eta _{4i}$.

It is worth pointing out a few things in connection with Eq. (8.8).

- The procedure that we used to write Eq. (8.8) is, in principle, process- and phase space parametrization-independent. We will use a particular parametrization of the phase space to perform the required computation but one should keep in mind that the freedom of changing the parametrization exists and, perhaps, it is worth exploring it in the future.
- The first term in Eq. (8.8), $\langle SSF_{LM}(1,2,4,5)\rangle$, is the double-soft subtraction term. It contains unregulated soft and collinear singularities and cannot be directly expanded in $\epsilon$. However, it only involves the tree-level matrix element $F_{LM}(1,2)$ which means that emitted gluons decouple from both the hard matrix element and the phase space constraints. When integrated over gluon energies and angles, this term gives rise to $1/\epsilon ^n$ poles, $n \le 4$.
- The second term in Eq. (8.8), $\left\langle \big [I-SS\big ] S_5 F_{LM}(1,2,4,5)\right\rangle$, is the double-soft regulated, single-soft subtraction term. It contains $F_{LM}(1,2,4)$ and matrix elements of *lower* multiplicity. This term still contains unregulated singularities that occur when the momentum of the gluon $g_4$ becomes collinear to the collision axis or to the direction of $g_5$. When integrated over gluon energies and angles, this term gives rise to $1/\epsilon ^n$ poles, with $n \le 3$.
- The term $\langle F_{LM}^{s_rc_s} \rangle$ in Eq. (8.9) is the soft-regulated single-collinear subtraction term. Note that thanks to the damping and $\theta$ factors only one kind of collinear singularity per term is present. $\langle F_{LM}^{s_rc_s} \rangle$ involves $F_{LM}(1,2,4(5))$, depending on the partition and the sector and, therefore, contains unregulated collinear singularities related to gluon emissions along the collision axis. It gives rise to $1/\epsilon ^2$ and $1/\epsilon$ poles.
- The term $\langle F_{LM}^{s_rc_t} \rangle$ in Eq. (8.10) is the triple-collinear subtraction, where all other singularities are regulated. As we will see, contributions of the double-collinear partitions to $\langle F_{LM}^{s_rc_t} \rangle$ have the “double-convolution” structure. The $\langle F_{LM}^{s_rc_t} \rangle$ term contains $1/\epsilon$ poles in contributions of triple-collinear partitions, and $1/\epsilon ^2$ poles in contributions of double-collinear partitions.
- The term $\langle F_{LM}^{s_rc_r} \rangle$ in Eq. (8.11) is completely regulated, thanks to the nested subtractions. It can be evaluated in four dimensions. It is the only term that involves the full hard matrix element for the process $q(p_1) + \bar{q}(p_2) \rightarrow V + g(p_4) + g(p_5)$.

Following our general strategy, we need to study the first four terms in Eq. (8.8), which involve matrix elements of reduced multiplicity, and rewrite them in terms of integrable quantities that admit straightforward expansions in the dimensional regularization parameter $\epsilon$. We will discuss how to do this in the following subsections.

**Table 1:** Coefficients of the $\epsilon$ expansion of the double-soft projected real-emission contribution. Full results are given in the first row. Results for individual color factors are given in the second and third rows. Numerical errors are such that their contribution to the final result is below the per mille level

$c^{SS}_{4}$	$c^{SS}_{3}$	$c^{SS}_{2}$	$c^{SS}_{1}$	$c^{SS}_{0}$
5.55554(2)	$$-11.73653(7)$$	$$-7.3253(7)$$	$$ -20.796(5)$$	$$-54.65(7)$$
$1.999995(8)~C_\mathrm{F}^2$	$-5.54530(5)~C_\mathrm{F}^2$	$1.1077(3)~C_\mathrm{F}^2$	$1.522(1)~C_\mathrm{F}^2$	$1.961(4)~C_\mathrm{F}^2$
$0.499999(2)~C_\mathrm{A}C_\mathrm{F}$	$-0.46960(1)~C_\mathrm{A}C_\mathrm{F}$	$-2.3236(1)~C_\mathrm{A}C_\mathrm{F}$	$-5.876(1)~C_\mathrm{A}C_\mathrm{F}$	$-14.52(1)~C_\mathrm{A}C_\mathrm{F}$

### The double-soft subtraction term

We begin with the discussion of the first term in Eq. (8.8), $\left\langle SSF_{LM}(1,2,4,5)\right\rangle$. It corresponds to the kinematic situation where momenta of both gluons vanish at a comparable rate. The corresponding limit for the amplitude squared is given in Refs. [74, 75] and allows us to write

8.14 $$\begin{aligned}&\left\langle SSF_{LM}(1,2,4,5)\right\rangle \nonumber \\&\quad = g_{s,b}^2\int _{E_4 > E_5} [\mathrm{d}g_{4}] [\mathrm{d}g_{5}] \; \mathrm{Eik}(1,2,4,5) \; \big \langle F_{LM}(1,2 )\big \rangle , \end{aligned}$$

where

8.15 $$\begin{aligned} \mathrm{Eik}(1,2,4,5)&= 4 C_\mathrm{F}^2 S_{12}(4) S_{12}(5) +C_\mathrm{A}C_\mathrm{F}\big [ 2 S_{12}(4,5)\nonumber \\&\quad -S_{11}(4,5)-S_{22}(4,5)\big ], \end{aligned}$$

and [75]

8.16 $$\begin{aligned} S_{ij}(q)&\equiv \frac{p_i\cdot p_j}{(p_i\cdot q)(p_j\cdot q)} = 2\frac{s_{ij}}{s_{iq}s_{jq}},\nonumber \\ S_{ij}(q_1,q_2)&= S_{ij}^{so}(q_1,q_2) + \frac{p_i\cdot q_1 p_j\cdot q_2+p_i\cdot q_2 p_j\cdot q_1}{p_i\cdot q_{12} p_j\cdot q_{12}}\nonumber \\&\quad \times \,\left[ \frac{(1-\epsilon )}{(q_1\cdot q_2)^2}-\frac{1}{2} S_{ij}^{so}(q_1,q_2)\right] \nonumber \\&\quad -\,2\frac{p_i\cdot p_j}{q_1\cdot q_2 p_i\cdot q_{12} p_j \cdot q_{12}}, \end{aligned}$$

with

8.17 $$\begin{aligned} S_{ij}^{so}(q_1,q_2)= & {} \frac{p_i\cdot p_j}{q_1\cdot q_2} \left( \frac{1}{p_i\cdot q_1 p_j\cdot q_2} + \frac{1}{p_i\cdot q_2 p_j\cdot q_1}\right) \nonumber \\&-\frac{(p_i\cdot p_j)^2}{p_i\cdot q_1 p_j\cdot q_1 p_i\cdot q_2 p_j\cdot q_2}. \end{aligned}$$

At this point, we stress again that the hard matrix element $F_{LM}(1,2)$ corresponds to a tree-level process and that the emitted gluons have no impact on the kinematic properties of the final state *V* because they decouple from the energy-momentum conserving $\delta$-function.

The goal now is to integrate the eikonal factor over the momenta of the two gluons. We note that, at this point, unless put in by hand, the integration over gluon energies becomes unconstrained since the energy-momentum conserving $\delta$-function becomes independent of the gluon momenta after the double-soft limit is taken. It is for this reason that we need to introduce $E_\mathrm{max}$ as in Eq. (8.3).

To satisfy constraints on gluon energies, we parametrize them as

8.18 $$\begin{aligned} E_4 = E_\mathrm{max} ~x_1,\quad E_5 = E_4 x_2 = E_\mathrm{max}\; x_1 x_2. \end{aligned}$$

Written in these variables, the eikonal factor becomes

8.19 $$\begin{aligned} \mathrm{Eik}(1,2,4,5) = E_\mathrm{max}^{-4} x_1^{-4} x_2^{-2} \mathcal{E} (x_2, n_1, n_2, n_4, n_5 ). \end{aligned}$$

The important point is that the dependence on the overall energy scale $x_1$ factorizes and that the remaining (complicated) function $\mathcal{E}$ is independent of energies of the incoming partons. We also use the parametrization of energies Eq. (8.18) in the phase space to obtain

8.20 $$\begin{aligned}&\int [\mathrm{d}g_{4}][\mathrm{d}g_{5}] \mathrm{Eik}(1,2,4,5) = -\frac{E_{\mathrm {max}}^{-4\epsilon }}{4\epsilon }\nonumber \\&\quad \times \int \limits _{0}^{1}\frac{\mathrm{d}x_2}{x_2^{1+2\epsilon }} \frac{\mathrm{d} \Omega _4}{2 (2\pi )^{d-1}} \frac{\mathrm{d} \Omega _5}{2 (2\pi )^{d-1}} \mathcal{E} (x_2, n_1, n_2, n_4, n_5 ). \nonumber \\ \end{aligned}$$

For the case of a color-singlet final state, this integral is just a constant.^7^ The abelian contribution is simple to obtain since it is just the product of NLO structures. In principle it should be possible to compute the non-abelian contribution analytically along the lines of e.g. Refs. [76–78]. However, it is also straightforward to obtain it numerically. To do this, we partition the phase space as in residue-improved sector decomposition [10, 11]. The corresponding formulas for the angular phase space are given in Appendix B. Performing the required decomposition and integrating Eq. (8.20) numerically, we obtain the $\epsilon$-expansion of the double-soft subtraction term,

8.21 $$\begin{aligned} \bigl < SSF_{LM}(1,2,4,5) \bigr >= & {} [\alpha _\mathrm{s}]^2 \big \langle E_{\mathrm {max}}^{-4\epsilon } F_{LM}(1,2)\big \rangle \nonumber \\&\times \left( \frac{c^{SS}_{4}}{\epsilon ^4} +\frac{c^{SS}_{3}}{\epsilon ^3}+\frac{c^{SS}_{2}}{\epsilon ^2} +\frac{c^{SS}_{1}}{\epsilon }+c^{SS}_{0} \right) , \nonumber \\ \end{aligned}$$

where $c^{SS}_i = C_\mathrm{F}^2\cdot c^{SS}_{i,C_\mathrm{F}^2} + C_\mathrm{A}C_\mathrm{F}\cdot c^{SS}_{i,C_\mathrm{A}C_\mathrm{F}}$. Numerical values of the coefficients $c^{SS}$ are shown in Table 1. There we also report numerical results for the abelian contribution, which are in perfect agreement with the analytic calculation. The result for the double-soft subtraction $\bigl < SSF_{LM}(1,2,4,5) \bigr >$ does not require any further regularization; we will later combine it with other contributions with tree-level kinematics to cancel the $1/\epsilon$ singularities explicitly.

### The single-soft term

We now consider the second term that contributes to Eq. (8.8). It is a double-soft regulated, single-soft singular expression that reads

8.22 $$\begin{aligned} \left\langle \big [I-SS\big ] S_5 F_{LM}(1,2,4,5)\right\rangle . \end{aligned}$$

Note that this contribution implicitly depends on $F_{LM}(1,2,4)$ and $F_{LM}(1,2)$, and the hard matrix element that appears in $F_{LM}(1,2,4)$ still contains collinear singularities that arise when the momentum of gluon $g_4$ becomes parallel to the momenta of the incoming partons or to the direction of $g_5$. These divergences will have to be extracted and regulated.

We start by computing the soft limit for the gluon $g_5$. We find

8.23 $$\begin{aligned}&S_{5} F_{LM}(1,2,4,5)\nonumber \\&\quad =\frac{g^{2}_{s,b}}{E^{2}_{5}}\left[ (2C_{F}-C_{A})\frac{\rho _{12}}{\rho _{15}\rho _{45}}+C_{A}\left( \frac{\rho _{14}}{\rho _{15}\rho _{45}}+\frac{\rho _{24}}{\rho _{25}\rho _{45}}\right) \right] \nonumber \\&\qquad \times F_{LM}(1,2,4). \end{aligned}$$

Since the gluon $g_5$ decouples from the hard matrix element, we can integrate over its momentum. We find

8.24 $$\begin{aligned} \langle ( I - SS) S_5F_{LM}(1,2,4,5) \rangle = \langle J_{124}\; ( I - S_4) F_{LM}(1,2,4) \rangle , \end{aligned}$$

where

8.25 $$\begin{aligned} J_{124}&= \frac{ [\alpha _\mathrm{s}] E_4^{-2\epsilon }}{\epsilon ^2} \bigg [ (2C_\mathrm{F}-C_\mathrm{A})(2\rho _{12})^{-\epsilon }K_{12}\nonumber \\&\quad + C_\mathrm{A}\big [(2\rho _{14})^{-\epsilon } K_{14} + (2\rho _{24})^{-\epsilon } K_{24}\big ] \bigg ], \end{aligned}$$

and

8.26 $$\begin{aligned} K_{ij}= & {} \frac{\Gamma ^2(1-\epsilon )}{\Gamma (1-2\epsilon )} \eta _{ij}^{1+\epsilon }F_{21}(1,1,1-\epsilon ,1-\eta _{ij})\nonumber \\= & {} 1 + \left[ \text {Li}_2(1-\eta _{ij}) - \frac{\pi ^2}{6}\right] \epsilon ^2 +\mathcal O(\epsilon ^3). \end{aligned}$$

We need to simplify Eq. (8.24) because it still contains collinear singularities that appear when the momentum of gluon $g_4$ becomes parallel to the collision axis. To extract them, we write

8.27 $$\begin{aligned}&\big \langle J_{124}\big [I-S_4\big ]F_{LM}(1,2,4) \big \rangle = \big \langle \big [I- C_{41} - C_{42}\big ]\nonumber \\&\quad \times J_{124}\big [I-S_4\big ]F_{LM}(1,2,4) \big \rangle \nonumber \\&\quad + \big \langle \big [ C_{41} + C_{42}\big ]J_{124}\big [I-S_4\big ]F_{LM}(1,2,4) \big \rangle . \end{aligned}$$

We reiterate that according to our notational conventions, the collinear projection operators do not act on the phase space element of the gluon $g_4$ in Eq. (8.27). The first term in Eq. (8.27) is explicitly regulated and can be expanded in powers of $\epsilon$; for this reason, we will only be concerned with the second term. We focus on the projection operator $C_{41}$; the contribution of the projection operator $C_{42}$ is then obtained by analogy.

First, we consider how $C_{41}$ acts on $J_{124}$. Using $\eta _{12}=1$, $C_{41}\rho _{24}=\rho _{12}$ and taking the $\rho _{41}\rightarrow 0$ limit on $K_{14}$, $K_{24}$ we obtain

8.28 $$\begin{aligned} C_{41} J_{124}&= \frac{[\alpha _\mathrm{s}]}{\epsilon ^2}E_4^{-2\epsilon } \frac{\Gamma ^2(1-\epsilon )}{\Gamma (1-2\epsilon )}\nonumber \\&\quad \times \bigg [ 2^{1-2\epsilon }C_\mathrm{F}+ C_\mathrm{A}\Gamma (1+\epsilon )\Gamma (1-\epsilon )(2\rho _{14})^{-\epsilon } \bigg ]. \end{aligned}$$

Integrating over the energy and angle of the gluon $g_4$ we arrive at

8.29 $$\begin{aligned}&\big \langle C_{41}J_{124}\big [I-S_4\big ]F_{LM}(1,2,4) \big \rangle \nonumber \\&\quad = -\frac{[\alpha _\mathrm{s}]^2 s^{-2\epsilon }}{\epsilon ^3} \bigg [ 2C_\mathrm{F}\frac{\Gamma ^4(1-\epsilon )}{\Gamma ^2(1-2\epsilon )}+ C_\mathrm{A}\frac{\Gamma ^4(1-\epsilon )\Gamma (1+\epsilon )}{2 \Gamma (1-3\epsilon )} \bigg ]\nonumber \\&\qquad \times \int \limits _{z_\mathrm{min}}^1 \frac{\mathrm{d}z}{(1-z)^{1+4\epsilon }} \hat{\mathcal P}_{qq}^{(-)}\bigl <F_{LM}(z\cdot 1,2)\bigr >, \end{aligned}$$

where $z_\mathrm{min}= 1-E_\mathrm{max}/E_4$. The splitting function operator $\hat{\mathcal P}_{qq}^{(-)}$ is defined by means of the following equation:

8.30 $$\begin{aligned} \hat{\mathcal P}_{qq}^{(-)}f(z) = \mathcal{P}_{qq}(z) f(z) - 2 C_\mathrm{F} f(1), \end{aligned}$$

where

8.31 $$\begin{aligned} \mathcal{P}_{qq}(z) = (1-z) \frac{P_{qq}(z)}{z}, \end{aligned}$$

and the splitting function $P_{qq}(z)$ is given in Eq. (3.15).

Note that $\big \langle C_{41} J_{124}\big [I-S_4\big ]F_{LM}(1,2,4) \big \rangle$ in Eq. (8.29) can be directly expanded in powers of $\epsilon$ since all the singularities are regulated. The only problem that needs to be addressed is the fact that the integration over *z* does not start at $z=0$, as is the case for the convolutions. The lower integration boundary $z_\mathrm{min}$ must be kept in Eq. (8.29) because of the subtraction term $2 C_\mathrm{F} F_{LM}(1,2)$. Indeed, if $z_\mathrm{min}$ is replaced with zero, the integration over the gluon energy for this term extends to the region $E_4 > E_\mathrm{max}$, in contradiction with the original phase space parametrization. The extension of the integration region in Eq. (8.29) is accomplished following steps discussed in the context of the NLO QCD computation in Sect. 3. Effectively, this leads to a redefinition of the splitting function

8.32 $$\begin{aligned}&\int \limits _{z_\mathrm{min}}^{1} \frac{\mathrm{d} z }{(1-z)^{1+4\epsilon }} \hat{\mathcal P}_{qq}^{(-)}F_{LM}(z\cdot 1,2) \nonumber \\&\quad \equiv \int \limits _{0}^{1} \mathrm{d} z \mathcal{P}_{qq,RR_1}(z) \frac{F_{LM}(z \cdot 1, 2)}{z}, \end{aligned}$$

where $\mathcal{P}_{qq,RR_1}(z)$ is given in Eq. (A.12). We note that the contribution of the collinear operator $C_{42}$ to the second term in Eq. (8.27) is computed in a similar way; the computation leads to the same result as in Eq. (8.29) up to an obvious replacement $F_{LM}(z \cdot 1, 2) \rightarrow F_{LM}(1, z \cdot 2)$.

Putting everything together, we find the final result for the double-soft regulated single-soft singular contribution to $\langle F_{LM}(1,2,4,5) \rangle$,

8.33 $$\begin{aligned}&\big \langle \big [I-SS\big ] S_5 F_{LM}(1,2,4,5)\big \rangle \nonumber \\&\quad = \big \langle \big [I- C_{41} - C_{42}\big ]\big [I-S_4\big ] J_{124} F_{LM}(1,2,4) \big \rangle \bigg . \nonumber \\&\quad \quad -\frac{[\alpha _\mathrm{s}]^2 s^{-2\epsilon }}{\epsilon ^3} \left[ 2C_\mathrm{F}\frac{\Gamma ^4(1-\epsilon )}{\Gamma ^2(1-2\epsilon )} +\frac{C_\mathrm{A}}{2}\frac{\Gamma ^4(1-\epsilon )\Gamma (1+\epsilon )}{\Gamma (1-3\epsilon )} \right] \nonumber \\&\quad \quad \times \int \limits _0^1 \mathrm{d}z\;\mathcal P_{qq,RR_1}(z) \Bigg \langle \frac{F_{LM}(z\cdot 1,2) + F_{LM}(1,z\cdot 2) }{z} \Bigg \rangle . \end{aligned}$$

### The single-collinear term

Next, we consider the soft-regulated, single-collinear contribution to $\langle F_{LM}(1,2,4,5) \rangle$

8.34 $$\begin{aligned}&\langle F_{LM}^{s_rc_s} \rangle = \sum _{(ij)\in dc} \left\langle \big [I-SS\big ]\big [I-S_5\big ] \right. \nonumber \\&\quad \times \left. \bigg [ C_{4i} [\mathrm{d}g_{4}] + C_{5j}[\mathrm{d}g_{5}] \bigg ]w^{i4,j5}F_{LM}(1,2,4,5) \right\rangle \nonumber \\&\quad +\sum _{i\in tc} \bigg \langle \big [I-SS\big ]\big [I-S_5\big ] \bigg [ \theta ^{(a)} C_{5i} + \theta ^{(b)} C_{45}+ \theta ^{(c)} C_{4i} \nonumber \\&\quad + \theta ^{(d)} C_{45} \bigg ] \times [\mathrm{d}g_{4}] [\mathrm{d}g_{5}] w^{i4,i5}F_{LM}(1,2,4,5) \bigg \rangle . \end{aligned}$$

We need to rewrite Eq. (8.34) in such a way that extraction of the remaining collinear singularities becomes straightforward. We note that $\langle F_{LM}^{s_rc_s} \rangle$ contains contributions from double- and triple-collinear partitions, which we will treat separately. We will start with the double-collinear partitions since they are somewhat simpler.

#### The double-collinear partitions

In this subsection, we will consider the contribution of the double-collinear partitions to $\langle F_{LM}^{s_rc_s} \rangle$. We begin with the partition 14, 25. For the first term, we need to compute

8.35 $$\begin{aligned}&\big [I-SS\big ] \big [I-S_5\big ]C_{41} w^{14,25}F_{LM}(1,2,4,5)\nonumber \\&\quad = \tilde{w}_{4||1}^{14,25}\big [I-SS\big ] \big [I-S_5\big ]C_{41}F_{LM}(1,2,4,5), \end{aligned}$$

where $\tilde{w}_{4||1}^{14,25} = \lim _{\rho _{41}\rightarrow 0} w^{14,25}$ does not depend on the momentum of gluon $g_4$ anymore. To further simplify Eq. (8.35), note that collinear and soft projection operators commute with each other and that

8.36 $$\begin{aligned}&SS(I-S_5) C_{41}F_{LM}(1,2,4,5) \sim SSF_{LM}(1-4,2,5)\nonumber \\&\quad - SSS_5 F_{LM}(1-4,2,5) = 0. \end{aligned}$$

This implies that we can drop the $SS$ term in Eq. (8.35). We use the collinear limit for $C_{41} F_{LM}(1,2,4,5)$ obtained by a straightforward generalization of Eq. (3.14). We define $z = 1-E_4/E_1$ and obtain

8.37 $$\begin{aligned}&C_{41}(I-S_5)F_{LM}(1,2,4,5) \nonumber \\&\quad = \frac{g_{s,b}^2}{E_4^2 \rho _{41}} \mathcal{P}_{qq}(z) \big [I-S_5\big ] F_{LM}(z\cdot 1,2,5). \end{aligned}$$

The function $\mathcal{P}_{qq}(z)$ was introduced in Eq. (8.31).

We now consider the phase space. According to Eq. (8.34), $C_{41}$ acts on the phase space element $[\mathrm{d}g_{4}]$. We introduce $x_3 = (1-\cos \theta _{41})/2$ to get

8.38 $$\begin{aligned}&g_{s,b}^2 [\mathrm{d}g_{4}] \theta (E_4-E_5) = [\alpha _\mathrm{s}] s^{-\epsilon } E_4^2 \rho _{14}\nonumber \\&\quad \times \frac{\mathrm{d}z}{(1-z)^{1+2\epsilon }} \big [x_3(1-x_3)\big ]^{-\epsilon }\frac{\mathrm{d}x_3}{x_3}\theta (z_\mathrm{max}(E_5)-z), \nonumber \\ \end{aligned}$$

with

8.39 $$\begin{aligned} z_\mathrm{max}(E_5) = 1- \frac{E_5}{E_1} = 1 - \frac{2E_5}{\sqrt{s}} . \end{aligned}$$

In this parametrization, the action of $C_{41}$ implies replacing $[ x_3 ( 1- x_3) ]^{-\epsilon }$ with $x_3^{-\epsilon }$. Putting everything together, we obtain

8.40 $$\begin{aligned}&\big \langle \big [I-SS\big ] \big [I-S_5\big ]C_{41} w^{14,25} [\mathrm{d}g_{4}] F_{LM}(1,2,4,5)\big \rangle \nonumber \\&\quad =-\frac{[\alpha _\mathrm{s}] s^{-\epsilon } }{\epsilon } \int \limits _{z_\mathrm{min}}^{z_\mathrm{max}(E_5)} \frac{\mathrm{d}z}{(1-z)^{1+2\epsilon } } \mathcal{P}_{qq}(z)\nonumber \\&\qquad \times \big \langle \tilde{w}_{4||1}^{14,25} \big [I-S_5\big ]F_{LM}(z\cdot 1,2,5)\big \rangle . \end{aligned}$$

Note that, similar to the NLO case, the lower boundary $z_\mathrm{min}$ is not important when integrating $F_{LM}(z\cdot 1,2,4)$ since for $z<z_\mathrm{min}$ there is no sufficient energy in the incoming partons to produce the required final state.

Next we consider the action of the $C_{52}$ projection operator. Following the preceding discussion and using $z = 1-E_5/E_2 = 1-2E_5/\sqrt{s}$, we obtain

8.41 $$\begin{aligned}&\left\langle \big [I-SS\big ] \big [I-S_5\big ]C_{52} w^{14,25} [\mathrm{d}g_{5}] F_{LM}(1,2,4,5)\right\rangle \nonumber \\&\quad =-\frac{[\alpha _\mathrm{s}] s^{-\epsilon } }{\epsilon } \int \limits _{z_\mathrm{min}(E_4)}^{1} \frac{\mathrm{d}z}{ ( 1-z)^{1+2\epsilon } } \hat{\mathcal P}_{qq}^{(-)}\big \langle \tilde{w}_{5||2}^{14,25}\nonumber \\&\quad \quad \times F_{LM}(1,2 \cdot z,4)\big \rangle . \end{aligned}$$

The operator $\hat{\mathcal P}_{qq}^{(-)}$ was introduced in Eq. (8.30) and

8.42 $$\begin{aligned} z_\mathrm{min}(E_4) = 1-\frac{E_4}{E_2}=1-\frac{2E_4}{\sqrt{s}}. \end{aligned}$$

The sum of Eqs. (8.40) and (8.41) gives the required result for the collinear sector 14, 25.

The partition 15, 24 is obtained from the results for 14, 25 after a few obvious replacements. We find

8.43 $$\begin{aligned}&\left\langle \big [I-SS\big ] \big [I-S_5\big ]C_{42} w^{15,24} [\mathrm{d}g_{4}] F_{LM}(1,2,4,5)\right\rangle \nonumber \\&\quad =-\frac{[\alpha _\mathrm{s}] s^{-\epsilon }}{\epsilon } \int \limits _{z_\mathrm{min}}^{z_\mathrm{max}(E_5)} \frac{\mathrm{d}z}{ ( 1-z)^{1+2\epsilon } } \mathcal{P}_{qq}(z)\nonumber \\&\qquad \times \big \langle \tilde{w}_{4||2}^{15,24} \big [I-S_5\big ]F_{LM}(1,z\cdot 2,5)\big \rangle \end{aligned}$$

and

8.44 $$\begin{aligned}&\left\langle \big [I-SS\big ] \big [I-S_5\big ]C_{51} w^{15,24} [\mathrm{d}g_{5}] F_{LM}(1,2,4,5)\right\rangle \nonumber \\&\quad =-\frac{[\alpha _\mathrm{s}] s^{-\epsilon }}{\epsilon } \int \limits _{z_\mathrm{min}(E_4)}^{1} \frac{\mathrm{d}z}{(1-z)^{1+2\epsilon }} \hat{\mathcal P}_{qq}^{(-)}\big \langle \tilde{w}_{5||1}^{15,24} \nonumber \\&\quad \quad \times F_{LM}(1\cdot z,2,4)\big \rangle . \end{aligned}$$

We can now combine the contributions of the two double-collinear partitions. In doing so, it is convenient to always denote the “resolved” (i.e. the non-collinear) gluon by $g_4$. Out of the four terms that we need to combine, two correspond to the collinear emission along the direction of the incoming quark $p_1$ and two along the direction of the incoming antiquark $p_2$. We consider terms that belong to the former category first.

When combining results, it is important to realize that $z_\mathrm{min}(E_4) = z_\mathrm{max}(E_4) = 1 - 2E_4/\sqrt{s}$. We will denote it by $z_4 > z_\mathrm{min} = 1-2 E_\mathrm{max}/\sqrt{s}$. After straightforward manipulations we find

8.45 $$\begin{aligned}&\left\langle \big [I-SS\big ]\big [I-S_5\big ] \left[ C_{41} w^{14,25} +C_{51} w^{15,24}\right] \right. \nonumber \\&\qquad \times \left. [\mathrm{d}g_{4}] [\mathrm{d}g_{5}] F_{LM}(1,2,4,5)\right\rangle \nonumber \\&\quad = -\frac{[\alpha _\mathrm{s}s^{-\epsilon }]}{\epsilon } \int \limits _0^1 \frac{\mathrm{d}z}{(1-z)^{1+2\epsilon }} \nonumber \\&\qquad \times \Bigg \langle \tilde{w}_{5||1}^{15,24} \bigg ( \hat{\mathcal P}_{qq}^{(-)}\big [I-S_4\big ]F_{LM}(z\cdot 1,2,4) \nonumber \\&\qquad + \theta \left( z_4 - z \right) 2C_\mathrm{F}\big [I-S_4\big ]F_{LM}(1,2,4)\nonumber \\&\qquad +\theta \left( z - z_4 \right) \hat{\mathcal P}_{qq}^{(-)}S_4 F_{LM}(z\cdot 1,2,4)\bigg )\Bigg \rangle . \end{aligned}$$

Note that the lower integration boundary in this formula should be $z = z_\mathrm{min}$ but we can extend the integration region to $z = 0$, without making a mistake. This is so because every time $F_{LM}(z\cdot 1,\ldots )$ appears in the integrand, the $z>z_{\mathrm{min}}$ condition is automatically enforced by the requirement that the initial state should have enough energy to produce the final state. On the other hand, if $F_{LM}(z\cdot 1,\ldots )$ does not appear, $\theta$-functions require that $z> z_4 > z_\mathrm{min}$. We also note that, thanks to explicit subtractions and constraints due to $\theta$-functions, each term in Eq. (8.45) vanishes if $z\rightarrow 1$ or $E_4\rightarrow 0$. Finally, we stress that $\hat{\mathcal P}_{qq}^{(-)}$ and $S_4$ commute since they act on different variables.

We can write a similar equation for the sum of the two terms where the collinear gluon is emitted along the direction of the antiquark $\bar{q}(p_2)$. Finally, putting everything together, we obtain the contribution of the double-collinear partitions to $\langle F_{LM}^{s_r c_s} \rangle$. We find

8.46 $$\begin{aligned} \bigg \langle&\big [I-SS\big ]\big [I-S_5\big ] \left[ (C_{41} + C_{52}) w^{14,25} + ( C_{51} + C_{42} ) w^{24,15} \right] \nonumber \\&\quad \times [\mathrm{d}g_{4}] [\mathrm{d}g_{5}] F_{LM}(1,2,4,5)\bigg \rangle \nonumber \\&\quad = -\frac{[\alpha _\mathrm{s}] s^{-\epsilon }}{\epsilon } \int \limits _0^1 \frac{\mathrm{d}z}{(1-z)^{1+2\epsilon }}\times \bigg \langle \tilde{w}_{5||1}^{15,24} \Bigg \{ \hat{\mathcal P}_{qq}^{(-)}\big [I-S_4\big ] \nonumber \\&\quad \big [ F_{LM}(z\cdot 1,2,4) + F_{LM}(1,z \cdot 2,4) \big ]+ \theta \left( z_4 - z \right) 4C_\mathrm{F}\nonumber \\&\quad \big [I-S_4\big ]F_{LM}(1,2,4) +\theta \left( z - z_4 \right) \hat{\mathcal P}_{qq}^{(-)}S_4 \big [ F_{LM}(z\cdot 1,2,4)\nonumber \\&\quad + F_{LM}(1,z\cdot 2,4) \big ] \bigg \}\Bigg \rangle . \end{aligned}$$

Note that the second term in the curly bracket only depends on *z* through the $\theta$-function and so the *z*-integration of this term can be performed explicitly.

#### The triple-collinear partition 14, 15

We consider the triple-collinear partition 14, 15 and study the contribution of single-collinear limits in Eq. (8.34). We begin with sector (*a*). The relevant expression reads

8.47 $$\begin{aligned} \left\langle \big [I-SS\big ]\big [I-S_5\big ]\theta ^{(a)} C_{51} [\mathrm{d}g_{5}] w^{14,15} F_{LM}(1,2,4,5)\right\rangle . \end{aligned}$$

The calculation is identical to the case of the double-collinear partition except that we need to account for the constraint that defines sector (*a*) when integrating over the angle of gluon $g_5$. Writing $\rho _{14} = 2x_{3}$ and $\rho _{15} = 2x_4$, and taking $\theta _a = \theta (\rho _{14}/2 - \rho _{15})$, we find

8.48 $$\begin{aligned} \int \limits _0^1\theta ^{(a)} \frac{\mathrm{d}x_4}{x_4^{1+\epsilon }} = \int \limits _0^{x_3/2} \frac{\mathrm{d}x_4}{x_4^{1+\epsilon }} = -\frac{(x_3/2)^{-\epsilon }}{\epsilon } = -\frac{(\rho _{14}/4)^{-\epsilon }}{\epsilon }. \end{aligned}$$

Using this result, we obtain

8.49 $$\begin{aligned}&\left\langle \big [I-SS\big ] \big [I-S_5\big ]\theta ^{(a)} C_{51} w^{14,15} [\mathrm{d}g_{5}] F_{LM}(1,2,4,5)\right\rangle \nonumber \\&\quad =-\frac{[\alpha _\mathrm{s}] s^{-\epsilon }}{\epsilon } \int \limits _{0}^{1} \frac{\mathrm{d}z}{(1-z)^{1+2\epsilon }}\times \left\langle \tilde{w}_{5||1}^{14,15} \left( \frac{\rho _{14}}{4}\right) ^{-\epsilon }\right. \nonumber \\&\qquad \times \left. \theta (z - z_4) \hat{\mathcal P}_{qq}^{(-)}F_{LM}(1,2,4)\right\rangle . \end{aligned}$$

A similar calculation for the sector (*c*) gives

8.50 $$\begin{aligned}&\left\langle \big [I-SS\big ] \big [I-S_5\big ]\theta ^{(c)} C_{41} w^{14,15} [\mathrm{d}g_{4}] F_{LM}(1,2,4,5)\right\rangle \nonumber \\&\quad = -\frac{[\alpha _\mathrm{s}]s^{-\epsilon }}{\epsilon } \times \int \limits _{0}^{1} \frac{\mathrm{d}z}{(1-z)^{1+2\epsilon } } \bigg \langle \tilde{w}_{4||1}^{14,15} \left( \frac{\rho _{15}}{4}\right) ^{-\epsilon } \theta (z_5 - z)\nonumber \\&\qquad \times \mathcal{P}_{qq}(z) \big [I-S_5\big ]F_{LM}(z\cdot 1,2,5)\bigg \rangle . \end{aligned}$$

In parallel to the case of the double-collinear partitions, it is again convenient to always call the resolved gluon $g_4$. We combine contributions of sectors (*a*) and (*c*), renaming $g_5 \rightarrow g_4$ where appropriate, and we obtain

8.51 $$\begin{aligned}&\left\langle \big [I-SS\big ] \big [I-S_5\big ]\left( \theta ^{(a)}C_{51}+\theta ^{(c)}C_{41}\right) \right. \nonumber \\&\qquad \times \left. w^{14,15} [\mathrm{d}g_{4}][\mathrm{d}g_{5}] F_{LM}(1,2,4,5)\right\rangle \nonumber \\&\quad =-\frac{[\alpha _\mathrm{s}] s^{-\epsilon } }{\epsilon } \int \limits _{0}^{1} \frac{\mathrm{d}z}{(1-z)^{1+2\epsilon }} \Bigg \langle \tilde{w}_{5||1}^{14,15}\left( \frac{\rho _{14}}{4}\right) ^{-\epsilon } \nonumber \\&\qquad \times \bigg \{ \left[ I- \theta (z_4 - z) S_4\right] \times \hat{\mathcal P}_{qq}^{(-)}F_{LM}(z\cdot 1,2,4)\nonumber \\&\qquad + \theta (z_4 - z) 2C_\mathrm{F}\big [I-S_4\big ] F_{LM}(1,2,4)\bigg \}\Bigg \rangle . \end{aligned}$$

We now turn to sectors (*b*) and (*d*). These sectors are different from the other triple-collinear sectors and from the double-collinear partitions. Indeed, the single-collinear limits that we consider in sectors (*b*) and (*d*) correspond to gluons $g_4$ and $g_5$ becoming collinear to each other. We consider

8.52 $$\begin{aligned} \left\langle \big [I-SS\big ] \big [I-S_5\big ]\theta ^{(b,d)} C_{45} w^{14,15} [\mathrm{d}g_{5}] F_{LM}(1,2,4,5)\right\rangle , \end{aligned}$$

and start with the discussion of how the collinear projection operator $C_{45}$ acts on $F_{LM}$. We find

8.53 $$\begin{aligned} C_{45}F_{LM}(1,2,4,5)&= \frac{g_{s,b}^2}{E_5^2\rho _{45}} \frac{E_5}{E_4} P_{gg,\mu \nu }(z)F_{LM}^{\mu \nu }(1,2,4+5)\nonumber \\&=\frac{g_{s,b}^2}{E_5^2\rho _{45}}\frac{z}{1-z}\bigg [ P_{gg}^{(0)}(z)F_{LM}(1,2,45) \nonumber \\&\quad + P_{gg}^\perp (z)\kappa _{\perp ,\mu }\kappa _{\perp ,\nu } F_{LM}^{\mu \nu }(1,2,45) \bigg ], \end{aligned}$$

where $p_{4+5} = p_{45} = (E_4+E_5)/E_4 \cdot p_4$, i.e. the hard matrix element must be taken in the collinear limit. The splitting functions are

8.54 $$\begin{aligned}&P_{gg}^{(0)}(z) = 2C_\mathrm{A}\left( \frac{z}{1-z}+\frac{1-z}{z}\right) ,\nonumber \\&P^\perp _{gg}(z) = 4C_\mathrm{A}(1-\epsilon )z(1-z), \end{aligned}$$

and *z* is the fraction of the total momentum $p_{45} = p_4 + p_5$ carried by gluon $g_5$,

8.55 $$\begin{aligned} z = E_5/( E_4 + E_5). \end{aligned}$$

The vector $\kappa _\perp$ is a normalized transverse vector

8.56 $$\begin{aligned} \kappa = k_\perp /\sqrt{-k_\perp ^2}, \end{aligned}$$

defined by the following decomposition:

8.57 $$\begin{aligned} p_5 = \alpha p_4 + \beta {\bar{p}}_4 + k_{\perp }, \end{aligned}$$

where $${\bar{p}}_4 = (p_4^{(0)}, - \vec p_4)$$ and $$k_{\perp }\cdot p_4 = k_{\perp }\cdot {\bar{p}}_4 = 0$$.

We now construct these vectors explicitly. For this, we need the parametrization of the angular phase space of the two gluons valid for sectors (*b*) and (*d*); it is given in Appendix B. Here we repeat the relevant formulas and discuss simplifications that occur in the limit where the momenta of $g_4$ and $g_5$ become collinear. We write the four-momenta of $g_4$ and $g_5$ as

8.58 $$\begin{aligned}&p_4^\mu = E_4 \big ( t^\mu + \cos \theta _{41} e_3^{\mu } + \sin \theta _{41} b^\mu \big ),\nonumber \\&p_5 \!=\! E_5 \big ( t^\mu + \cos \theta _{51} e_3^\mu + \sin \theta _{51} \left( \cos \varphi _{45} b^\mu \!+\! \sin \varphi _{45} a^{\mu } \right) \big ), \end{aligned}$$

where $t^{\mu } = (1,\vec {0})$, $e_3^\mu = (0,0,0,1;0\ldots )$, $b \cdot t = b \cdot e_3 = 0$ and $a \cdot t = a \cdot e_3 = a \cdot b = 0$. Our goal is to parametrize the phase space in such a way that explicit averaging over directions of $k_\perp$ can be performed. The phase space parametrization for sectors (*b*) and (*d*) can be written as

8.59 $$\begin{aligned}{}[\mathrm{d}g_{4}] [\mathrm{d}g_{5}]&= \left( E_4^{1-2\epsilon } \mathrm{d}E_4\right) \left( E_5^{1-2\epsilon } \mathrm{d}E_5 \right) \nonumber \\&\quad \times \theta (E_\mathrm{max}-E_4) \theta (E_4-E_5) \mathrm{d}\Omega ^{(b,d)}_{45}, \end{aligned}$$

with

8.60 $$\begin{aligned} C_{45}\left[ \frac{\mathrm{d}\Omega _{45}^{(b,d)}}{\eta _{45}}\right]&= N_{\epsilon }^{(b,d)} \frac{\mathrm{d}\Omega _{g_4}}{(2\pi )^{d-1}} \left[ \frac{1}{8\pi ^2} \frac{(4\pi )^\epsilon }{\Gamma (1-\epsilon )}\right] \nonumber \\&\quad \times \left[ \frac{\mathrm{d}\Omega _{d-3,a}}{\Omega _{d-3}}\right] x_3^{-\epsilon }(1-x_3)^{\epsilon }\frac{\mathrm{d}x_4}{x_4^{1+2\epsilon }}\mathrm{d}\Lambda , \end{aligned}$$

where

8.61 $$\begin{aligned}&\mathrm{d}\Lambda \equiv \frac{\Gamma (1+\epsilon )\Gamma (1-\epsilon )}{\Gamma (1+2\epsilon )\Gamma (1-2\epsilon )} \frac{\lambda ^{-1/2+\epsilon }(1-\lambda )^{-1/2-\epsilon }}{\pi } \mathrm{d}\lambda ,\nonumber \\&N_\epsilon ^{(b,d)} = \left[ \frac{\Gamma (1-\epsilon )\Gamma (1+2\epsilon )}{\Gamma (1+\epsilon )} \right] . \end{aligned}$$

Here, $x_4\rightarrow 0$ corresponds to the 4||5 collinear limit, $\eta _{45} = (1-\cos \theta _{45} )/2$, $x_3 = \rho _{41}/2$ and $\lambda$ is related to the azimuthal angle $\varphi _{45}$. Further details about the parametrization as well as expressions of scalar products in terms of $x_{3,4}$ and $\lambda$ can be found in Appendix B. In this parametrization, the vector $\kappa _\perp$ reads^8^

8.62 $$\begin{aligned} \kappa _\perp = a \sqrt{1-\lambda } + r \sqrt{\lambda },\quad r = \sin \theta _{41} \; e_3 - \cos \theta _{41} \; b. \quad \end{aligned}$$

Using this expression in Eq. (8.53) together with momenta parametrization Eq. (8.58) and the phase space limit Eq. (8.60), we observe that integrations over $\lambda$ and the directions of the vector $a^\mu$ can be performed since neither $\lambda$ nor $a^\mu$ appear in the hard matrix element. We define

8.63 $$\begin{aligned} \big \langle \kappa _{\perp }^\mu \kappa _{\perp }^\nu \big \rangle \equiv \int \mathrm{d}\Lambda \frac{\mathrm{d}\Omega _{d-3,a}}{\Omega _{d-3}} \kappa _{\perp }^\mu \kappa _{\perp }^\nu . \end{aligned}$$

Using

8.64 $$\begin{aligned} \int \frac{\mathrm{d}\Omega _{d-3,a}}{\Omega _{d-3}}\; a^\mu = 0,\quad \int \frac{\mathrm{d}\Omega _{d-3,a}}{\Omega _{d-3}}\; a^\mu a^\nu = -\frac{g_{\perp ,(d-3)_a}^{\mu \nu }}{d-3}, \end{aligned}$$

and

8.65 $$\begin{aligned} \int \mathrm{d}\Lambda = 1,\quad \int \lambda \mathrm{d}\Lambda = \frac{1+2\epsilon }{2} ,\quad \int (1-\lambda ) \mathrm{d}\Lambda = \frac{1-2\epsilon }{2}, \end{aligned}$$

we find

8.66 $$\begin{aligned} \big \langle \kappa _{\perp }^\mu \kappa _{\perp }^\nu \big \rangle&= -\frac{g^{\mu \nu }_{\perp ,(d-3)_a}}{2} + \frac{1+2\epsilon }{2} r^\mu r^\nu \nonumber \\&=\frac{1}{2}\bigg [ -g^{\mu \nu }_{\perp ,(d-3)_a}+r^\mu r^\nu \bigg ] +\epsilon r^\mu r^\nu \nonumber \\&=-\frac{g_{\perp ,(d-2)}}{2} + \epsilon r^\mu r^\nu . \end{aligned}$$

It follows that averaging over transverse directions introduces an $\epsilon$-dependent leftover, as a consequence of the chosen phase space parametrization [10, 11].

To write the result of the integration over unresolved phase space variables, it is convenient to define an additional splitting function,

8.67 $$\begin{aligned} P_{gg}(z,\epsilon )= & {} P_{gg}^{(0)}(z) + \frac{P_{gg}^\perp (z)}{2}\nonumber \\= & {} 2C_\mathrm{A}\left( \frac{1-z}{z} + \frac{z}{1-z} + z(1-z)(1-\epsilon )\right) .\nonumber \\ \end{aligned}$$

Combining the results discussed above, we write an expression for the contribution of the $C_{45}$ collinear projection operator in sector (*b*). We obtain

8.68 $$\begin{aligned}&\big \langle \big [I-SS\big ] \big [I-S_5\big ]\theta ^{(b)} C_{45} w^{14,15} [\mathrm{d}g_{5}] F_{LM}(1,2,4,5)\big \rangle \bigg .\nonumber \\&\quad =-\frac{[\alpha _\mathrm{s}]}{2\epsilon } N_\epsilon ^{(b)} \int _{E_4>E_5} [\mathrm{d}g_{4}] \tilde{w}_{4||5}^{14,15} x_3^{-\epsilon }(1-x_3)^{\epsilon }\nonumber \\&\qquad \times \frac{\mathrm{d}E_5}{E_{5}^{1+2\epsilon }} \big [I-SS\big ] \big [I-S_5\big ] \mathcal{P}_{45}(1,2,4,5), \end{aligned}$$

where

8.69 $$\begin{aligned} \mathcal P_{45}(1,2,4,5)&= \frac{E_5}{E_4} \Bigg [P_{gg}\left[ \frac{E_5}{E_4+E_5},\epsilon \right] F_{LM}(1,2,4+5) \nonumber \\&\quad + \epsilon P_{gg}^\perp \left[ \frac{E_5}{E_4+E_5} \right] r_\mu r_\nu F_{LM}^{\mu \nu }(1,2,4+5) \Bigg ]. \end{aligned}$$

It follows from Eq. (8.68) that we need to know how an operator $(I - SS)(I - S_5)$ acts on $\mathcal P_{45}(1,2,4,5)$. We recall that the action of $SS$ on energy variables implies that $E_4,E_5 \rightarrow 0$ at fixed $E_4/E_5$. Computing the soft limits is simple and standard except for the spin-correlated part that we address below. In principle, we need to know three soft limits $SS, S_5$ and $SSS_5$. However, since

8.70 $$\begin{aligned} \lim _{E_5 \rightarrow 0} P^\perp _{gg}(E_5/(E_4+E_5)) = 0, \end{aligned}$$

we only need to consider $SSr_\mu r_\nu F_{LM}^{\mu \nu }(1,2,4+5)$. We find it using the known soft limits for amplitudes and the explicit form of the vector *r* given in Eq. (8.62). Indeed, since $r \cdot p_4 = 0$ and $r^2 =-1$, $r^\mu$ is a valid polarization vector of the gluon with momentum $4+5$, in the collinear 4||5 approximation. For this reason, the soft limit of $r_\mu r_\nu F_{LM}^{\mu \nu }$ follows from the standard soft limit of the amplitude for $q \bar{q} \rightarrow V + g$, not averaged over gluon polarizations. We obtain

8.71 $$\begin{aligned} SSr_\mu r_\nu F_{LM}^{\mu \nu }(1,2,4)&= \frac{C_\mathrm{F}}{E_4^2} \left( \frac{n_2\cdot r}{\rho _{24}}- \frac{n_1\cdot r}{\rho _{14}}\right) ^2F_{LM}(1,2) \nonumber \\&=\frac{2C_\mathrm{F}}{E_4^2} \frac{2\sin ^2\theta _{14}}{\rho _{14}^2 \rho _{24}^2} F_{LM}(1,2)\nonumber \\&= \frac{2C_\mathrm{F}}{E_4^2} \frac{\rho _{12}}{\rho _{14} \rho _{24}} F_{LM}(1,2) \nonumber \\&= S_4 F_{LM}(1,2,4). \end{aligned}$$

Collecting all the soft limits, we find

8.72 $$\begin{aligned}&\big [I- SS\big ] \big [I- S_5\big ] \mathcal P_{45}(1,2,4,5)\nonumber \\&\quad =\frac{z}{1-z} \bigg [ -g_{\mu \nu } P_{gg}(z,\epsilon ) + \epsilon P^\perp _{gg}(z) r_\mu r_\nu \bigg ] \nonumber \\&\qquad \quad \times \big [I-S_{45}\big ]F_{LM}^{\mu \nu }(1,2,45) -2C_\mathrm{A}\big [I-S_4\big ]\nonumber \\&\quad \qquad \times F_{LM}(1,2,4), \end{aligned}$$

where *z* is defined in Eq. (8.55). This implies

8.73 $$\begin{aligned} E_5 = z E_{45},\quad E_4 = (1-z) E_{45},\quad E_{45} = E_4 + E_5. \end{aligned}$$

We can now use Eqs. (8.72) in (8.68) and integrate over all variables that are not present in the hard matrix elements. This requires different variable transformations in the first and the second terms in Eq. (8.72). To integrate the first term, we change the integration variables from $E_{4,5}$ to $E_{45}$ and *z*.

**Fig. 1:**
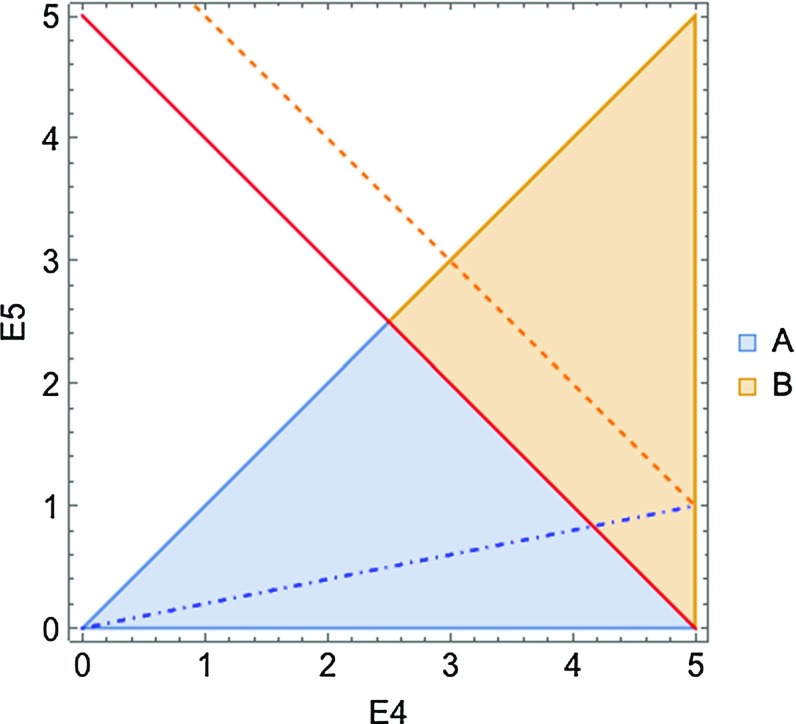
Integration region for the $(E_4,E_5)\rightarrow (E_{45},z)$ change of variables. The *colored triangle* is the allowed $0<E_5<E_4<E_\mathrm{max}$ region. The *blue region* “A” is the “physical” one, i.e. the one which is not removed by a phase space $\theta$-function inside $F_{LM}(1,2,45)$. The *orange region* “B” only contributes to the soft limit, since there no $\theta$-function from $F_{LM}$ is preventing it. Lines of fixed $E_{45}$ are shown in *solid red* (for $E_{45}=E_\mathrm{max}$) and *dashed orange* (for $E_{45}>E_\mathrm{max}$). In *dot-dashed blue*, lines of constant *z* are shown. In the “physical” region, only the $z<1/2$ condition is relevant. In the “B” region, we also have to impose $z>1-E_\mathrm{max}/E_{45}$, to prevent the $E_{45}$ integration to go *outside the triangle* (see the intersection of *blue* and *orange lines*)

We find that the integration region splits into two regions (cf. Fig. 1)

8.74 $$\begin{aligned} \int \limits _{0}^{E_\mathrm{max}} \mathrm{d}E_4 \int \limits _{0}^{E_4} \mathrm{d} E_5&= \int \limits _{0}^{E_\mathrm{max}} E_{45}\;\mathrm{d} E_{45} \int \limits _{0}^{1/2} \mathrm{d} z\nonumber \\&\quad + \int \limits _{E_\mathrm{max}}^{2 E_\mathrm{max}} E_{45}\; \mathrm{d} E_{45} \int \limits _{1-E_\mathrm{max}/E_{45}}^{1/2} \mathrm{d} z. \end{aligned}$$

Following this separation, we split the integral into two parts

8.75 $$\begin{aligned}&\big \langle \big [I-SS\big ] \big [I-S_5\big ]\theta ^{(b)} C_{45} w^{14,15} [\mathrm{d}g_{5}] F_{LM}(1,2,4,5)\big \rangle \nonumber \\&\quad =I_{A}+I_{B}, \end{aligned}$$

where the integral $I_A$ corresponds to the region $E_{45}<E_\mathrm{max}$ and the integral $I_B$ to the region $E_{45}>E_\mathrm{max}$; see Fig. 1. We obtain an integral representation for $I_A$ starting from Eq. (8.72), changing variables $(E_4,E_5)\rightarrow (E_{45},z)$ in the first term, and $E_5=z E_4$ in the second term in Eq. (8.72) and, finally, renaming $E_{45}\rightarrow E_4$. We obtain

8.76 $$\begin{aligned} I_A&=-\frac{[\alpha _\mathrm{s}]}{2\epsilon }N_\epsilon ^{(b)} \int [\mathrm{d}g_{4}] \tilde{w}_{4||5}^{14,15} x_3^{-\epsilon }(1-x_3)^{\epsilon } E_4^{-2\epsilon } \int \limits _0^1\frac{\mathrm{d}z}{z^{1+2\epsilon }} \nonumber \\&\quad \times \bigg \{z(1-z)^{-2\epsilon } \bigg [ -g_{\mu \nu } P_{gg}(z,\epsilon ) + \epsilon P^\perp _{gg}(z) r_\mu r_\nu \bigg ]\nonumber \\&\quad \times \big [I-S_{4}\big ]F_{LM}^{\mu \nu }(1,2,4)\theta (1/2 - z ) \nonumber \\&\quad -2C_\mathrm{A}\big [I-S_4\big ]F_{LM}(1,2,4)\bigg \}. \end{aligned}$$

To compute $I_B$, we notice that only the soft $S_{45}F_{LM}^{\mu \nu }(1,2,45)$ term of Eq. (8.72) contributes. Again renaming $E_{45} \rightarrow E_4$ and using Eq. (8.71), we obtain

8.77 $$\begin{aligned} I_B&=\frac{[\alpha _\mathrm{s}]^2}{2\epsilon }N_\epsilon ^{(b)} \int \limits _{E_\mathrm{max}}^{2E_\mathrm{max}} \frac{\mathrm{d}E_4}{E_4^{1+4\epsilon }} \nonumber \\&\quad \times \int \limits _0^1 \frac{2 \mathrm{d}x_3}{\big [4x_3(1-x_3)\big ]^\epsilon } \tilde{w}_{4||5}^{14,15} x_3^{-\epsilon }(1-x_3)^{\epsilon }\nonumber \\&\quad \times \int \limits _{z_4}^{1/2} \frac{\mathrm{d}z}{z^{1+2\epsilon }} z(1-z)^{-2\epsilon } \bigg [ P_{gg}(z,\epsilon ) + \epsilon P_{gg}(z,k_{\perp }) \bigg ]\nonumber \\&\quad \times \frac{2C_\mathrm{F}\rho _{12}}{\rho _{14}\rho _{24}}F_{LM}(1,2), \end{aligned}$$

where $z_4 = 1-E_4/E_\mathrm{max}$ and we expressed $[\mathrm{d}g_{4}]$ as an integral over energy $E_4$ and the angular integration variable $x_3 = \rho _{14}/2$. We have also integrated over $d-3$ angular variables that do not appear in the hard matrix element and in the splitting function.

Finally, we consider sector (*d*). We need to compute

8.78 $$\begin{aligned}&\big \langle \big [I-SS\big ] \big [I-S_5\big ]\theta ^{(d)} C_{45} w^{14,15} [\mathrm{d}g_{4}] F_{LM}(1,2,4,5)\big \rangle . \end{aligned}$$

The calculation is similar to what we just described for sector (*b*), apart from the following modifications of the integration boundaries:

8.79 $$\begin{aligned}&I_A:\,\,\theta (1/2-z) \rightarrow \theta (1-z) \;\theta (z-1/2), \nonumber \\&I_B:\,\,\theta ( z - z_4) \;\theta (1/2-z) \rightarrow \theta (z-1/2) \; \theta (1-z_4- z). \end{aligned}$$

Incorporating these changes in Eqs. (8.76) and (8.77) provides us with the result for sector (*d*).

Significant simplifications occur if the results for the two sectors are added; this happens because the *z*-integration boundaries in sectors (*b*) and (*d*) complement each other. Also, for both $I_A$ and $I_B$ the *z*-integration decouples from the rest and can be performed independently of the hard matrix element. In $I_A$, it yields

8.80 $$\begin{aligned} I_A^{(b)+(d)}&=\frac{[\alpha _\mathrm{s}]}{\epsilon } \bigg \langle \tilde{w}_{4||5}^{14,15} \left( \frac{\rho _{14}}{2}\right) ^{-\epsilon }\left( 1-\frac{\rho _{14}}{2}\right) ^{\epsilon } E_4^{-2\epsilon } \nonumber \\&\quad \times \bigg [ \tilde{\gamma }_g(\epsilon ) \big [I-S_4\big ]F_{LM}(1,2,4) + \epsilon \tilde{\gamma }_g(\epsilon ,k_\perp )\nonumber \\&\quad \times \big [I-S_4\big ]r_\mu r_\nu F_{LM}^{\mu \nu }(1,2,4) \bigg ]\bigg \rangle , \end{aligned}$$

where we used $x_3 = \rho _{14}/2$ and the constants $\tilde{\gamma }_g(\epsilon )$, $\tilde{\gamma }_g(\epsilon ,k_\perp )$ are defined in Eq. (A.19). In the integral $I_B$ the hard matrix element is that of the leading-order process which implies that integration over all variables related to radiated gluons can be explicitly performed. We find

8.81 $$\begin{aligned} I_B^{(b)+(d)}&=\frac{[\alpha _\mathrm{s}]^2}{\epsilon }E_\mathrm{max}^{-4\epsilon } \int \limits _0^1\frac{2\mathrm{d}x_3}{\big [4x_3(1-x_3)\big ]^\epsilon } \tilde{w}_{4||5}^{14,15} x_3^{-\epsilon }\nonumber \\&\quad \times (1-x_3)^{\epsilon } \delta _g(\epsilon ) \frac{ 2C_\mathrm{F}\rho _{12}}{\rho _{14}\rho _{24}} \big \langle F_{LM}(1,2)\big \rangle , \end{aligned}$$

with $\delta _g$ defined in Eq. (A.20).

#### Summing double- and triple-collinear partitions

Summing up the above results, we obtain an intermediate representation of $\langle F_{LM}^{s_rc_s} \rangle$. We write it as a sum of four terms,

8.82 $$\begin{aligned} \langle F_{LM}^{s_rc_s} \rangle&= \Bigg \{ \sum _{(ij)\in dc} \left\langle \big [I-SS\big ]\big [I-S_5\big ] \bigg [ C_{4i} [\mathrm{d}g_{4}] + C_{5j}[\mathrm{d}g_{5}] \bigg ]\right. \nonumber \\&\quad \times \left. w^{i4,j5}F_{LM}(1,2,4,5) \right\rangle +\sum _{i\in tc} \bigg \langle \big [I-SS\big ]\big [I-S_5\big ] \nonumber \\&\quad \times \bigg [ \theta ^{(a)} C_{5i} + \theta ^{(b)} C_{45} + \theta ^{(c)} C_{4i} + \theta ^{(d)} C_{45} \bigg ]\nonumber \\&\quad \times [\mathrm{d}g_{4}] [\mathrm{d}g_{5}] w^{i4,i5}F_{LM}(1,2,4,5) \bigg \rangle \Bigg \}\nonumber \\&=\big \langle \mathcal C_1(z\cdot 1,2,4)\big \rangle + \big \langle \mathcal C_2(1,z\cdot 2,4)\big \rangle \nonumber \\&\quad + \big \langle \mathcal C_3(1,2,4)\big \rangle + \big \langle \mathcal C_4(1,2,4)\big \rangle . \end{aligned}$$

These terms are defined as

8.83 $$\begin{aligned}&\big \langle \mathcal C_1(z\cdot 1,2,4)\big \rangle = -\frac{[\alpha _\mathrm{s}] s^{-\epsilon }}{\epsilon } \nonumber \\&\quad \times \int \limits _{0}^{1}\frac{\mathrm{d}z}{(1-z)^{1+2\epsilon }}\Bigg \langle \left( \tilde{w}^{14,15}_{5||1}\left( \frac{\rho _{41}}{4}\right) ^{-\epsilon } + \tilde{w}^{24,15}_{5||1}\right) \nonumber \\&\quad \times \bigg ( \left[ I- \theta ( z_4 - z) S_4 \right] \hat{\mathcal P}_{qq}^{(-)}F_{LM}(z\cdot 1,2,4) \nonumber \\&\quad +2C_\mathrm{F}\theta ( z_4 - z)\big [I- S_4\big ] F_{LM}(1,2,4)\bigg ) \Bigg \rangle , \end{aligned}$$

8.84 $$\begin{aligned}&\bigg \langle \mathcal C_2(1,z \cdot 2,4)\bigg \rangle = -\frac{[\alpha _\mathrm{s}] s^{-\epsilon }}{\epsilon }\nonumber \\&\quad \times \int \limits _{0}^{1} \frac{\mathrm{d}z}{(1-z)^{1+2\epsilon }} \Bigg \langle \left( \tilde{w}^{24,25}_{5||2}\left( \frac{\rho _{42}}{4}\right) ^{-\epsilon } + \tilde{w}^{14,25}_{5||2}\right) \nonumber \\&\quad \times \bigg ( \left[ I- \theta ( z_4 - z) S_4\right] \hat{\mathcal P}_{qq}^{(-)}F_{LM}(1,z\cdot 2,4) \nonumber \\&\quad +2C_\mathrm{F}\theta (z_4 - z)\big [I-S_4\big ] F_{LM}(1,2,4)\bigg ) \Bigg \rangle , \end{aligned}$$

8.85 $$\begin{aligned} \bigg \langle \mathcal C_3(1, 2,4)\bigg \rangle&= \frac{[\alpha _\mathrm{s}] }{\epsilon }\Bigg \langle \left[ \tilde{w}_{4||5}^{14,15} \left( \frac{\rho _{14}}{2}\right) ^{-\epsilon }\left( 1-\frac{\rho _{14}}{2}\right) ^{\epsilon }\right. \nonumber \\&\quad \left. + \tilde{w}_{4||5}^{24,25} \left( \frac{\rho _{24}}{2}\right) ^{-\epsilon }\left( 1-\frac{\rho _{24}}{2}\right) ^{\epsilon } \right] \nonumber \\&\quad \times E_4^{-2\epsilon } \big [I-S_4\big ] \bigg [ \tilde{\gamma }_g(\epsilon ) F_{LM}(1,2,4)\nonumber \\&\quad +\epsilon \tilde{\gamma }_g(\epsilon ,k_{\perp }) r_\mu r_\nu F_{LM}^{\mu \nu }(1,2,4) \bigg ]\Bigg \rangle , \end{aligned}$$

8.86 $$\begin{aligned} \bigg \langle \mathcal C_4(1,2,4)\bigg \rangle&= \frac{[\alpha _\mathrm{s}]^2 E_\mathrm{max}^{-4\epsilon } }{\epsilon } \;\delta _g(\epsilon ) \int \frac{\mathrm{d}\Omega _{(d-1),4}}{\Omega _{d-2}}\nonumber \\&\quad \times \bigg [\tilde{w}_{4||5}^{14,15} \left( \frac{\rho _{14}}{2}\right) ^{-\epsilon }\left( 1-\frac{\rho _{14}}{2}\right) ^{\epsilon }\nonumber \\&\quad + \tilde{w}_{4||5}^{24,25} \left( \frac{\rho _{24}}{2}\right) ^{-\epsilon } \left( 1-\frac{\rho _{24}}{2}\right) ^{\epsilon }\bigg ]\nonumber \\&\quad \times \left[ 2C_\mathrm{F}\frac{\rho _{12}}{\rho _{14}\rho _{24}}\right] \big \langle F_{LM}(1,2)\big \rangle , \end{aligned}$$

with

8.87 $$\begin{aligned} \frac{\mathrm{d}\Omega _{d-1,4}}{\Omega _{d-2}}=\mathrm{d}\cos \theta \left( \sin ^2\theta \right) ^{-\epsilon }= \frac{2\mathrm{d}x_3}{\big [4x_3(1-x_3)\big ]^\epsilon }. \end{aligned}$$

### The single-collinear term: extracting the last singularities 

The four contributions to $\langle F_{LM}^{s_rc_s} \rangle$ described at the end of the previous section require further manipulations because they cannot be expanded in series of $\epsilon$ as they are. Indeed, all of them exhibit collinear singularities in the limits 4||1 and 4||2, which need to be extracted before expansion in $\epsilon$ becomes possible. To deal with this issue, we again rewrite the identity operator through collinear projections. For example, we write

8.88 $$\begin{aligned} \big \langle \mathcal C_{1}(z \cdot 1, 2, 4) \big \rangle= & {} \big \langle ( C_{41} + C_{42} ) \mathcal C_{1}(z \cdot 1, 2, 4) \big \rangle \nonumber \\&+\big \langle ( I - C_{41} - C_{42} ) \mathcal C_{1}(z \cdot 1, 2, 4) \big \rangle .\nonumber \\ \end{aligned}$$

The first two terms can be further simplified by considering respective collinear limits; the last term is regulated and can be Taylor-expanded in $\epsilon$. The single-collinear subtraction term can be analyzed in the same way as all the other collinear limits discussed previously. The only new element here is the action of the collinear projection operators on the spin-correlated part. Using the explicit expression for the vector *r* in Eq. (8.62) we find

8.89 $$\begin{aligned} E_4^2\rho _{41} C_{41}r_\mu r_\nu F_{LM}^{\mu \nu }(1,2,4) = g_{s,b}^2 C_\mathrm{F}\frac{(1+z)^2}{2z}F_{LM}(z\cdot 1,2), \end{aligned}$$

where *z* is defined in the usual way $z = 1- E_4/E_1$. Taking this into account, after tedious but straightforward calculations we arrive at

$$\begin{aligned}&\langle F_{LM}^{s_rc_s} \rangle = \Bigg \{ \frac{[\alpha _\mathrm{s}]}{\epsilon }\Bigg \langle E_4^{-2\epsilon } \hat{\mathcal O}_\mathrm{NLO}\Delta _{4||5}\nonumber \\&\quad \times \bigg [ -g_{\mu \nu } \tilde{\gamma }_g + \epsilon \tilde{\gamma }_g^\perp r_\mu r_\nu \bigg ] F_{LM}^{\mu \nu }(1,2,4)\Bigg \rangle \nonumber \\&\quad -\frac{[\alpha _\mathrm{s}]}{\epsilon }\Bigg \langle s^{-\epsilon }\left[ \frac{(E_4/E_1)^{-2\epsilon }-1}{2\epsilon }\right] \nonumber \\&\quad \times 2C_\mathrm{F}\hat{\mathcal O}_\mathrm{NLO}\big [\Delta _{5||1}+\Delta _{5||2}\big ] F_{LM}(1,2,4)\Bigg \rangle \nonumber \\&\quad -\frac{[\alpha _\mathrm{s}]s^{-\epsilon }}{\epsilon }\int \limits _0^1 \mathrm{d}z\; \mathcal P_{qq,RR_2}(z)\nonumber \\&\quad \times \Bigg \langle \hat{\mathcal O}_\mathrm{NLO}\frac{\Delta _{5||1} F_{LM}(z\cdot 1,2,4) + \Delta _{5||2}F_{LM}(1,z\cdot 2,4) }{z} \Bigg \rangle \nonumber \\&\quad -\frac{[\alpha _\mathrm{s}]^2}{\epsilon }2C_\mathrm{F}\left[ \frac{s}{2}\right] ^{-2\epsilon } \int \limits _0^1 \mathrm{d}z\; \mathcal P_{qq,RR_3}(z) \int [d\rho _{14}] \hat{\mathcal{O}}_C S_{12,\rho }^{(4)} \Delta _{5||1}\nonumber \\&\quad \times \left\langle \frac{F_{LM}(z\cdot 1,2)}{z}\right\rangle -\frac{[\alpha _\mathrm{s}]^2}{\epsilon }2C_\mathrm{F}\left[ \frac{s}{2}\right] ^{-2\epsilon } \int \limits _0^1 \mathrm{d}z\; \mathcal P_{qq,RR_3}(z) \nonumber \\&\quad \times \int [d\rho _{24}] \hat{\mathcal{O}}_C S_{12,\rho }^{(4)}\Delta _{5||2}\times \left\langle \frac{F_{LM}(1,z\cdot 2)}{z} \right\rangle \Bigg \}+\Bigg \{\frac{[\alpha _\mathrm{s}]^2 s^{-2\epsilon }}{\epsilon ^2}\nonumber \\&\quad \times \int \limits _0^1 \mathrm{d}z\Bigg ( \frac{1}{2^{1-\epsilon }} \frac{\Gamma (1-2\epsilon )\Gamma (1-\epsilon )}{\Gamma (1-3\epsilon )} \big [\mathcal P_{qq}\otimes \mathcal P_{qq}\big ]_{RR}(z) \nonumber \\&\quad +\frac{\Gamma ^2(1-\epsilon )}{\Gamma (1-2\epsilon )}C_\mathrm{F}\left[ \frac{(E_\mathrm{max}/E_1)^{-2\epsilon }-1}{\epsilon }\right] \mathcal P_{qq,RR_2}(z) \nonumber \\&\quad +\left[ 2^{\epsilon } \frac{\Gamma (1-2\epsilon )\Gamma (1-\epsilon )}{\Gamma (1-3\epsilon )} +2\frac{\Gamma ^2(1-\epsilon )}{\Gamma (1-2\epsilon )}\right] C_\mathrm{F}\mathcal P_{qq,RR_{3+4}}(z) \nonumber \\ \end{aligned}$$

8.90 $$\begin{aligned}&\quad -\frac{1}{2^{1-2\epsilon }}\frac{\Gamma (1-2\epsilon )\Gamma (1-\epsilon )}{\Gamma (1-3\epsilon )}\nonumber \\&\quad \times \bigg [\tilde{\gamma }_g \mathcal P_{qq,RR_1}(z) + \epsilon \tilde{\gamma }_g^\perp \mathcal P_{qq,RR_5}(z)\bigg ]\Bigg )\nonumber \\&\quad \times \bigg \langle F_{LM}^z(1,2) \bigg \rangle +2 \frac{[\alpha _\mathrm{s}]^2 s^{-2\epsilon } }{\epsilon ^2}\frac{\Gamma ^2(1-\epsilon )}{\Gamma (1-2\epsilon )} \nonumber \\&\quad \times \int \limits _0^1 \mathrm{d}z~ \mathrm{d}\bar{z} ~\mathcal P_{qq,RR_2}(z) \mathcal P_{qq,RR_2}(\bar{z})\nonumber \\&\quad \times \left\langle \frac{F_{LM}(z\cdot 1,\bar{z}\cdot 2)}{z\bar{z}}\right\rangle +[\alpha _\mathrm{s}]^2C_\mathrm{F}\delta _g(\epsilon ) \nonumber \\&\quad \times \bigg [ \frac{1}{\epsilon } \int [d\rho _{14}] \hat{\mathcal{O}}_C S_{12,\rho }^{(4)}\Delta _{4||5} + \frac{1}{\epsilon } \int [d\rho _{24}] \hat{\mathcal{O}}_C S_{12,\rho }^{(4)}\Delta _{4||5} \nonumber \\&\quad -\frac{2^{1-2\epsilon }}{\epsilon ^2}\frac{\Gamma (1-2\epsilon )\Gamma (1-\epsilon )}{\Gamma (1-3\epsilon )}\bigg ] \big \langle E_\mathrm{max}^{-4\epsilon }F_{LM}(1,2)\big \rangle \Bigg \}. \end{aligned}$$

In the above equation, we used the following notation:

8.91 $$\begin{aligned}{}[d\rho _{ij}]&= \mathrm{d}\cos \theta _{ij}\; (\sin ^2\theta _{ij})^{-\epsilon },\quad \cos \theta _{ij} = 1 - \rho _{ij}.\nonumber \\ \Delta _{5||1}&= \left( \tilde{w}^{14,15}_{5||1}\left( \frac{\rho _{41}}{4}\right) ^{-\epsilon } + \tilde{w}^{24,15}_{5||1}\right) = 1 + \mathcal O(\epsilon ),\nonumber \\ \Delta _{5||2}&= \left( \tilde{w}^{24,25}_{5||2}\left( \frac{\rho _{42}}{4}\right) ^{-\epsilon } + \tilde{w}^{14,25}_{5||2}\right) = 1 + \mathcal O(\epsilon ),\nonumber \\ \Delta _{4||5}&= \left[ \tilde{w}_{4||5}^{14,15} \left( \frac{\rho _{14}}{2}\right) ^{-\epsilon }\left( 1-\frac{\rho _{14}}{2}\right) ^{\epsilon } \right. \nonumber \\&\left. \quad + \tilde{w}_{4||5}^{24,25} \left( \frac{\rho _{24}}{2}\right) ^{-\epsilon }\left( 1-\frac{\rho _{24}}{2}\right) ^{\epsilon } \right] = 1 + \mathcal O(\epsilon ). \end{aligned}$$

The relevant splitting functions are defined in Appendix A. Also,

8.92 $$\begin{aligned} F_{LM}^z(1,2)&\equiv \frac{ F_{LM}(z\cdot 1,2)+F_{LM}(1,z\cdot 2)}{z},\nonumber \\ \hat{\mathcal{O}}_C&\equiv I - C_{41} - C_{42},\nonumber \\ \hat{\mathcal O}_\mathrm{NLO}&\equiv \big [I-S_4\big ]\big [I-C_{41}-C_{42}\big ], \end{aligned}$$

and

8.93 $$\begin{aligned} S_{12,\rho }^{(4)}\equiv \frac{\rho _{12}}{\rho _{14}\rho _{24}}. \end{aligned}$$

We note that in Eq. (8.90) the first curly bracket is fully regulated, while the second contains subtraction terms. Note also that since $\Delta _{i||j} = 1+\mathcal O(\epsilon )$, if we are only interested in the $1/\epsilon$ poles, we can substitute $\Delta _{i||j}\rightarrow 1$ in Eq. (8.90). We also note that, for the process of interest, terms that contain $\mathcal{O}_C S_{12,\rho }^{(4)}$ can easily be integrated over the relative angles of the gluon $g_4$ with respect to the collision axis. We find

8.94 $$\begin{aligned} \int [\mathrm{d}\rho _{41}] \mathcal{O}_C S_{12,\rho }^{(4)}\Delta _{5||1}&= \int [\mathrm{d}\rho _{42}] \mathcal{O}_C S_{12,\rho }^{(4)}\Delta _{5||2}\nonumber \\&= (1+\ln 2)\epsilon + \mathcal O(\epsilon ^2), \nonumber \\ \int [\mathrm{d}\rho _{41}] \mathcal{O}_C S_{12,\rho }^{(4)}\Delta _{4||5}&= \int [\mathrm{d}\rho _{41}] \mathcal{O}_C S_{12,\rho }^{(4)}\Delta _{4||5}\nonumber \\&= \left( 2-\frac{\pi ^2}{3}\right) \epsilon + \mathcal O(\epsilon ^2). \end{aligned}$$

We will use these results when presenting the final formula for the double-real contribution.

### The double-unresolved collinear limit: double collinear

We now turn to the term $\langle F_{LM}^{s_rc_t} \rangle$ and begin by considering the contribution of the double-collinear partitions. It reads

8.95 $$\begin{aligned}&-\sum _{(ij)\in dc}\bigg \langle \big [I-SS\big ]\big [I-S_5\big ]\nonumber \\&\quad \quad \times C_{4i}C_{5j}[\mathrm{d}g_{4}][\mathrm{d}g_{5}] w^{i4,j5}F_{LM}(1,2,4,5) \bigg \rangle \nonumber \\&\quad =-\bigg \langle \big [I-SS\big ]\big [I-S_5\big ]\big [C_{41}C_{52}+C_{51}C_{42}\big ]\nonumber \\&\quad \quad \times [\mathrm{d}g_{4}][\mathrm{d}g_{5}]F_{LM}(1,2,4,5) \bigg \rangle . \end{aligned}$$

Note that, following our notational convention, the collinear projection operators act on the phase space elements $[\mathrm{d}g_{4}]$ and $[\mathrm{d}g_{5}]$.

We begin with the $C_{41}C_{52}$ term. Introducing

8.96 $$\begin{aligned} E_4 = (1-z)E_1,~~~ E_5=(1-\bar{z})E_2, \end{aligned}$$

and calculating collinear limits we obtain

8.97 $$\begin{aligned}&E_4^2 E_5^2 \rho _{14} \rho _{25} C_{41}C_{52}F_{LM}(1,2,4,5)\nonumber \\&\quad = g_{s,b}^4 \mathcal P_{qq}(z)\mathcal P_{qq}(\bar{z}) F_{LM}(z\cdot 1,\bar{z}\cdot 2). \end{aligned}$$

Since the momenta of gluons $g_{4}$ and $g_5$ decouple from each other, we find

8.98 $$\begin{aligned} SSC_{41}C_{52} F_{LM}(1,2,4,5)&= SSS_5 C_{41}C_{52} F_{LM}(1,2,4,5)\nonumber \\&= \frac{4g_{s,b}^4C_\mathrm{F}^2}{E_4^2 E_5^2 \rho _{14} \rho _{15} } F_{LM}(1,2). \end{aligned}$$

As the result, the original expression simplifies

8.99 $$\begin{aligned}&\big [I-SS\big ]\big [I-S_5\big ] C_{41}C_{52}F_{LM}(1,2,4,5)\nonumber \\&\quad =\big [I-S_5\big ]C_{41}C_{52}F_{LM}(1,2,4,5). \end{aligned}$$

Performing the angular integrations and accounting for the hierarchy of energies $E_4 > E_5$, we obtain

8.100 $$\begin{aligned}&-\big \langle \big [I-SS\big ]\big [I-S_5\big ] C_{41}C_{52}[\mathrm{d}g_{4}][\mathrm{d}g_{5}]F_{LM}(1,2,4,5) \big \rangle \nonumber \\&\quad = -\frac{[\alpha _\mathrm{s}]^2 s^{-2\epsilon }}{\epsilon ^2} \int \limits _{0}^{1} \frac{\mathrm{d}z}{(1-z)^{1+2\epsilon }} \int \limits _{z}^{1} \frac{\mathrm{d}\bar{z}}{(1-\bar{z})^{1+2\epsilon }} \nonumber \\&\qquad \times \big \langle \mathcal P_{qq}(z)\mathcal P_{qq}(\bar{z}) F_{LM}(z\cdot 1,\bar{z}\cdot 2)\nonumber \\&\quad \quad -2C_\mathrm{F}\mathcal P_{qq}(z)F_{LM}(z\cdot 1,2) \big \rangle , \end{aligned}$$

where, as usual, the *z* integrals do not need a lower cut-off whenever *z* is present in $F_{LM}$.

The term with collinear operators $C_{51}C_{42}$ in Eq. (8.95) can be simplified in a similar way. Combining the two contributions, we obtain

8.101 $$\begin{aligned}&-\big \langle \big [I-SS\big ]\big [I-S_5\big ] \big [C_{41}C_{52}+C_{51}C_{42}\big ]\nonumber \\&\qquad \times [\mathrm{d}g_{4}][\mathrm{d}g_{5}]F_{LM}(1,2,4,5) \big \rangle \nonumber \\&\quad =-\frac{[\alpha _\mathrm{s}]^2 s^{-2\epsilon } }{\epsilon ^2} \Bigg \{\int \limits _{0}^{1} \frac{\mathrm{d}z}{(1-z)^{1+2\epsilon }} \frac{\ d\bar{z}}{(1-\bar{z})^{1+2\epsilon }} \nonumber \\&\qquad \times \mathcal P_{qq}(z)\mathcal P_{qq}(\bar{z})\big \langle F_{LM}(z\cdot 1,\bar{z}\cdot 2)\big \rangle \nonumber \\&\qquad -2C_\mathrm{F}\int \limits _{0}^{1} \frac{\mathrm{d}z}{(1-z)^{1+2\epsilon }}\mathcal P_{qq}(z)\bigl <F_{LM}(z\cdot 1,2) \nonumber \\&\qquad + F_{LM}(1,z\cdot 2)\bigr >\times \int \limits _{z}^{1}\frac{\mathrm{d}\bar{z}}{(1-\bar{z})^{1+2\epsilon }}\Bigg \}. \end{aligned}$$

We can rewrite Eq. (8.101) to ensure that all singularities that appear in *z* and $\bar{z}$ integrals are regulated with the plus-prescription. This gives the final result for the double-collinear contribution

8.102 $$\begin{aligned}&-\big \langle \big [I-SS\big ]\big [I-S_5\big ] \big [C_{41}C_{52}+C_{51}C_{42}\big ]\nonumber \\&\qquad \times [\mathrm{d}g_{4}][\mathrm{d}g_{5}]F_{LM}(1,2,4,5) \big \rangle \nonumber \\&\quad =-\frac{[\alpha _\mathrm{s}]^2 s^{-2\epsilon } }{\epsilon ^2}\Bigg \{ \int \limits _0^1 \mathrm{d}z\; \mathrm{d}\bar{z} ~ \mathcal P_{qq,RR_2}(z) \mathcal P_{qq,RR_2}(\bar{z})~\nonumber \\&\qquad \times \left\langle \frac{F_{LM}(z\cdot 1,\bar{z}\cdot 2)}{z\bar{z}} \right\rangle -\int \limits _0^1 \mathrm{d}z\; \mathcal P_{qq,RR_6}(z)\nonumber \\&\qquad \times \left\langle \frac{F_{LM}(z\cdot 1,2) + F_{LM}(1,\bar{z}\cdot 2) }{z} \right\rangle \Bigg \}. \end{aligned}$$

The relevant splitting functions are given in Appendix A.

### The double-unresolved collinear limit: triple collinear

In this section, we consider the contribution of the triple-collinear partitions to $\langle F_{LM}^{s_r c_t} \rangle$. It reads

8.103 $$\begin{aligned}&\sum _{i\in tc} \bigg \langle \big [I-SS\big ]\big [I-S_5\big ] \bigg [ \theta ^{(a)} CC_i\big [I-C_{5i}\big ]\nonumber \\&\quad + \theta ^{(b)} CC_i\big [I-C_{45}\big ] \nonumber \\&\quad + \theta ^{(c)} CC_i\big [I-C_{4i}\big ]+ \theta ^{(d)} CC_i\big [I-C_{45}\big ] \bigg ]\nonumber \\&\quad \times [\mathrm{d}g_{4}] [\mathrm{d}g_{5}] w^{i4,i5}F_{LM}(1,2,4,5) \bigg \rangle . \end{aligned}$$

This contribution always contains the triple-collinear projection operator $CC_i$ that acts on the hard matrix elements. For $i =1$, this gives, schematically,

8.104 $$\begin{aligned} CC_1 F_{LM}(1,2,4,5)&= \left( \frac{2}{s_{145}}\right) ^2 P_{ggq}(1,4,5)\nonumber \\&\quad \times F_{LM}(1-4-5,2), \end{aligned}$$

where $$s_{145} = (p_1-p_4-p_5)^2$$ and $$P_{ggq}(1,4,5) $$ is the known triple-collinear splitting function [75, 79, 80]. The reduced matrix element in Eq. (8.104) has to be evaluated in the exact collinear limit, i.e. $$p_{1-4-5}\equiv (E_1-E_4-E_5)/E_1\cdot p_1$$. Other projection operators that appear in Eq. (8.103) provide subtractions that are needed to make the triple-collinear splitting function integrable over the unresolved parts of the $$(g_4,g_5)$$ phase space. For definiteness, we focus here on the triple-collinear partition where gluons are emitted along the direction of the incoming quark with momentum $$p_1$$; this corresponds to taking $$i=1$$ in Eq. (8.103).

To proceed, we note that the damping factors in Eq. (8.103) can be removed since the collinear projection operator $CC_1$ acting on them yields 1. Next, we need to study the triple-collinear limit of the angular phase space. The generic phase space parametrization is described in Appendix B and we use it to compute the triple-collinear limits. We stress that since the phase space parametrization changes from sector to sector, we need to consider all the four sectors separately.

Without going into further detail of the angular integration, it is clear that once this integration is performed, each sector in Eq. (8.103) provides the following contribution to the final integral over energies:

8.105 $$\begin{aligned}&\int \theta ^{(k)} CC_1 \big [I-C_{ij}\big ] \mathrm{d}\Omega _{45}^{(k)} F_{LM}(1,2,4,5)\nonumber \\&\quad \equiv [\alpha _\mathrm{s}]^2 T_C^{(k)}(E_1,E_4,E_5)F_{LM}(1-4-5,2), \end{aligned}$$

where the auxiliary function $T_C^{(k)}$ in Eq. (8.105) is defined as

8.106 $$\begin{aligned}{}[\alpha _\mathrm{s}]^2 T_C^{(k)}(E_1,E_4,E_5)= & {} \int \theta ^{(k)} CC_1 \big [I-C_{ij}\big ]\; \mathrm{d}\Omega _{45}^{(k)} \;\nonumber \\&\times \frac{4 P_{ggq}(z_4,z_5,z_1,s_{45},s_{41},s_{51}) }{s^2_{145}}. \nonumber \\ \end{aligned}$$

We note that the reason that $CC_1$ is present in Eq. (8.106) is that it still has to act on the phase space; its action on the matrix element has already been accounted for and resulted in the factorized form of Eq. (8.105) and the appearance of the triple-collinear splitting function in Eq. (8.106). We use Eq. (8.105) to write

8.107 $$\begin{aligned}&\big \langle \big [I-SS\big ]\big [I-S_5\big ] \theta ^{(k)} CC_1\big [I-C_{ij}\big ] [\mathrm{d}g_{4}] [\mathrm{d}g_{5}]\nonumber \\&\quad \quad \times w^{i4,i5}F_{LM}(1,2,4,5)\big \rangle \bigg . \nonumber \\&\quad \equiv [\alpha _\mathrm{s}]^2\times \int \limits _{0}^{E_\mathrm{max}} \mathrm{d}E_4\; E_4^{1-2\epsilon } \int \limits _{0}^{E_4} \mathrm{d}E_5\; E_5^{1-2\epsilon } \big [I-SS\big ] \nonumber \\&\quad \quad \times \big [I-S_5\big ] T_C^{(k)}(E_1,E_4,E_5) \big \langle F_{LM}(145,2)\big \rangle , \end{aligned}$$

where $145 \equiv 1 - 4 -5$ in the collinear approximation.

In what follows, we discuss the integration over energies in Eq. (8.107). Our goal is to change variables in such a way that the argument of the hard matrix element becomes $z \cdot 1$; once this happens, Eq. (8.107) becomes a convolution of a hard matrix element with a splitting function. Although, in principle, changing variables in an integral is straightforward, it turns out that it is beneficial to do so in different ways in the four terms that appear in Eq. (8.107); for this reason, we consider them separately. We emphasize that since the individual contributions to Eq. (8.107) diverge, it is important to keep dimensional regularization in place until the end of the computation.

We begin with the term that contains the identity operator $I$ and change the variables as follows:

8.108 $$\begin{aligned} E_4 = E_1(1-z)\left( 1-\frac{r}{2}\right) ,\quad E_5 = E_1(1-z)\frac{r}{2}, \end{aligned}$$

with $r\in (0,1)$ and $z\in (0,1)$. We note that the lower integration boundary for *z* can be taken to be $z = 0$ because $F_{LM}(z\cdot 1,2)$ always appears in this contribution. As we already discussed several times, this automatically cuts off the integral over *z* at a proper minimal value. With this in mind, we write^9^

8.109 $$\begin{aligned}&\int \limits _{0}^{E_\mathrm{max}} \mathrm{d}E_4\; E_4^{1-2\epsilon } \int \limits _{0}^{E_4} \mathrm{d}E_5\; E_5^{1-2\epsilon } T_C(E_1,E_4,E_5)F_{LM}(145,2) \nonumber \\&\quad =\frac{E_1^{4-4\epsilon }}{2^{-2\epsilon }} \int \limits _0^1\frac{\mathrm{d}z}{(1-z)^{1+4\epsilon }} \frac{\mathrm{d}r}{r^{1+2\epsilon }} \frac{1}{\left( 1-\frac{r}{2}\right) ^{1+2\epsilon }}\nonumber \\&\qquad \times \bigg [ (1-z)^4 \left( \frac{r}{2}\right) ^2\left( 1-\frac{r}{2}\right) ^2T_C\left( E_1,E_1(1-z)\right. \nonumber \\&\qquad \left. \times \left( 1-\frac{r}{2}\right) , E_1(1-z)\frac{r}{2}\right) \bigg ] F_{LM}(z\cdot 1,2). \end{aligned}$$

Next, we consider the $S_5$ operator. It describes the limit where $E_5 \rightarrow 0$ at fixed $E_4$. Calculating this limit with the parametrization in Eq. (8.108) mixes *z* and *r* and, therefore, is inconvenient. A better way is to change the parametrization. We choose

8.110 $$\begin{aligned} E_4 = E_1(1-z), \quad E_5 = E_1(1-z)r, \end{aligned}$$

where $r\in (0,1)$. In principle, we should use $z>z_\mathrm{min}$ but, since *z* enters the hard matrix element, we can extend all the integrals to $z \in (0,1)$. We find

8.111 $$\begin{aligned}&\int \limits _{0}^{E_\mathrm{max}} \mathrm{d}E_4\; E_4^{1-2\epsilon } \int \limits _{0}^{E_4} \mathrm{d}E_5\; E_5^{1-2\epsilon } S_5T_C(E_1,E_4,E_5)\nonumber \\&\quad \times F_{LM}(1-4-5,2)= E_1^{4-4\epsilon }\nonumber \\&\quad \times \int \limits _0^1\frac{\mathrm{d}z}{(1-z)^{1+4\epsilon }} \frac{\mathrm{d}r}{r^{1+2\epsilon }}\; F_{LM}(z\cdot 1,2)\nonumber \\&\quad \times \bigg [(1-z)^4 r^2 T_C(E_1, E_1(1-z),E_1(1-z)r)\bigg ]_{r\rightarrow 0}. \end{aligned}$$

Note that the $r^2$ prefactor ensures that the $r\rightarrow 0$ limit of the square bracket exists.

We can also use the change of variables in Eq. (8.110) for terms with operators $SS$ and $SSS_5$. The only difference is that since in those terms *z* does not appear in $F_{LM}$, we have to keep the lower integration boundary at $z = z_\mathrm{min}$. We write

8.112 $$\begin{aligned}&\int \limits _{0}^{E_\mathrm{max}} \mathrm{d}E_4\; E_4^{1-2\epsilon } \int \limits _{0}^{E_4} \mathrm{d}E_5\; E_5^{1-2\epsilon } SST_C(E_1,E_4,E_5)\nonumber \\&\quad \times F_{LM}(1-4-5,2)= E_1^{4-4\epsilon }\nonumber \\&\quad \times \int \limits _{z_{\mathrm{min}}}^1\frac{\mathrm{d}z}{(1-z)^{1+4\epsilon }} \frac{\mathrm{d}r}{r^{1+2\epsilon }}\; F_{LM}(1,2) \nonumber \\&\quad \times \bigg [(1-z)^4 r^2 T_C(E_1,E_1(1-z),E_1(1-z)r)\bigg ]_{z\rightarrow 1}. \end{aligned}$$

Also in this case, the $(1-z)^4$ prefactor ensures the existence of the $z\rightarrow 1$ limit of the term in the square bracket. The term with an operator $SSS_5$ in Eq. (8.107) is obtained from Eq. (8.112) by taking the $r \rightarrow 0$ limit in the expression in square brackets.

To proceed, it is convenient to define two *z*-dependent functions and a constant

8.113 $$\begin{aligned} A_1(z)&\equiv \frac{z}{2^{-2\epsilon }} \int \limits _0^1 \frac{\mathrm{d}r}{r^{1+2\epsilon }} \frac{1}{\left( 1-\frac{r}{2}\right) ^{1+2\epsilon }}\nonumber \\&\quad \times \Bigg \{ \bigg [ (1-z)^4 \left( \frac{r}{2}\right) ^2\left( 1-\frac{r}{2}\right) ^2 \nonumber \\&\quad \times T_C\left( E_1,E_1(1-z)\left( 1-\frac{r}{2}\right) , E_1(1-z)\frac{r}{2}\right) \bigg ] \nonumber \\&\quad -\bigg [ (1-z)^4 \left( \frac{r}{2}\right) ^2\left( 1-\frac{r}{2}\right) ^2 T_C\left( E_1,E_1(1-z)\left( 1-\frac{r}{2}\right) , \right. \nonumber \\&\quad \times \left. E_1(1-z)\frac{r}{2}\right) \bigg ]_{r\rightarrow 0}\Bigg \}, \nonumber \\ A_2(z)&\equiv \frac{z}{2^{-2\epsilon }} \int \limits _0^1 \frac{\mathrm{d}r}{r^{1+2\epsilon }} \frac{1}{\left( 1-\frac{r}{2}\right) ^{1+2\epsilon }} \nonumber \\&\quad \times \bigg [ (1-z)^4 \left( \frac{r}{2}\right) ^2\left( 1-\frac{r}{2}\right) ^2 T_C\left( E_1,E_1(1-z)\left( 1-\frac{r}{2}\right) ,\right. \nonumber \\&\quad \times \left. E_1(1-z)\frac{r}{2}\right) \bigg ]_{r\rightarrow 0} \nonumber \\&\quad -z\int \limits _0^1 \frac{\mathrm{d}r}{r^{1+2\epsilon }} \bigg [(1-z)^4 r^2 T_C(E_1,E_1(1-z),E_1(1-z)r)\bigg ]_{r\rightarrow 0}, \nonumber \\ A_3&\equiv \int \limits _0^1 \frac{\mathrm{d}r}{r^{1+2\epsilon }} \Bigg \{ \bigg [(1-z)^4 r^2 T_C(E_1,E_1(1-z),E_1(1-z)r)\bigg ]\nonumber \\&\quad -\bigg [(1-z)^4 r^2 T_C(E_1,E_1(1-z),E_1(1-z)r)\bigg ]_{r\rightarrow 0} \Bigg \}_{z\rightarrow 1}. \end{aligned}$$

We can further simplify the function $A_2$ if we realize that the $S_5$ limit of the triple-collinear splitting function is homogeneous in $E_5$. This implies that the $r \rightarrow 0$ limit of the two $T_C$ functions in the formula for $A_2$ in Eq. (8.113) are related and can be combined. Changing variables $r \rightarrow r/2$ in the first term on the r.h.s. of the integral for $A_2$, we obtain

8.114 $$\begin{aligned} A_2(z)= & {} z \bigg [ \int \limits _0^{1/2} \frac{\mathrm{d}r}{r^{1+2\epsilon }}\frac{1}{(1-r)^{1+2\epsilon }} -\int \limits _0^1 \frac{\mathrm{d}r}{r^{1+2\epsilon }}\bigg ]\nonumber \\&\times \bigg [(1-z)^4 r^2 T_C(E_1,E_1(1-z),E_1(1-z)r)\bigg ]_{r\rightarrow 0}.\nonumber \\ \end{aligned}$$

Since the term in the square bracket no longer depends on *r* after the $r\rightarrow 0$ limit is taken, we can perform the *r* integrations in the first line to get

8.115 $$\begin{aligned} A_2(z)= & {} \frac{z}{2\epsilon } \left[ 1-\frac{\Gamma ^2(1-2\epsilon )}{\Gamma (1-4\epsilon )}\right] \nonumber \\&\times \bigg [(1-z)^4 r^2 T_C(E_1,E_1(1-z),E_1(1-z)r)\bigg ]_{r\rightarrow 0}. \nonumber \\ \end{aligned}$$

We can now write the result for the integral that we are interested in using $A_{1,2}(z)$ and $A_3$. We find^10^

8.116 $$\begin{aligned}&\big \langle \big [I-SS\big ]\big [I-S_5\big ] \theta ^{(k)} CC_1\big [I-C_{ij}\big ] [\mathrm{d}g_{4}] [\mathrm{d}g_{5}]\nonumber \\&\quad \times w^{i4,i5}F_{LM}(1,2,4,5)\big \rangle =[\alpha _\mathrm{s}]^2 E_1^{4-4\epsilon }\bigg .\nonumber \\&\quad \times \Bigg \{ \int \limits _0^1\mathrm{d}z\frac{ A_1^{(k)}(z)+A_2^{(k)}(z)}{(1-z)^{1+4\epsilon }} \left\langle \frac{F_{LM}(z\cdot 1,2)}{z}\right\rangle \nonumber \\&\quad - \int \limits _{z_\mathrm{min}}^1 \frac{A_3^{(k)} \mathrm{d}z}{(1-z)^{1+4\epsilon }} \big \langle F_{LM}(1,2)\big \rangle \Bigg \} . \end{aligned}$$

This integral can be re-written in such a way that all the $z \rightarrow 1$ singularities are regulated by plus-prescriptions. We have already discussed how this can be done several times; for this reason, we do not repeat this discussion again and only present the result. It reads

8.117 $$\begin{aligned}&\big \langle \big [I-SS\big ]\big [I-S_5\big ] \theta ^{(k)} CC_1\big [I-C_{ij}\big ] [\mathrm{d}g_{4}] [\mathrm{d}g_{5}]\nonumber \\&\quad \times w^{i4,i5}F_{LM}(1,2,4,5) \big \rangle \bigg .= [\alpha _\mathrm{s}]^2 E_1^{4-4\epsilon } \nonumber \\&\quad \times \int \limits _{0}^{1} \mathrm{d}z \left[ R^{(k)}(z) + \frac{R^{(k)}_+}{[(1-z)^{1+4\epsilon }]_+} + R^{(k)}_\delta \delta (1-z) \right] \nonumber \\&\quad \times \left\langle \frac{F_{LM}(z \cdot 1, 2)}{z}\right\rangle , \end{aligned}$$

where8.118The functions $A^{(k)}_{1,2}(z)$ and the constant $A^{(k)}_3$ are given in Eqs. (8.113) and (8.115). We have also used the following notation:

8.119 $$\begin{aligned}&\frac{1}{[(1-z)^{1+4\epsilon }]_+} = \sum \limits _{n=0}^{\infty } \frac{(-4 \epsilon )^n}{n!} \mathcal{D}_n(z),\nonumber \\&F^{(k)}(r) = \bigg [(1-z)^4 r^2 T_C^{(k)}(E_1,E_1(1-z),E_1(1-z)r)\bigg ]_{z\rightarrow 1}. \end{aligned}$$

We are now in a position to write the contribution of this term in the final form

8.120 $$\begin{aligned}&\sum \limits _{k} \big \langle \big [I-SS\big ]\big [I-S_5\big ] \theta ^{(k)}CC_1\big [I-C_{ij}\big ]\nonumber \\&\quad \times [\mathrm{d}g_{4}] [\mathrm{d}g_{5}] w^{14,15}F_{LM}(1,2,4,5) \big \rangle = \frac{[\alpha _\mathrm{s}]^2}{\epsilon } \times \left[ \frac{s}{4}\right] ^{-2\epsilon } \sum \limits _{k}^{} \nonumber \\&\quad \times \int \limits _0^1 \mathrm{d}z \bigg [ R^{(k)}(z) + \frac{R_+^{(k)}}{\big [(1-z)^{1+4\epsilon }\big ]_+} +R_\delta ^{(k)} \delta (1-z)\bigg ]\nonumber \\&\quad \times \left\langle \frac{F_{LM}(z\cdot 1,2)}{z}\right\rangle . \end{aligned}$$

All the terms in Eq. (8.120) can be expanded in power series in the dimensional regularization parameter $\epsilon$. The functions $R^{(k)}(z)$ and the constants $R_+^{(k)}$ and $R^{(k)}_{\delta }$ are calculated numerically.

## Pole cancellation and finite remainders

We are now in position to discuss the final result for the NNLO QCD contribution to the cross section. We consider

9.1 $$\begin{aligned} \mathrm{d}\hat{\sigma }^\mathrm{NNLO} = \mathrm{d}\sigma ^\mathrm{RR} + \mathrm{d}\sigma ^\mathrm{RV} + \mathrm{d}\sigma ^\mathrm{VV} + \mathrm{d}\sigma ^\mathrm{ren} + \mathrm{d}\sigma ^\mathrm{CV}. \nonumber \\ \end{aligned}$$

All the different contributions to Eq. (9.1) were considered in the previous sections. It should be clear from these discussions that the result for the NNLO cross section is given by a linear combination of integrated matrix elements with different multiplicities, which may or may not be convoluted with generalized splitting functions. Since, for well-defined observables, the cancellation of soft and collinear divergences occurs point-by-point in the phase space, contributions proportional to $F_{LM}(1,2,4,5)$, $F_{LM}(1,2,4)$, $F_{LM}(z \cdot 1, 2, 4)$ etc. must be separately finite. For this reason, it is convenient to present the result for the NNLO QCD contribution to the cross section as a sum of seven terms

9.2 $$\begin{aligned} \mathrm{d} \hat{\sigma }^\mathrm{NNLO}&= \mathrm{d}\hat{\sigma }^\mathrm{NNLO}_{F_{LM}(1,2,4,5)} + \mathrm{d}\hat{\sigma }^\mathrm{NNLO}_{F_{LM}(1,2,4)} + \mathrm{d}\hat{\sigma }^\mathrm{NNLO}_{F_{LM}(z \cdot 1, \bar{z} \cdot 2) } \nonumber \\&\quad + \mathrm{d}\hat{\sigma }^\mathrm{NNLO}_{F_{LM}(z \cdot 1,2)} + \mathrm{d}\hat{\sigma }^\mathrm{NNLO}_{F_{LM}(1, z \cdot 2)} + \mathrm{d}\hat{\sigma }^\mathrm{NNLO}_{F_{LV}^\mathrm{fin}(1,2)} \nonumber \\&\quad + \mathrm{d}\hat{\sigma }^\mathrm{NNLO}_{F_{LM}(1,2)}, \end{aligned}$$

which are individually finite. Each of the individual terms in Eq. (9.2) has a subscript that indicates the highest multiplicity matrix element that it contains. Below we collect all the different contributions to $\mathrm{d}\hat{\sigma }^\mathrm{NNLO}$ and present finite remainders for terms with different multiplicities. For simplicity, we fix the arbitrary parameter $E_\mathrm{max}=\sqrt{s}/2$.

### Terms involving $F_{LM}(1,2,4,5)$

This contribution is the only one that involves the matrix element for $q \bar{q} \rightarrow V+gg$. We repeat here the result, already given in Eq. (8.11)

9.3 $$\begin{aligned}&\mathrm{d}\hat{\sigma }^\mathrm{NNLO}_{F_{LM}(1,2,4,5)} = \langle F_{LM}^{s_rc_r} \rangle = \sum _{(ij)\in dc}\bigg \langle \big [I-SS\big ]\big [I-S_5\big ] \nonumber \\&\quad \times \bigg [(I- C_{5j})(I-C_{4i})\bigg ]\nonumber \\&\quad \times [\mathrm{d}g_{4}][\mathrm{d}g_{5}] w^{i4,j5}F_{LM}(1,2,4,5) \bigg \rangle \nonumber \\&\quad +\sum _{i\in tc} \bigg \langle \big [I-SS\big ]\big [I-S_5\big ] \bigg [ \theta ^{(a)} \big [I-CC_i\big ]\big [I-C_{5i}\big ]\nonumber \\&\quad + \theta ^{(b)} \big [I-CC_i\big ]\big [I-C_{45}\big ]\nonumber \\&\quad + \theta ^{(c)} \big [I-CC_i\big ]\big [I-C_{4i}\big ]+ \theta ^{(d)} \big [I-CC_i\big ]\big [I-C_{45}\big ] \bigg ] \nonumber \\&\quad \times [\mathrm{d}g_{4}] [\mathrm{d}g_{5}] w^{i4,i5}F_{LM}(1,2,4,5) \bigg \rangle . \end{aligned}$$

It follows that $\mathrm{d}\hat{\sigma }^\mathrm{NNLO}_{F_{LM}(1,2,4,5)}$ is expressed through a combination of nested soft and collinear subtractions and can be directly computed in four dimensions.

### Terms involving $\hat{\mathcal{O}}_{NLO}F_{LM}(1,2,4)$

We continue with terms that involve $F_{LM}(1,2,4)$. They are present in the double-real contribution, Eqs. (8.33) and (8.90) and in the real-virtual contribution, Eq. (7.8); they are also found in terms that appear due to ultraviolet Eq. (5.1) and collinear Eq. (5.8) renormalizations of the next-to-leading order cross section. Extracting these terms, we observe that all the $1/\epsilon$ singularities cancel out. The finite remainder reads

9.4 $$\begin{aligned}&\mathrm{d} \hat{\sigma }^\mathrm{NNLO}_{124} = \frac{\alpha _\mathrm{s}(\mu )}{2\pi }\Bigg \{ \int \limits _0^1 \mathrm{d}z \bigg [4C_\mathrm{F}\tilde{\mathcal D}_1(z) - \hat{P}^{(0)}_{qq}(z) \ln \left( \frac{\mu ^2}{s}\right) - \hat{P}^{(\epsilon )}_{qq}(z) \bigg ] \nonumber \\&\quad \times \Bigg \langle \hat{\mathcal O}_\mathrm{NLO}\left[ \frac{F_{LM}(z\cdot 1,2,4)+F_{LM}(1,z\cdot 2,4)}{z}\right] \Bigg \rangle \nonumber \\&\quad + 2C_\mathrm{F}\int \limits _0^1 \mathrm{d}z\; \tilde{\mathcal D}_0(z) \times \Bigg \langle \ln \frac{\rho _{41}}{4}\hat{\mathcal O}_\mathrm{NLO} \frac{\tilde{w}_{5||1}^{15,14}F_{LM}(z\cdot 1,2,4)}{z} \nonumber \\&\quad + \ln \frac{\rho _{42}}{4}\hat{\mathcal O}_\mathrm{NLO} \frac{\tilde{w}_{5||2}^{25,24}F_{LM}(1,z\cdot 2,4)}{z} \Bigg \rangle \nonumber \\&\quad +C_\mathrm{F}\Bigg \langle \hat{\mathcal O}_\mathrm{NLO} \bigg [ \frac{2}{3}\pi ^2 -2 \ln \frac{2E_4}{\sqrt{s}}\ln \frac{\rho _{41}}{4} \tilde{w}_{5||1}^{14,15} \nonumber \\&\quad -2 \ln \frac{2E_4}{\sqrt{s}}\ln \frac{\rho _{42}}{4} \tilde{w}_{5||2}^{24,25} \bigg ]F_{LM}(1,2,4) \Bigg \rangle \nonumber \\&\quad +C_\mathrm{A}\Bigg \langle \hat{\mathcal O}_\mathrm{NLO} \bigg [ \frac{137}{18}-\frac{4}{3}\pi ^2+\frac{11}{6} \ln \frac{\mu ^2}{E_4^2} \nonumber \\&\quad -\frac{11}{6}\left( \ln \frac{\rho _{14}}{\rho _{24}} \tilde{w}_{4||5}^{14,15}+ \ln \frac{\rho _{24}}{\rho _{14}} \tilde{w}_{4||5}^{24,25} \right) \nonumber \\&\quad +\frac{3}{2}\ln \frac{2E_4}{\sqrt{s}} +\ln ^2\frac{2E_4}{\sqrt{s}}+ \frac{3}{4}\ln \frac{\rho _{14}\rho _{24}}{4}\nonumber \\&\quad +\ln \frac{2 E_4}{\sqrt{s}}\ln \frac{\rho _{14}\rho _{24}}{4} +\text {Li}_2\left( 1-\frac{\rho _{14}}{2}\right) \nonumber \\&\quad +\text {Li}_2\left( 1-\frac{\rho _{24}}{2}\right) \bigg ] F_{LM}(1,2,4)\Bigg \rangle \nonumber \\&\quad -\frac{C_\mathrm{A}}{3}\big \langle \hat{\mathcal O}_\mathrm{NLO} r_\mu r_\nu F_{LM}^{\mu \nu }(1,2,4)\big \rangle \Bigg \}+ \big \langle \hat{\mathcal O}_\mathrm{NLO} F^\mathrm{fin}_{LV}(1,2,4)\big \rangle ,\nonumber \\ \end{aligned}$$

where

9.5 $$\begin{aligned} \tilde{\mathcal D}_i(z) = \mathcal D_i(z) - \frac{1+z}{2} \ln ^i(1-z). \end{aligned}$$

### Terms involving $F^\mathrm{fin}_{LV}(1,2)$ and $F^\mathrm{fin}_{LVV}(1,2)$

Next, we collect the finite remainders of the one-loop and two-loop virtual contributions to the $q \bar{q} \rightarrow V$ process. These contributions appear in the real-virtual, the double-virtual, the collinear subtraction and the ultraviolet renormalization. Upon combining them and expanding the resulting contributions in $\epsilon$, we obtain

9.6 $$\begin{aligned} \mathrm{d}\hat{\sigma }^\mathrm{NNLO}_{F^\mathrm{fin}_{LV}(1,2)}&= \frac{\alpha _\mathrm{s}(\mu )}{2\pi }\Bigg \{\frac{2\pi ^2}{3}C_\mathrm{F}\big \langle F^\mathrm{fin}_{LV}(1,2)\big \rangle \nonumber \\&\quad + \big \langle F^\mathrm{fin}_{LV^2}(1,2)\big \rangle + \big \langle F^\mathrm{fin}_{LVV}(1,2)\big \rangle \nonumber \\&\quad + \int \limits _0^1 \mathrm{d}z \left[ 4C_\mathrm{F}\tilde{\mathcal D}_1(z) - \ln \left( \frac{\mu ^2}{s}\right) \hat{P}^{(0)}_{qq}(z) -P^{(\epsilon )}_{qq}(z)\right] \nonumber \\&\quad \times \bigg \langle \frac{F^\mathrm{fin}_{LV}(z\cdot 1,2)+F^\mathrm{fin}_{LV}(1,z\cdot 2)}{z} \bigg \rangle \Bigg \} . \end{aligned}$$

### Terms of the form $\mathcal P_1 \otimes \mathrm{d}\sigma \otimes \mathcal P_2$

Terms of the type $\mathcal P_1 \otimes \mathrm{d}\sigma \otimes \mathcal P_2$, where $\mathcal P_{1,2}$ are some splitting functions, appear in the double-real contribution as well as in the collinear renormalization. Combining all the relevant terms, we find

9.7 $$\begin{aligned} \mathrm{d}\hat{\sigma }^\mathrm{NNLO}_{F_{LM}(z \cdot 1,\bar{z} \cdot 2)}&= \left( \frac{\alpha _\mathrm{s}(\mu )}{2\pi }\right) ^2 C_\mathrm{F}^2 \int \limits _{0}^{1} \mathrm{d}z\; \mathrm{d}\bar{z} \left[ 2\tilde{\mathcal D}_0(z) \ln \left( \frac{\mu ^2}{s}\right) \right. \nonumber \\&\quad \left. - 4\tilde{\mathcal D}_1(z) -(1-z)\right] \nonumber \\&\quad \times \Bigg \langle \frac{F_{LM}(z\cdot 1,\bar{z}\cdot 1)}{z\bar{z}} \Bigg \rangle \left[ 2\tilde{\mathcal D}_0(\bar{z}) \ln \left( \frac{\mu ^2}{s}\right) \right. \nonumber \\&\quad \left. - 4\tilde{\mathcal D}_1(\bar{z}) -(1-\bar{z}) \right] . \end{aligned}$$

### Terms of the form $\mathcal P \otimes \mathrm{d}\sigma$

These terms appear in the double-real, real-virtual, collinear subtraction and ultraviolet renormalization contributions. We note that starting from $\mathcal{O}(1/\epsilon )$, the part of the double-real contribution related to the integral of the triple-collinear splitting function is only known numerically; see the discussion in Sect. 8.7.

Combining all the terms, we observe analytic cancellation of the poles up to $1/\epsilon ^2$. For the $1/\epsilon$ poles and the finite part, it is useful to split the contribution into a scale-independent and a scale-dependent term

9.8 $$\begin{aligned} \mathrm{d}\hat{\sigma }^\mathrm{NNLO}_{F_{LM}(z \cdot 1,2)} \equiv \mathrm{d}\hat{\sigma }^\mathrm{NNLO}_{F_{LM}(z \cdot 1,2)}(\mu ^2=s)+ \Delta ^\mathrm{NNLO}_{F_{LM}(z\cdot 1,2)}(\mu ). \end{aligned}$$

We also introduce an expansion of the functions $R^{(k)}(z)$ and the constants $R^{(k)}_+$, which were introduced in Section 8.7, in powers of $\epsilon$

9.9 $$\begin{aligned} \sum \limits _{k\in \mathrm{sectors}} R^{(k)}(z)= & {} R^{(0)}(z) + \epsilon R^{(\epsilon )}(z)+ \mathcal{O}(\epsilon ^2),\nonumber \\ \sum \limits _{k\in \mathrm{sectors}} R_+^{(k)}= & {} R_+^{(0)} + \epsilon R_+^{(\epsilon )} + \mathcal{O}(\epsilon ^2). \end{aligned}$$

The scale-independent term reads

9.10 $$\begin{aligned}&\mathrm{d}\hat{\sigma }^\mathrm{NNLO}_{F_{LM}(z \cdot 1,2)}(\mu ^2=s) = \left[ \frac{\alpha _\mathrm{s}(\mu )}{2\pi }\right] ^2 \nonumber \\&\quad \times \int \limits _0^1 \mathrm{d}z \Bigg \{ C_\mathrm{F}^2\Bigg [ 8\tilde{\mathcal D}_3(z) + 4\tilde{\mathcal D}_1(z)(1+\ln 2)\nonumber \\&\quad +4\tilde{\mathcal D}_0(z)\bigg [\frac{\pi ^2}{3}\ln 2 +4\zeta _3\bigg ] \nonumber \\&\quad +\frac{5z-7}{2} + \frac{5-11z}{2}\ln z + (1-3z)\ln 2 \ln z\nonumber \\&\quad +\ln (1-z)\bigg [\frac{3}{2}z-(5+11z)\ln z\bigg ] \nonumber \\&\quad +2(1-3z)\text {Li}_2(1-z) \nonumber \\&\quad +(1-z)\bigg [\frac{4}{3}\pi ^2+\frac{7}{2}\ln ^2 2-2\ln ^2(1-z)\nonumber \\&\quad +\ln 2\big [4\ln (1-z)-6\big ]+\ln ^2 z \nonumber \\&\quad +\text {Li}_2(1-z)\bigg ] +(1+z)\bigg [-\frac{\pi ^2}{3}\ln z - \frac{7}{4}\ln ^2 2 \ln z\nonumber \\&\quad -2\ln 2 \ln (1-z)\ln z +4 \ln ^2(1-z)\ln z\nonumber \\&\quad -\frac{\ln ^3 z}{3}+\big [4\ln (1-z)-2\ln 2\big ]\text {Li}_2(1-z) \bigg ] \nonumber \\&\quad +\left[ \frac{1+z^2}{1-z}\right] \ln (1-z) \big [3\text {Li}_2(1-z)-2\ln ^2 z\big ]\nonumber \\&\quad - \frac{5-3z^2}{1-z}\text {Li}_3(1-z) +\frac{\ln z}{(1-z)}\nonumber \\&\quad \times \bigg [ 12 \ln (1-z)-\frac{3-5z^2}{2}\ln ^2(1-z)-\frac{7+z^2}{2}\ln 2 \ln z\bigg ] \Bigg ] \nonumber \\&\quad +C_\mathrm{A}C_\mathrm{F}\Bigg [ -\frac{22}{3}\tilde{\mathcal D}_2(z)+\left( \frac{134}{9}-\frac{2}{3}\pi ^2\right) \tilde{\mathcal D}_1(z)\nonumber \\&\quad + \bigg [-\frac{802}{27}+\frac{11}{18}\pi ^2 +(2\pi ^2-1)\frac{\ln 2}{3} +11\ln ^2 2 + 16 \zeta _3\bigg ]\tilde{\mathcal D}_0(z)\nonumber \\&\quad +\frac{37-28z}{9}+ \frac{1-4z}{3}\ln 2 -\left( \frac{61}{9}+\frac{161}{18}z\right) \ln (1-z)\nonumber \\&\quad + (1+z)\ln (1-z)\bigg [\frac{\pi ^2}{3}-\frac{22}{3}\ln 2\bigg ] \nonumber \\&\quad -(1-z)\bigg [\frac{\pi ^2}{6}+\text {Li}_2(1-z)\bigg ] -\frac{2+11z^2}{3(1-z)}\ln 2\ln z\nonumber \\&\quad -\frac{1+z^2}{1-z}\text {Li}_2(1-z)\times \times \big [2\ln 2+3\ln (1-z)\big ]\Bigg ]\nonumber \\&\quad +R^{(\epsilon )}_{+} \mathcal D_0(z) + R^{(\epsilon )}(z) \Bigg \}\Bigg \langle \frac{F_{LM}(z\cdot 1,2)}{z} \Bigg \rangle . \end{aligned}$$

The scale-dependent term reads

9.11 $$\begin{aligned}&\Delta ^\mathrm{NNLO}_{F_{LM}(z \cdot 1,2)}(\mu ) = \left[ \frac{\alpha _\mathrm{s}(\mu )}{2\pi }\right] ^2\nonumber \\&\quad \times \int \limits _0^1 \mathrm{d}z \Bigg \{ C_\mathrm{F}^2\Bigg [ -12\tilde{\mathcal D}_1(z)-12\tilde{\mathcal D}_2(z) -6+5z \nonumber \\&\quad +2 (1-z)\ln (1-z) -2\ln z \frac{1+z+z^2}{1-z} \nonumber \\&\quad -(1+z)\bigg [2\ln z\ln (1-z)+ \frac{\ln ^2 z}{2}+2\text {Li}_2(1-z)\bigg ] \nonumber \\&\quad +2\frac{1+z^2}{1-z}\big [\ln (1-z)\ln z-\text {Li}_2(1-z)\big ]\Bigg ] \nonumber \\&\quad +C_\mathrm{A}C_\mathrm{F}\Bigg [ \frac{22}{3}\tilde{\mathcal D}_1(z)-\left( \frac{67}{9}-\frac{\pi ^2}{3}\right) \tilde{\mathcal D}_0(z) \nonumber \\&\quad -\frac{5-8z}{6} - \frac{2+11z^2}{6(1-z)}\ln z +\frac{1+z^2}{1-z}\text {Li}_2(1-z)\Bigg ]\Bigg \}\nonumber \\&\quad \times \Bigg \langle \frac{F_{LM}(z\cdot 1,2)}{z}\Bigg \rangle \times \ln \left( \frac{\mu ^2}{s}\right) +\left( \frac{\alpha _\mathrm{s}(\mu )}{2\pi }\right) ^2 \nonumber \\&\quad \times \int \limits _0^1 dz \Bigg \{ C_\mathrm{F}^2\bigg [ 4\tilde{\mathcal D}_1(z)+6\tilde{\mathcal D}_0(z)-(1-z)-\frac{1+3z^2}{2(1-z)}\ln z\bigg ] \nonumber \\&\quad -\frac{11}{6}C_\mathrm{A}C_\mathrm{F}\tilde{\mathcal D}_0(z)\Bigg \} \Bigg \langle \frac{F_{LM}(z\cdot 1,2)}{z}\Bigg \rangle \times \ln ^2\left( \frac{\mu ^2}{s}\right) . \end{aligned}$$

To arrive at these results, we check the cancellation of $1/\epsilon$ poles in $\mathrm{d}\hat{\sigma }^\mathrm{NNLO}_{F_{LM}(z \cdot 1,2)}$ and then, assuming that the cancellation is exact, deduce the analytic form of $R^{(0)}(z)$ and $R_+^{(0)}$. These analytic results are then used in the scale-dependent term. Thus the only numerical contributions needed for the finite part are $R^{(\epsilon )}(z)$ and $R_+^{(\epsilon )}$.

### Terms involving $F_{LM}(1,2)$

All the different contributions to the NNLO cross section produce terms proportional to $F_{LM}(1,2)$. These include constants $R_\delta ^{(k)}$ originating from the triple-collinear splitting function, which, as mentioned in the previous subsection, are only known numerically. As before, we introduce an expansion in $\epsilon$ for these constants.

9.12 $$\begin{aligned} \sum \limits _{k\in \mathrm{sectors}} R_\delta ^{(k)} = R_\delta ^{(0)} + \epsilon R_\delta ^{(\epsilon )} + \mathcal{O}(\epsilon ^2). \end{aligned}$$

Furthermore, for the double-soft contribution we know the abelian constants of Eq. (8.21) analytically, but only have numerical results for the non-abelian constants, which are reported in Table 1. Thus, for each order in $1/\epsilon$, we check the cancellation of terms numerically and then, assuming that the cancellation is actually exact, we deduce an analytic form of the triple-collinear splitting and double-soft constants at this order. This form is then used in determining the cancellation at lower orders in $1/\epsilon$. Thus, the only numerical constants appearing in our final formula are $R_\delta ^{(\epsilon )}$ and $c^{SS}_{0,C_A C_\mathrm{F}}$. The final formula reads

9.13 $$\begin{aligned}&\mathrm{d}\hat{\sigma }^\mathrm{NNLO}_{F_{LM}(1,2)} = \left( \frac{\alpha _\mathrm{s}(\mu )}{2\pi }\right) ^2\Bigg [ 2R^{(\epsilon )}_{\delta } + C_\mathrm{F}^2\bigg ( \frac{8}{45}\pi ^4+\frac{7}{3}\pi ^2\ln ^2 2\nonumber \\&\quad - 16\zeta _3\ln 2\bigg )+C_\mathrm{A}C_\mathrm{F}\bigg ( \frac{4214}{81} +\frac{403}{72}\pi ^2 -\frac{17}{48}\pi ^4 -\frac{671}{36} \zeta _3 \nonumber \\&\quad + c^{SS}_{0,C_\mathrm{A}C_\mathrm{F}}-\frac{445}{54}\ln 2 - \frac{22}{9}\pi ^2 \ln 2 -\frac{149}{18}\ln ^2 2 + 4\pi ^2 \ln ^2 2\nonumber \\&\quad + \frac{22}{9}\ln ^3 2 - \frac{16}{3}\ln ^4 2-34\zeta _3\ln 2 \bigg ) \Bigg ]\big \langle F_{LM}(1,2)\big \rangle \nonumber \\&\quad +\left( \frac{\alpha _\mathrm{s}(\mu )}{2\pi }\right) ^2 \Bigg \{ C_\mathrm{F}^2\bigg [\left( \frac{9}{2}-\frac{2}{3}\pi ^2\right) \ln \left( \frac{\mu ^2}{s}\right) \nonumber \\&\quad -\left( \frac{3}{4}+\pi ^2+28\zeta _3\right) \bigg ] \nonumber \\&\quad +C_\mathrm{A}C_\mathrm{F}\bigg [-\frac{11}{4} \ln \left( \frac{\mu ^2}{s}\right) +\left( -\frac{17}{12}+6\zeta _3\right) \bigg ]\Bigg \}\nonumber \\&\quad \times \ln \left( \frac{\mu ^2}{s} \right) \big \langle F_{LM}(1,2)\big \rangle . \end{aligned}$$

## Numerical results

Having described the subtraction procedure in some detail, we will now study how well it works in practice. We have implemented it in a partonic Monte Carlo program to compute NNLO QCD corrections to the production of a vector boson $\gamma ^*$ in proton–proton collisions.^11^ The calculation is fully differential; we consider decays of the virtual photons to massless leptons and study NNLO QCD corrections to lepton observables. We extracted the relevant matrix elements from Refs. [81, 82] as implemented in [83], and from Refs. [84, 85]. For all computations reported below we employ the NNLO parton distribution functions from the NNPDF3.0 set [86].

We begin by comparing the analytic result for the NNLO QCD correction to the $pp \rightarrow \gamma ^* \rightarrow e^+e^- + X$ cross section, which we extract from Ref. [87], and the result of the numerical computation based on the formulas reported in the previous section. We wish to emphasize that this comparison is performed using the NNLO *contribution* to the cross section, and not the full cross section at NNLO, which would have included LO and NLO contributions as well. We take $14~\mathrm{TeV}$ as the center-of-mass collision energy. We include lepton pairs with invariant masses *Q* in the range $50~\mathrm{GeV}< Q < 350~\mathrm{GeV}$ and take $\mu = 100~\mathrm{GeV}$ for the renormalization and factorization scales. We obtain the NNLO *corrections* to the cross sections

**Fig. 2:**
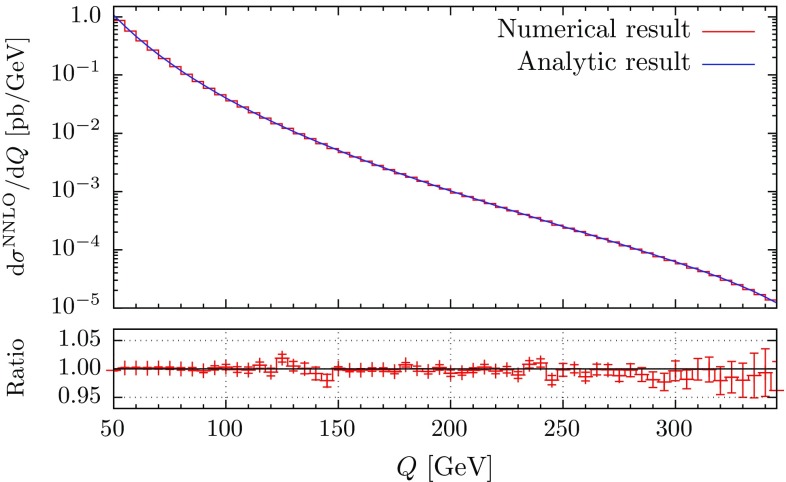
Comparison of the NNLO QCD contribution $\mathrm{d}\sigma ^\mathrm{NNLO}/\mathrm{d}Q$ computed in this paper with the analytic results in Ref. [87]

10.1 $$\begin{aligned} \mathrm{d} \sigma ^\mathrm{NNLO} = 14.471(4)~\mathrm{pb},\quad \mathrm{d} \sigma _\mathrm{analytic}^\mathrm{NNLO} = 14.470~\mathrm{pb}, \end{aligned}$$

where the first result is ours and the second is extracted from Ref. [87]. The agreement between the two results is quite impressive; it is significantly better than a permille. To further illustrate the degree of agreement, we repeat the comparison using the kinematic distribution $\mathrm{d} \sigma ^\mathrm{NNLO}/\mathrm{d}Q$, shown in Fig. 2. In the upper pane of Fig. 2, we see a perfect agreement of analytic and numerical results for a range of *Q*-values where the cross section changes by five orders of magnitude.

**Fig. 3:**
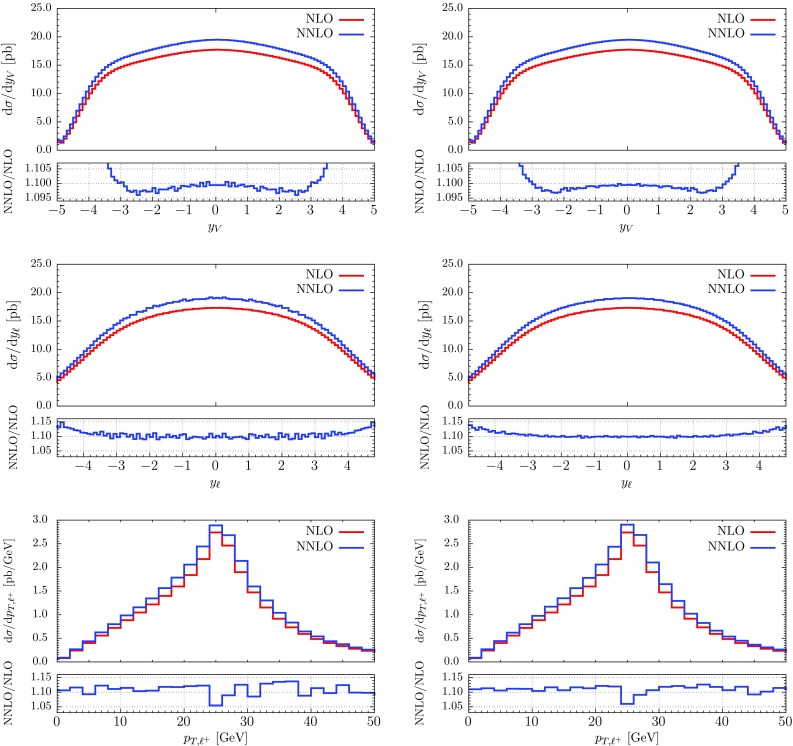
*Upper panes* Rapidity distribution of the vector boson, rapidity distribution of a lepton and $p_T$ distribution of a lepton at different orders of perturbation theory. *Lower panes* the ratio of NNLO/NLO prediction for a given observable. *Plots on the left* the runtime of $\mathcal{O}(10)$ CPU hours; *plots on the right* the runtime of $\mathcal{O}(100)$ CPU hours. Note that the dip in the ratio of NNLO/NLO lepton $p_T$ distribution at $p_T\sim 25~\mathrm{GeV}$ is a physical feature and *not* a fluctuation

The ratio of numerical and analytic cross sections is shown in the lower pane of Fig. 2. We see that the agreement is between a fraction of permille and a few percent for all values of *Q* considered. We reiterate that we plot the NNLO correction to the differential cross section and not the full cross section at NNLO. Given that the NNLO contribution changes the NLO result by about 10%, the permille to percent precision on the NNLO *correction* leads to almost absolute precision for physical *cross sections* and *simple kinematic distributions*. We will further illustrate this point below. Before doing so, we note that we found a similar level of agreement for individual color structures and for individual contributions to the final result. We also note that although we report results for a single scale choice here, using the results in the previous sections and the known amplitudes for $q\bar{q} \rightarrow e^+e^- + X$, it is easy to check *analytically* the scale dependence of our result against the one reported in Ref. [87]. Full agreement is found.

As we mentioned in the Introduction, one of the important issues for current NNLO QCD computations is their practicality. For example, with the increasing precision of Drell–Yan measurements, one may require very accurate theoretical predictions for fiducial volume cross sections. It is then important to clarify whether a given implementation of the NNLO QCD corrections can produce results that satisfy advanced stability requirements and, if so, how much CPU time is needed to achieve them.

To illustrate this aspect of our computational scheme, we show the rapidity distribution of the dilepton pair, the rapidity distribution of a lepton, and the lepton transverse momentum distribution in Fig. 3. The plots on the left and on the right provide identical information: the upper panes show next-to-leading and next-to-next-to-leading order predictions for the respective observable, and the lower panes the ratio of the NNLO to NLO distributions. The difference between the plots on the left and the plots on the right is the CPU time required to obtain them; it changes from $\mathcal {O}(10)$ CPU hours for the plots on the left, to $\mathcal {O}(100)$ CPU hours for the plots on the right. The different run times are reflected in different bin-to-bin fluctuations seen in both plots. The bin-to-bin fluctuations for the two rapidity distributions are at the percent-level or better in the plots on the left, and they become practically unobservable in the plots on the right. The situation is slightly worse for the transverse momentum of the lepton. However, this observable is rather delicate in the $\gamma ^*$ case, as each bin receives contributions from a large range of invariant masses. The introduction of a *Z* boson propagator will localize the bulk of the cross section in a much smaller invariant mass window, and lead to improved stability in this case.^12^ Nevertheless, the results shown in Fig. 3 imply that the numerical implementation of our subtraction scheme allows for high precision computations, while also delivering results that are acceptable for phenomenology even after relatively short run times.

## Conclusions

In this paper we described a modification of the residue-improved subtraction scheme [10, 11] that allows us to remove one of the five sectors that are traditionally used to fully factorize singularities of the double-real emission matrix elements squared. The redundant sector includes correlated soft-collinear limits where energies of emitted gluons and their angles vanish in a correlated fashion. Once this sector is removed, the physical picture of independent soft and collinear emissions leading to singularities in scattering amplitudes is recovered and the bookkeeping simplifies considerably.

Using these simplifications, we reformulated a NNLO subtraction scheme, based on nested subtractions of soft and collinear singularities that, in a straightforward way, leads to an integrable remainder for the double-real emission cross section. The subtraction terms are related to cross sections of reduced multiplicity; they can be rewritten in a way that allows us to prove the cancellation of $1/\epsilon$ singularities independent of the hard matrix elements. Once singularities cancel, the NNLO QCD corrected cross section is written in terms of quantities that can be computed in four dimensions.

Although we believe that this framework is applicable for generic NNLO QCD computations, in this paper, for the sake of simplicity, we studied dilepton pair production in quark–antiquark annihilation and computed gluonic contributions to NNLO corrections. We implemented our formulas in a numerical program and used it to calculate NNLO QCD *corrections* to the production cross section of a vector boson in hadron collisions with a sub-percent precision. We also showed that kinematic distributions, including the lepton rapidity and transverse momentum distributions, can be computed precisely and efficiently. We look forward to the application of the computational framework discussed in this paper to more complex processes, relevant for the LHC phenomenology.

## Appendix A: Generalized splitting functions

In this appendix, we summarize all the various splitting functions that appear in our calculation.

We start by presenting the Altarelli–Parisi splitting functions that are relevant for the calculation. At NLO, we only require the leading-order splitting function

A.1 $$\begin{aligned} \hat{P}^{(0)}_{qq}(z) = C_\mathrm{F}\left[ 2\mathcal D_0(z)-(1+z)+\frac{3}{2}\delta (1-z)\right] . \end{aligned}$$

For NNLO computations, two more splitting functions are needed; see Eqs. (2.10) and (2.11). The first one is the convolution of two leading-order splitting functions. It reads

A.2 $$\begin{aligned}&\left[ \hat{P}^{(0)}_{qq}\otimes \hat{P}^{(0)}_{qq}\right] (z) \nonumber \\&\quad =C_\mathrm{F}^2\bigg [ 8 \mathcal D_1(z)+6 \mathcal D_0(z)+ \left( \frac{9}{4}-\frac{2}{3}\pi ^2\right) \delta (1-z) \nonumber \\&\qquad -\,\frac{(1+3z^2)}{1-z}\ln z- 4(1+z)\ln (1-z) -5 - z\bigg ]. \end{aligned}$$

The second is the splitting function $\hat{P}_{qq}^{(1)}$. We emphasize that, for our case, we need the NLO splitting function for a continuous quark line entering the hard matrix element after the emission of two gluons. We then only have to consider the non-singlet NLO splitting function from which the contribution of identical quarks is subtracted. We extract the relevant information from Ref. [87]. $\hat{P}_{qq}^{(1)}$ in Eq. (2.11) must then be identified with

A.3 $$\begin{aligned}&\hat{P}^{(1)}_{qq,\widetilde{\mathrm{NS}}}(x) = C_\mathrm{A}C_\mathrm{F}\Bigg [ \frac{3\pi ^2(1+x)-124 x -19}{18}\nonumber \\&\quad +\left( \frac{67}{9}-\frac{\pi ^2}{3}\right) \mathcal D_0(x) +\frac{2+11x^2}{6(1-x)}\ln x \nonumber \\&\quad -\frac{1+x^2}{1-x}\text {Li}_2(1-x)+\delta (1-x)\left( \frac{17}{24}+\frac{11}{18}\pi ^2-3\zeta _3\right) \Bigg ] \nonumber \\&\quad +C_\mathrm{F}^2\Bigg [3-2x -2\frac{1+x^2}{1-x}\ln (1-x)\ln x\nonumber \\&\quad +2\ln x + \frac{1+3x^2}{2(1-x)}\ln ^2 x+2\frac{1+x^2}{1-x}\text {Li}_2(1-x)\nonumber \\&\quad +\delta (1-x)\left( \frac{3}{8}-\frac{\pi ^2}{2}+6\zeta _3\right) \Bigg ]. \end{aligned}$$

We now list the definitions of the various splitting functions used in the text. In these formulas, we use the definition

A.4 $$\begin{aligned} L_1 = \ln \left( 2\frac{E_\mathrm{max}}{\sqrt{s}}\right) . \end{aligned}$$

- The tree-level splitting function used in the NLO computation is defined as
  A.5 $$\begin{aligned}&\mathcal P_{qq,R}(z) = C_\mathrm{F}\Bigg \{\bigg [ \frac{3}{2} \delta (1-z) + 2\mathcal D_0(z) \nonumber \\&\quad - 4 \mathcal D_1(z)\epsilon + 4\mathcal D_2(z)\epsilon ^2\bigg ] \nonumber \\&\quad -(1+z) + \bigg [2(1+z)\ln (1-z) -(1-z)\bigg ]\epsilon \nonumber \\&\quad +\bigg [ 2 (1-z) \ln (1-z)-2 (1+z) \ln ^2(1-z) \bigg ]\epsilon ^2 \Bigg \} + \mathcal O(\epsilon ^3). \end{aligned}$$
- The splitting function $\mathcal P_{qq,\mathrm{NLO_{CV}}}(z)$ reads
  A.6 $$\begin{aligned}&\mathcal P_{qq,\mathrm{NLO_{CV}}}(z) \nonumber \\&\quad = C_\mathrm{F}\hat{P}^{(0)}_{qq} \left[ \frac{2\pi ^2}{3} + \epsilon (\pi ^2-4\zeta _3)\right] \nonumber \\&\qquad +\,C_\mathrm{F}^2\frac{1+z^2}{1-z} \ln z \left[ \ln z- \epsilon \left( \frac{2 \pi ^2}{3} - \frac{3}{2}\ln z + \ln ^2 z\right) \right] \nonumber \\&\qquad +\,\mathcal O(\epsilon ^2). \end{aligned}$$
- The splitting function $\big [\mathcal P_{qq}\otimes \mathcal P_{qq}\big ]_\mathrm{NLO_{CV}}(z)$ reads
  A.7 $$\begin{aligned}&\big [\mathcal P_{qq}\otimes \mathcal P_{qq}\big ]_\mathrm{NLO_{CV}}(z) =C_\mathrm{F}^2\Bigg \{ \bigg [6\mathcal D_0(z)+8\mathcal D_1(z)\nonumber \\&\quad +\left( \frac{9}{4}-\frac{2}{3}\pi ^2\right) \delta (1-z)\bigg ] \nonumber \\&\quad +\bigg [\frac{4}{3}\pi ^2 \mathcal D_0(z)-6\mathcal D_1(z)-12\mathcal D_2(z)-8\zeta _3\delta (1-z)\bigg ]\epsilon \nonumber \\&\quad +\bigg [16\zeta _3\mathcal D_0(z)-\frac{8}{3}\pi ^2\mathcal D_1(z)+6\mathcal D_2(z)+\frac{32}{3}\mathcal D_3(z)\nonumber \\&\quad -\frac{8}{45}\pi ^4\delta (1-z)\bigg ]\epsilon ^2 \nonumber \\&\quad +\bigg [-5-z-4(1+z)\ln (1-z) - \frac{(1+3z^2)\ln z}{1-z}\bigg ] \nonumber \\&\quad +\bigg [-\frac{3}{2}(1-z)+(5+z)\ln (1-z)+2(3+z)\ln z\nonumber \\&\quad +\frac{4\ln ^2 z-6\ln z}{1-z}+(1+z) \nonumber \\&\quad \times \big [2\text {Li}_2(z)-3\ln ^2 z+6\ln ^2 (1-z)-\pi ^2\big ]\bigg ]\epsilon \nonumber \\&\quad +\bigg [\pi ^2\left( z-\frac{5}{3}\right) + (1-z)\big [3\ln (1-z)-2\big ]\nonumber \\&\quad -2\ln z -2(3+z)\ln ^2 z \nonumber \\&\quad +\frac{\ln ^2 z}{1-z}\left( 6-\frac{8}{3}\ln z\right) + (6-2z)\text {Li}_2(z)\nonumber \\&\quad + (1+z)\bigg (-\frac{16}{3}\ln ^3(1-z)-\ln ^2(1-z)\big [3+2\ln z\big ] \nonumber \\&\quad +\frac{2}{3}\pi ^2\ln z + 2 \ln ^3 z +\ln (1-z) \nonumber \\&\quad \times \big [2\pi ^2-4\text {Li}_2(z)\big ] -4\text {Li}_3(z)-4\text {Li}_3(1-z)-4\zeta _3\bigg ) \bigg ]\epsilon ^2\Bigg \}\nonumber \\&\quad + \mathcal O(\epsilon ^3). \end{aligned}$$
- The one-loop splitting functions used in the computation of the real-virtual limits reads
  A.8 $$\begin{aligned}&P_{qq}^\mathrm{loop,i}(z) = -(1-z) P_{qq}(z)\nonumber \\&\quad \times \Bigg \{ \bigg [\frac{1}{\epsilon ^2}\frac{\Gamma ^2(1-\epsilon )\Gamma ^2(1+\epsilon )}{\Gamma (1-2\epsilon )\Gamma (1+2\epsilon )} \frac{1}{(1-z)^{2\epsilon }}+2\text {Li}_2(1-z) \nonumber \\&\quad -2\epsilon \text {Li}_2(1-z)\ln (1-z)\bigg ]C_\mathrm{A}\nonumber \\&\quad +2\bigg [\frac{\ln z}{\epsilon } -\text {Li}_2(1-z)-\ln z\ln (1-z)+\bigg ( \frac{1}{2}\ln ^2(1-z)\ln z \nonumber \\&\quad + \ln (1-z) \text {Li}_2(1-z) - \text {Li}_3(1-z) \bigg )\epsilon \bigg ]C_\mathrm{F}\Bigg \}\nonumber \\&\quad +(C_\mathrm{A}-C_\mathrm{F}) \mathcal P^\mathrm{RV,new}_{qq}(z) +\mathcal O(\epsilon ^2), \end{aligned}$$
  where
  A.9 $$\begin{aligned} \mathcal P_{qq}^\mathrm{RV,new}(z)&= -C_\mathrm{F}(1-z)\bigg [z + (1+z-z \ln (1-z))\epsilon \bigg ]\nonumber \\&\quad + \mathcal O (\epsilon ^2). \end{aligned}$$
- The tree-level splitting function used in the real-virtual contribution is defined as
  A.10 $$\begin{aligned}&\mathcal P_{qq,RV,1}(z) = C_\mathrm{F}\Bigg \{ 2\big [\delta (1-z)L_{1}-\mathcal D_0(z)\big ]\nonumber \\&\quad +2\big [2\mathcal D_1(z)-\delta (1-z)L_{1}^2\big ]\epsilon +\left[ \frac{4}{3}\delta (1-z)L_{1}^3-4\mathcal D_2(z)\right] \epsilon ^2\nonumber \\&\quad + \left[ -\frac{2}{3}\delta (1-z)L_{1}^4 + \frac{8}{3}\mathcal D_3(z)\right] \epsilon ^3 +(1+z) \nonumber \\&\quad + \big [(1-z) - 2(1+z) \ln (1-z)\big ]\epsilon \nonumber \\&\quad +2\bigg [(1+z)\ln ^2(1-z) -(1-z)\ln (1-z)\bigg ]\epsilon ^2 \nonumber \\&\quad +\Bigg [ 2(1-z)\ln ^2(1-z)-\frac{4}{3}(1+z) \ln ^3(1-z) \Bigg ]\epsilon ^3\Bigg \} + \mathcal O(\epsilon ^4). \end{aligned}$$
- The one-loop splitting function used in the real-virtual contribution reads
  A.11 $$\begin{aligned}&\mathcal P_{RV,2}(z) = -C_\mathrm{A}C_\mathrm{F}\Bigg \{ \frac{1}{\epsilon ^2}\left[ \delta (1-z)L_{1} - \mathcal D_0(z)\right] \nonumber \\&\quad + \frac{2}{\epsilon }\left[ 2\mathcal D_1(z)-\delta (1-z)L_{1}^2\right] \nonumber \\&\quad + \left[ -\frac{1}{3}\delta (1-z)L_{1}\left( \pi ^2-8L_{1}^2\right) +\frac{\pi ^2}{3} \mathcal D_0(z) - 8 \mathcal D_2(z) \right] \nonumber \\&\quad + \bigg [\frac{2}{3}\delta (1-z)L_{1}^2 \times \left( \pi ^2-4L_{1}^2\right) \nonumber \\&\quad -\frac{4}{3}\pi ^2\mathcal D_1(z)+\frac{32}{3}\mathcal D_3(z)\bigg ]\epsilon +\frac{1+z}{2\epsilon ^2} \nonumber \\&\quad +\frac{1}{2\epsilon }\bigg [(1-z)-4(1+z)\times \ln (1-z)\bigg ] \nonumber \\&\quad +\bigg [ -\frac{z}{2}-2(1-z)\ln (1-z) \nonumber \\&\quad + (1+z)\bigg ( 4\ln ^2(1-z)-\frac{\pi ^2}{6}\bigg ) -\frac{(1+z^2)\text {Li}_2(1-z)}{(1-z)}\bigg ] \nonumber \\&\quad +\bigg [ \frac{3}{2} z\ln (1-z) + \frac{1+z}{6}\big [ -3 + 4 \pi ^2\ln (1-z) -32\ln ^3(1-z)\big ] \nonumber \\&\quad +(1-z)\bigg (4\ln ^2(1-z)-\frac{\pi ^2}{6} + \text {Li}_2(1-z)\bigg ) \nonumber \\&\quad +3\left[ \frac{1+z^2}{1-z}\right] \times \ln (1-z)\text {Li}_2(1-z) \bigg ]\epsilon \Bigg \} \nonumber \\&\quad -C_\mathrm{F}^2\Bigg \{ -\frac{1}{\epsilon }\left[ \frac{(1+z^2)\ln z}{1-z}\right] \nonumber \\&\quad +\bigg [ \frac{z}{2} + (1-z)\ln z+\frac{1+z^2}{1-z} \big [ 3 \ln (1-z)\ln z + \text {Li}_2(1-z)\big ] \bigg ]\nonumber \\&\quad + \bigg [ \frac{1}{2}\big [1+z-3z\ln (1-z)\big ] \nonumber \\&\quad -(1-z)\big [3\ln (1-z)\ln z + \text {Li}_2(1-z)\big ]\nonumber \\&\quad -\frac{1}{2}\left[ \frac{1+z^2}{(1-z)}\right] \big [9\ln ^2(1-z)\ln z \nonumber \\&\quad + 6 \ln (1-z)\text {Li}_2(1-z) -2\text {Li}_3(1-z)\big ] \bigg ]\epsilon \Bigg \} + \mathcal O(\epsilon ^2).\nonumber \\ \end{aligned}$$
- The splitting function $\mathcal P_{qq,RR_1}$ reads
  A.12 $$\begin{aligned}&\mathcal P_{qq,RR_1}(z) = C_\mathrm{F}\Bigg \{\bigg [ 2\big [\mathcal D_0(z)-L_{1}\delta (1-z) \big ] \nonumber \\&\quad -4\big [2\mathcal D_1(z) -L_{1}^2\delta (1-z)\big ]\epsilon \nonumber \\&\quad +16\big [\mathcal D_2(z) - \frac{1}{3}L_{1}^3\delta (1-z)\big ]\epsilon ^2 \nonumber \\&\quad -\frac{16}{3}\big [4 \mathcal D_3(z)- L_{1}^4\delta (1-z)\big ]\epsilon ^3 \bigg ]\nonumber \\&\quad +\bigg [-(1+z) + \big [4(1+z)\ln (1-z) -(1-z)\big ]\epsilon \nonumber \\&\quad +\big [4(1-z)\ln (1-z) - 8 (1+z) \ln ^2(1-z) \big ]\epsilon ^2 \nonumber \\&\quad +\left[ \frac{32}{3}(1+z)\ln ^3(1-z) - 8 (1-z)\ln ^2(1-z) \right] \epsilon ^3 \bigg ] \Bigg \} \nonumber \\&\quad +\mathcal O(\epsilon ^4). \end{aligned}$$
- The splitting function $\mathcal P_{qq,RR_2}(z)$ reads
  A.13 $$\begin{aligned}&\mathcal P_{qq,RR_2}(z) = C_\mathrm{F}\Bigg \{\bigg [ 2\mathcal D_0(z) - 4 \mathcal D_1(z)\epsilon + 4\mathcal D_2(z)\epsilon ^2\nonumber \\&\quad -\frac{8}{3}\mathcal D_3(z)\epsilon ^3\bigg ] \nonumber \\&\quad +\bigg [ -(1+z) + \big [2(1+z)\ln (1-z) -(1-z)\big ]\epsilon \nonumber \\&\quad +\big [ 2 (1-z) \ln (1-z) -2 (1+z) \ln ^2(1-z) \big ]\epsilon ^2 \nonumber \\&\quad +\left[ -2(1-z)\ln ^2(1-z) +\frac{4}{3}(1+z)\ln ^3(1-z) \right] \epsilon ^3 \bigg ] \Bigg \}\nonumber \\&\quad + \mathcal O(\epsilon ^3). \end{aligned}$$
- The splitting function $\mathcal P_{qq,RR_3}(z)$ reads
  A.14 $$\begin{aligned}&\mathcal P_{qq,RR_3}(z) = C_\mathrm{F}\Bigg \{ \left[ 2L_{1} \mathcal D_0(z) - 2\mathcal D_1(z) - \delta (1-z)L_{1}^2\right] \nonumber \\&\quad + \left[ 6\mathcal D_2(z) - 4 L_{1} \mathcal D_1(z) - 2 L_{1}^2 \mathcal D_0(z) + 2 \delta (1-z)L_{1}^3\right] \epsilon \nonumber \\&\quad +\left[ -\frac{28}{3}\mathcal D_3(z) + 4L_{1} \mathcal D_2(z) + 4 L_{1}^2 \mathcal D_1(z) + \frac{4}{3}L_{1}^3 \mathcal D_0(z)\right. \nonumber \\&\quad \left. - \frac{7}{3}\delta (1-z) L_{1}^4 \right] \epsilon ^2 + \left[ (1+z)(\ln (1-z)-L_{1})\right] \nonumber \\&\quad + \bigg [ (1-z)(\ln (1-z)-L_{1}) \nonumber \\&\quad +(1+z) \left( L_{1}^2 + 2 L_{1} \ln (1-z) -3 \ln ^2(1-z) \right) \bigg ]\epsilon \nonumber \\&\quad + \bigg [ (1-z)\left( L_{1}^2 + 2 L_{1} \ln (1-z) - 3\ln ^2(1-z)\right) +(1+z)\nonumber \\&\quad \times \left( -\frac{2}{3}L_{1}^3 - 2 L_{1} \ln ^2(1-z) + \frac{14}{3}\ln ^3(1-z) -2\ln (1-z)L_{1}^2 \right) \bigg ]\epsilon ^2 \Bigg \}\nonumber \\&\quad + \mathcal O(\epsilon ^3). \end{aligned}$$
- The splitting function $\mathcal P_{qq,RR_4}(z)$ reads
  A.15 $$\begin{aligned}&\mathcal P_{qq,RR_4}(z)=C_\mathrm{F}\Bigg \{ \left[ \delta (1-z)L_{1}^2-2\mathcal D_1(z) \right] \nonumber \\&\quad + \left[ -2\delta (1-z)L_{1}^3 + 6\mathcal D_2(z) \right] \epsilon \nonumber \\&\quad +\left[ \frac{7}{3}\delta (1-z)L_{1}^4 - \frac{28}{3}\mathcal D_3(z) \right] \epsilon ^2 +\left[ (1+z)\ln (1-z)\right] \nonumber \\&\quad +\left[ (1-z)\ln (1-z) -3 (1+z) \ln ^2(1-z)\right] \epsilon \nonumber \\&\quad +\left[ \frac{14}{3}(1+z)\ln ^3(1-z) -3(1-z)\ln ^2(1-z) \right] \epsilon ^2\Bigg \}\nonumber \\&\quad + \mathcal O(\epsilon ^3). \end{aligned}$$
- The splitting function $\mathcal P_{qq,RR_5}(z)$ reads
  A.16 $$\begin{aligned}&\mathcal P_{qq,RR_5}(z) = C_\mathrm{F}\bigg [ 2\big [\mathcal D_0(z) - L_{1} \delta (1-z)\big ]\nonumber \\&\quad + \big [4L_{1}^2 \delta (1-z) -8 \mathcal D_1(z)\big ]\epsilon \nonumber \\&\quad -\frac{3+z}{2} + 2(3+z)\ln (1-z) \epsilon \bigg ] + \mathcal O(\epsilon ^2). \end{aligned}$$
- The splitting function $\mathcal P_{qq,RR_6}(z)$ reads
  A.17 $$\begin{aligned}&\mathcal P_{qq,RR_6}(z)=C_\mathrm{F}^2\Bigg ( 4\mathcal D_1(z) - 12\mathcal D_2(z)\epsilon + \frac{56}{3}\mathcal D_3(z)\epsilon ^2\nonumber \\&\quad -2(1+z)\ln (1-z)\nonumber \\&\quad +\left[ -2(1-z)\ln (1-z) +6 (1+z) \ln ^2(1-z)\right] \epsilon \nonumber \\&\quad +\left[ 6(1-z)\ln ^2(1-z)-\frac{28}{3}(1+z)\ln ^3(1-z)\right] \epsilon ^2 \Bigg )\nonumber \\&\quad +\mathcal O(\epsilon ^3). \end{aligned}$$
- The splitting function $\big [\mathcal P_{qq}\otimes \mathcal P_{qq}\big ]_{RR}(z)$ is defined as
  A.18 $$\begin{aligned}&\big [\mathcal P_{qq}\otimes \mathcal P_{qq}\big ]_{RR}(z) = C_\mathrm{F}^2 \Bigg \{ \bigg [ 8\mathcal D_1(z) -\frac{2}{3}\pi ^2 \delta (1-z) \nonumber \\&\quad - 4\mathcal D_0(z) L_{1} \bigg ] +\bigg [ \left( \frac{8}{3}\pi ^2 + 4 L_{1}^2\right) \mathcal D_0(z)\nonumber \\&\quad + 8 L_{1} \mathcal D_1(z)-24 \mathcal D_2(z)- 16 \zeta _3 \delta (1-z) \bigg ]\epsilon \nonumber \\&\quad + \bigg [ \left( -\frac{8}{3}L_{1}^3 + 64 \zeta _3\right) \mathcal D_0(z) -\left( 8 L_{1}^2 + \frac{32}{3} \pi ^2\right) \mathcal D_1(z)\nonumber \\&\quad - 8 L_{1} \mathcal D_2(z)+\frac{112}{3} \mathcal D_3(z) - \frac{2}{5}\pi ^4 \delta (1-z) \bigg ]\epsilon ^2\Bigg \}\nonumber \\&\quad +C_\mathrm{F}^2 \Bigg \{ \bigg [ -2(1-z)+ (1+z) \big ( \ln z - 4 \ln (1-z)+2L_{1} \big ) \bigg ] \nonumber \\&\quad + \bigg [ 2(1-z) \left( 2\ln (1-z)+L_{1}\right) + (1+z) \big [ 12\ln ^2(1-z) \nonumber \\&\quad +2\ln z - \ln ^2 z + 4 \text {Li}_2(z)-2\pi ^2 - 2 L_{1}^2 - 4 L_{1} \ln (1-z)\big ] \bigg ]\epsilon \nonumber \\&\quad + \bigg [ -2(1-z)\big (2\ln ^2(1-z)+2L_{1} \ln (1-z)+L_{1}^2 + 5\big )\nonumber \\&\quad +(1+z) \bigg ( 4\ln ^2(1-z)L_{1} +4 \ln (1-z)L_{1}^2 \nonumber \\&\quad +\frac{4}{3}L_{1}^3 -\frac{4}{3}\pi ^2 + 8\pi ^2 \ln (1-z)\nonumber \\&\quad - \frac{56}{3} \ln ^3(1-z) -5 \ln z+ \frac{2}{3}\pi ^2 \ln z \nonumber \\&\quad - 8 \ln ^2(1-z)\ln z - 2 \ln ^2 z + \frac{2}{3}\ln ^3 z+8 \text {Li}_2(z) \nonumber \\&\quad - 16 \ln (1-z) \text {Li}_2(z) -16 \text {Li}_3(1-z)\nonumber \\&\quad -8 \text {Li}_3(z) - 24 \zeta _3 \bigg ) \bigg ]\epsilon ^2\Bigg \} + \mathcal O (\epsilon ^3). \end{aligned}$$

We also require the following constants:

A.19 $$\begin{aligned} \tilde{\gamma }_g(\epsilon )&= \frac{11}{6}C_\mathrm{A}+ C_\mathrm{A}\left( \frac{137}{18}-\frac{2\pi ^2}{3}\right) \epsilon \nonumber \\&\quad + C_\mathrm{A}\left( \frac{823}{27}-\frac{11}{18}\pi ^2 - 16\zeta _3 \right) \epsilon ^2+ \mathcal O(\epsilon ^3),\nonumber \\ \tilde{\gamma }_g(\epsilon ,k_{\perp })&= -\frac{C_\mathrm{A}}{3} - \frac{7C_\mathrm{A}}{9}\epsilon + \mathcal O(\epsilon ^2), \end{aligned}$$

and

A.20 $$\begin{aligned} \delta _g(\epsilon )&= C_\mathrm{A}\left( -\frac{131}{72}+\frac{\pi ^2}{6} + \frac{11}{6}\ln 2\right) \nonumber \\&\quad +C_\mathrm{A}\left( -\frac{1541}{216}+\frac{11}{18}\pi ^2-\frac{\ln 2}{6}+4\zeta _3\right) \epsilon \nonumber \\&\quad +C_\mathrm{A}\left( -\frac{9607}{324}+\frac{125}{216}\pi ^2+\frac{7}{45}\pi ^4 +\ln 2\right. \nonumber \\&\quad \left. + \frac{11}{18}\pi ^2\ln 2 + \frac{77}{6}\zeta _3\right) \epsilon ^2+ \mathcal O(\epsilon ^3). \end{aligned}$$

## Appendix B: Phase space parametrization and partitioning

We consider the phase space element of the two gluons

B.1 $$\begin{aligned}{}[\mathrm{d}g_{4}] [\mathrm{d}g_{5}] \theta (E_\mathrm{max} - E_4) \theta ( E_4 - E_5) \end{aligned}$$

and discuss its parametrization.

We take $E_4 = E_\mathrm{max}\; x_1$, $E_5 = E_\mathrm{max}\; x_1 x_2$ with $x_1\in (0,1)$ and $x_2\in (0,1)$ and write the phase space in Eq. (B.1) as

B.2 $$\begin{aligned}{}[dg_4][dg_5]&= \frac{ \mathrm{d} x_1 }{x_1^{1+4\epsilon }} \frac{ \mathrm{d} x_2 }{x_2^{1+2\epsilon }} \; x_1^4 \; x_2^2 \; E_\mathrm{max}^{4 - 4 \epsilon } \; \mathrm{d \Omega }_{45}, \nonumber \\ \mathrm{d}\Omega _{45}&= \frac{\mathrm{d} \Omega _4^{(d-1)}}{ 2 ( 2\pi )^{d-1}} \frac{\mathrm{d} \Omega _5^{(d-1)}}{ 2 ( 2\pi )^{d-1}}. \end{aligned}$$

We will now introduce the parametrization of the angular phase space. This parametrization is tailored for the process that we are interested in,

B.3 $$\begin{aligned} \bar{q}(p_1) + q(p_2) \rightarrow V(p_V) + g(p_4) + g(p_5), \end{aligned}$$

and, as we will see, allows us to expose all the collinear singularities in a straightforward manner. We assume that momenta of quarks in the initial state point along the *z*-axis

B.4 $$\begin{aligned}&p_{1,2} = E_{1,2} \left( t^\mu \pm e_3^\mu \right) ,\quad t^\mu = (1,0,0,0;\ldots ),\nonumber \\&e_3^\mu = (0,0,0,1;0,0\ldots ), \end{aligned}$$

and parametrize the gluon momenta as

B.5 $$\begin{aligned} p_4&= E_4 \big ( t^\mu + \cos \theta _{41} e_3^\mu + \sin \theta _{41} b^\mu \big ),\nonumber \\ p_5&=E_5 \big ( t^\mu + \cos \theta _{51} e_3^\mu + \sin \theta _{51} \left( \cos \varphi _{45} b^\mu + \sin \varphi _{45} a^\mu \big ) \right) , \end{aligned}$$

The (*d*-dimensional) unit vectors $b^\mu$ and $a^\mu$ are chosen in such a way that

B.6 $$\begin{aligned} t \cdot a = e_3 \cdot a = t \cdot b = e_3 \cdot b = a \cdot b = 0. \end{aligned}$$

Given this choice, the angular phase space is written as [10, 11]

B.7 $$\begin{aligned} \mathrm{d}\Omega _{45}= & {} \frac{\mathrm{d} \Omega _{b}^{(d-2)} \mathrm{d} \Omega _{a}^{(d-3)}}{ 2^{6\epsilon } (2 \pi )^{2 d -2} } \left[ \eta _4 (1-\eta _4) \right] ^{-\epsilon } \left[ \eta _5 ( 1- \eta _5 ) \right] ^{-\epsilon }\nonumber \\&\times \frac{| \eta _4 - \eta _5|^{1-2\epsilon } }{D^{1-2\epsilon } } \frac{ \mathrm{d} \eta _4 \mathrm{d} \eta _5 \mathrm{d} \lambda }{[\lambda ( 1- \lambda )]^{1/2+\epsilon }}, \end{aligned}$$

where $\eta _{i} = \eta _{i1}$ and $\eta _{ij} = \rho _{ij}/2$, $D = \eta _4 + \eta _5 - 2 \eta _4 \eta _5 + 2 ( 2\lambda - 1) \sqrt{\eta _4 \eta _5 ( 1- \eta _4) (1 - \eta _5) }$, and

B.8 $$\begin{aligned}&\eta _{45} = \frac{|\eta _4 - \eta _5|^2}{D},\;\;\;\; \sin ^2 \varphi _{45} = 4 \lambda ( 1- \lambda ) \frac{|\eta _4 - \eta _5 |^2}{D^2}. \end{aligned}$$

The phase space can be split into four different sectors that we will refer to as *a*, *b*, *c*, *d*. The following parametrization is chosen for each of the four sectors [10, 11]:

$$\begin{aligned}&(a) \eta _4 = x_3,\quad \eta _5 = x_3 x_4/2;\quad (b) \eta _4 = x_3,\quad \eta _5 = x_3 ( 1- x_4/2);\\&(c) \eta _4 = x_3 x_4/2 ,\quad \eta _5 = x_3 ;\quad (d) \eta _4 = x_3 (1-x_4/2),\quad \eta _5 = x_3. \end{aligned}$$

Below we present the phase space for each of the four sectors employing the above parametrization. To this end, we will need the following function:

B.9 $$\begin{aligned} N(x_3,x_4, \lambda )= & {} 1 + x_4 ( 1 - 2 x_3)\nonumber \\&- 2(1-2 \lambda ) \sqrt{x_4(1-x_3) ( 1- x_3 x_4)}. \end{aligned}$$

It turns out that the angular phase spaces for sectors *a* and *c* and for sectors *b* and *d* are identical. The results read

B.10 $$\begin{aligned} \mathrm{d} \Omega _{45}^{(a,c)}&= \frac{\mathrm{d} \Omega _{b}^{(d-2)} \mathrm{d} \Omega _{a}^{(d-3)}}{ 2^{6\epsilon } (2 \pi )^{2 d -2} } \frac{\mathrm{d} x_3 }{x_3^{1+2\epsilon }}\frac{\mathrm{d} x_4 }{ x_4^{1+ \epsilon } } \frac{\mathrm{d} \lambda }{ (\lambda ( 1- \lambda ) )^{1/2+\epsilon }}\; F_{\epsilon }^{-\epsilon }\;\nonumber \\ F_0 \; x_3^2 x_4&= \left[ \frac{1}{8\pi ^2}\frac{(4\pi )^{\epsilon }}{\Gamma (1-\epsilon )}\right] ^2 \left[ \frac{\Gamma ^2(1-\epsilon )}{\Gamma (1-2\epsilon )}\right] \nonumber \\&\quad \times \left[ \frac{\mathrm{d}\Omega ^{(b)}_{d-2}}{\Omega _{d-2}} \frac{\mathrm{d}\Omega ^{(a)}_{d-3}}{\Omega _{d-3}}\right] \times \frac{\mathrm{d} x_3}{x_3^{1+2\epsilon }}\frac{\mathrm{d} x_4}{x_4^{1+ \epsilon } } \nonumber \\&\quad \times \frac{\mathrm{d} \lambda }{ \pi (\lambda ( 1- \lambda ) )^{1/2+\epsilon }}\; \; (256 F_{\epsilon })^{-\epsilon } \; 4F_0\; x_3^2 x_4,\nonumber \\ F_\epsilon&=\frac{(1-x_3) (1-x_3 x_4/2) (1-x_4/2)^2}{2 N(x_3,x_4/2,\lambda )^2},\;\;\;\;\nonumber \\ F_0&= \frac{(1-x_4/2)}{2 N(x_3,x_4/2,\lambda )},\;\;\;\Bigg . \end{aligned}$$

and

B.11 $$\begin{aligned} \mathrm{d} \Omega _{45}^{(b,d)}&= \frac{\mathrm{d} \Omega _{b}^{(d-2)} \mathrm{d} \Omega _{a}^{(d-3)}}{ 2^{6\epsilon } (2 \pi )^{2 d -2} } \frac{\mathrm{d} x_3}{x_3^{1+2\epsilon }}\frac{\mathrm{d} x_4}{ x_4^{1+ 2 \epsilon } } \frac{\mathrm{d} \lambda }{ (\lambda ( 1- \lambda ) )^{1/2+\epsilon }}\; F_{\epsilon }^{-\epsilon }\;\nonumber \\ F_0 \; x_3^2 x_4^2&= \left[ \frac{1}{8\pi ^2}\frac{(4\pi )^{\epsilon }}{\Gamma (1-\epsilon )}\right] ^2 \left[ \frac{\Gamma ^2(1-\epsilon )}{\Gamma (1-2\epsilon )}\right] \nonumber \\&\quad \times \left[ \frac{\mathrm{d}\Omega ^{(b)}_{d-2}}{\Omega _{d-2}} \frac{\mathrm{d}\Omega ^{(a)}_{d-3}}{\Omega _{d-3}}\right] \times \frac{\mathrm{d} x_3}{x_3^{1+2\epsilon }}\frac{\mathrm{d} x_4 }{ x_4^{1+ 2 \epsilon } }\nonumber \\&\quad \times \frac{\mathrm{d} \lambda }{ \pi (\lambda ( 1- \lambda ) )^{1/2+\epsilon }}\; (256 F_{\epsilon })^{-\epsilon } \;4F_0\; x_3^2 x_4^2,\nonumber \\ F_\epsilon&=\frac{(1-x_3) (1-x_4/2) (1-x_3(1-x_4/2) ) }{4 N(x_3,1-x_4/2,\lambda )^2},\;\;\;\; \nonumber \\ F_0&= \frac{1}{4 N(x_3,1-x_4/2,\lambda )}.\;\;\;\Bigg . \end{aligned}$$

Furthermore, it is beneficial to remove singular dependence on $\lambda$ in Eqs. (B.10) and (B.11) by changing variable $\lambda \rightarrow y$ as

B.12 $$\begin{aligned} \lambda = \sin ^2\left( \frac{\pi }{2}y\right) ,\quad \frac{\mathrm{d}\lambda }{\pi \sqrt{\lambda (1-\lambda )}}=\mathrm{d}y,\quad y\in (0,1). \end{aligned}$$

To deal with one singularity at a time, we have to partition the phase space. We write

B.13 $$\begin{aligned} 1 = w^{14,15} + w^{24,25} + w^{14,25} + w^{15,24}, \end{aligned}$$

where

B.14 $$\begin{aligned}&w^{14,15} = \frac{\rho _{24} \rho _{25}}{d_4 d_5} \left( 1 + \frac{\rho _{14}}{d_{4521}} + \frac{\rho _{15}}{d_{4512}} \right) ,\nonumber \\&w^{24,25} = \frac{\rho _{14} \rho _{15}}{d_4 d_5} \left( 1 + \frac{\rho _{25}}{d_{4521}} + \frac{\rho _{24}}{d_{4512}} \right) , \nonumber \\&w^{14,25} = \frac{\rho _{24} \rho _{15} \rho _{45}}{d_4 d_5 d_{4512}},\quad w^{24,15} = \frac{\rho _{14} \rho _{25} \rho _{45}}{d_4 d_5 d_{4521}}. \end{aligned}$$

We have introduced the following notation:

B.15 $$\begin{aligned} d_{i =4,5}= & {} \rho _{1i} + \rho _{2i} = 2,\quad d_{4521} = \rho _{45} + \rho _{42}+\rho _{51},\nonumber \\ d_{4512}= & {} \rho _{45} + \rho _{41}+\rho _{52}. \end{aligned}$$

In Eq. (B.13), the term $w^{14,15}$ corresponds to the triple-collinear sector where singular radiation occurs along the direction of the incoming quark with momentum $p_1$, $w^{24,25}$ to the triple-collinear sector where singular radiation occurs along the direction of the incoming antiquark with momentum $p_2$, and $w^{14,25}$ and $w^{15,24}$ to the double-collinear sectors.

## Footnotes

1 We also note that recently the CoLoRFulNNLO scheme was fully worked out for $e^+e^-$ colliders [63, 64].

2 A different improvement of the original scheme [10, 11], which also allows one to deal with four-dimensional matrix elements was presented in Ref. [12].

3 For earlier efforts, see Ref. [69].

4 This feature is particular to the process under consideration.

5 The $\mathcal O(\epsilon ^2)$ contribution to $\mathcal P_{qq,R}$, relevant for NNLO contributions, is reported in Appendix A.

6 The very possibility of regulating soft singularities independently of collinear ones follows directly from QCD color coherence. It is the primary reason why we do not need to consider sectors where soft and collinear singularities are entangled. Note that this feature does not apply to individual diagrams but to on-shell QCD scattering amplitudes as a whole.

7 In a more general NNLO problem, this integral is a function of the scalar product of the three-momenta of the two hard partons.

8 This expression is valid for sector (*b*). For sector (*d*), one should replace $r\rightarrow -\tilde{r}$ with $\tilde{r} = \sin \theta _{51} \; e_3 - \cos \theta _{51}\;b$ in Eq. (8.62).

9 For ease of notation, we will drop the sector index in the intermediate calculations and restore it at the end.

10 At this point, we restore the sector label.

11 We remind the reader that we only consider the $q \bar{q}$ annihilation channel and restrict ourselves to gluonic corrections in this paper.

12 The state-of-the-art comparison of this and other observables in Drell–Yan production between different NNLO codes was presented in Ref. [88].
